# Supplementary material for: One-Pot Access to Functionalised Malamides via Organocatalytic Enantioselective Formation of Spirocyclic β-Lactone-Oxindoles and Double Ring-Opening
Source: Molecules. 2024 Jul 31;29(15):3635. doi: 10.3390/molecules29153635 (PMC11313722; doi:10.3390/molecules29153635)

# One-pot Access to Functionalised Malamides via Organocatalytic Enantioselective Formation and Double Ring-opening of Spirocyclic β-Lactone-Oxindoles

Alastair J. Nimmo, Kevin Kasten, George White, Julia Roeterdink, Aidan P. McKay, David B. Cordes, and Andrew D. Smith*

## Supporting Information

## Table of Contents

**1. General Information** .......... **2**

**2. General Procedures** .......... **4**

2.1 General Procedure A: Preparation of Boc-protected isatins .......... 4

2.2 General Procedure B: Isothiourea-catalysed enantioselective [2+2] cycloaddition – double ring opening protocol .......... 4

**3. Preparation of isatins** .......... **5**

**4. Isothiourea catalysis products** .......... **8**

**Single crystal X-ray diffraction data** .......... **20**

**References** .......... **23**

**Appendix I: $^{1}H$ and $^{13}C\{^{1}H\}$ NMR Spectra** .......... **24**

**Appendix II: HPLC traces of novel compounds** .......... **57**

## 1. General Information

All reagents and solvents were obtained from commercial suppliers and were used without further purification unless otherwise stated. Anhydrides and *N*-Boc isatin were prepared in accordance with literature.[1,2] Reactions involving moisture sensitive reagents were carried out in flame-dried glassware under an inert atmosphere ($N_2$ or Ar) using standard vacuum line techniques. Anhydrous solvents ($Et_2O$, $CH_2Cl_2$, THF and toluene) were obtained after passing through an alumina column (Mbraun SPS-800). Petrol is defined as petroleum ether 40–60 °C.
Room temperature (RT) refers to 20–25 °C.

Under reduced pressure refers to the use of either a Büchi Rotavapor R-200 with a Büchi V-491 heating bath and Büchi V-800 vacuum controller, a Büchi Rotavapor R-210 with a Büchi V-491 heating bath and Büchi V-850 vacuum controller, a Heidolph Laborota 4001 with vacuum controller, an IKA RV10 rotary evaporator with a IKA HB10 heating bath and ILMVAC vacuum controller, or an IKA RV10 rotary evaporator with a IKA HB10 heating bath and Vacuubrand CVC3000 vacuum controller. Rotary evaporator condensers are fitted to Julabo FL601 Recirculating Coolers filled with ethylene glycol and set to –6 °C.

Analytical thin layer chromatography (TLC) was performed on pre-coated aluminium plates (Kieselgel 60 F254 silica) and visualisation was achieved using ultraviolet light (254 nm) and/or staining with aqueous $KMnO_4$ solution by heating. Manual column chromatography was performed in glass columns fitted with porosity 3 sintered discs over Kieselgel 60 silica using the solvent system stated.
Melting points were recorded on an Electrothermal 9100 melting point apparatus, (dec) refers to decomposition.

Optical rotations were measured on a Perkin Elmer Precisely/Model-341 polarimeter operating at the sodium D line with a 100 mm path cell at 20 °C. Concentrations (*c*) are stated in g/100 mL.

HPLC analyses were obtained on either a Shimadzu HPLC consisting of a DGU-20A5 degassing unit, LC-20AT liquid chromatography pump, SIL-20AHT autosampler, CMB-

20A communications bus module, SPD-M20A diode array detector and a CTO-20A column oven or a Shimadzu HPLC consisting of a DGU-20A5R degassing unit, LC-20AD liquid chromatography pump, SIL-20AHT autosampler, SPD-20A UV/Vis detector and a CTO-20A column oven. Separation was achieved using DAICEL CHIRALPAK AD-H, IA, and ID columns using the method stated. HPLC traces of enantiomerically enriched compounds were compared with authentic racemic spectra.

Infrared spectra were recorded on a Shimadzu IRAffinity-1 Fourier transform IR spectrophotometer fitted with a Specac Quest ATR accessory (diamond puck). Spectra were recorded of thin films with characteristic absorption wavenumbers ($\nu_{max}$) reported in $cm^{-1}$.

$^{1}H$, $^{13}C\{^{1}H\}$, and $^{19}F\{^{1}H\}$ NMR spectra were acquired on either a Bruker AV400 with a BBFO probe, a Bruker AVII 400 with a BBFO probe, a Bruker AVIII-HD 500 with a SmartProbe BBFO+ probe, a Bruker AVIII 500 with a CryoProbe Prodigy BBO probe, or a Bruker AVIII-HD 700 with a CryoProbe Prodigy TCI probe in the deuterated solvent stated. All chemical shifts are quoted in parts per million (ppm) relative to the residual solvent peak. All coupling constants, J, are quoted in Hz. Multiplicities are indicated as s (singlet), d (doublet), t (triplet), q (quartet), m (multiplet), and multiples thereof. The abbreviation Ar denotes aromatic. NMR peak assignments were confirmed using 2D $^{1}H$ correlated spectroscopy (COSY), 2D $^{1}H$ nuclear Overhauser effect spectroscopy (NOESY), 2D $^{1}H$–$^{13}C$ heteronuclear multiple-bond correlation spectroscopy (HMBC), and 2D $^{1}H$–$^{13}C$ heteronuclear single quantum coherence (HSQC) where necessary.

Mass spectrometry (*m*/*z*) data were acquired by either electrospray ionisation (ESI), electron impact (EI) at either the University of St Andrews Mass Spectrometry Facility or SIRCAMS at University of Edinburgh.

## 2. General Procedures

### 2.1 General Procedure A: Preparation of Boc-protected isatins

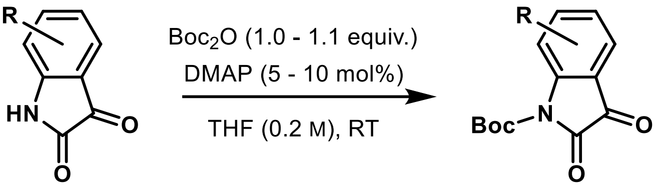


The appropriate isatin (1.0 equiv.) and di-*tert*-butyl dicarbonate (1.0 – 1.1 equiv.) were dissolved in anhydrous THF (0.2 M). DMAP (5 – 10 mol%) was then added and the reaction mixture stirred at RT. Once complete, the reaction mixture was concentrated under reduced pressure and purified directly by flash silica column chromatography and recrystallisation as specified.

### 2.2 General Procedure B: Isothiourea-catalysed enantioselective [2+2] cycloaddition – double ring opening protocol

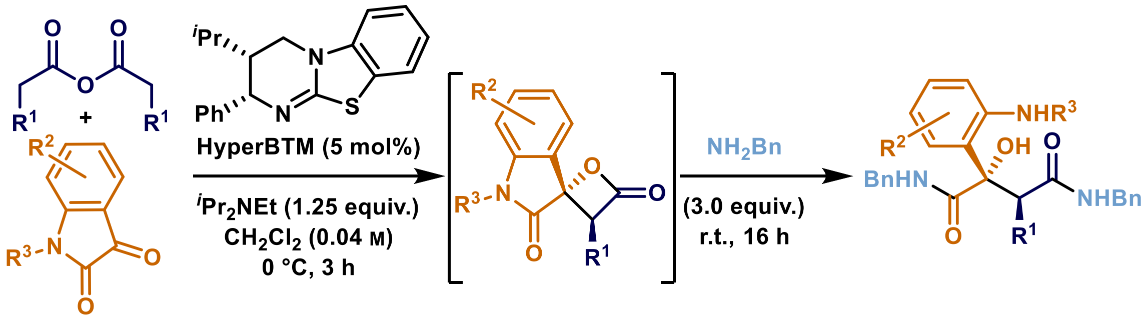


The appropriate anhydride (1.5 equiv.), protected isatin (1.0 equiv.), and (2*S*,3*R*)-HyperBTM (5 mol%) were dissolved in anhydrous $CH_2Cl_2$ (0.04 M) and cooled to 0 °C. The reaction was started by addition of $^i$Pr$_2$NEt (1.3 equiv.) and was stirred at 0 °C for 3 h then the appropriate amine (3.0 equiv.) was added, and the mixture allowed to warm to RT to stir o/n. Once complete, the crude mixture was subjected directly to flash silica column chromatography.

**3. Preparation of isatins**

**3.1 tert-butyl 5-methoxy-2,3-dioxoindoline-1-carboxylate S1**

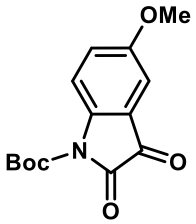


Following General Procedure A, 5-methoxyindoline-2,3-dione (0.89 g, 5.0 mmol, 1.0 equiv.), di-*tert*-butyl dicarbonate (1.20 g, 5.5 mmol, 1.1 equiv.), and DMAP (31 mg, 0.25 mmol, 5 mol%) gave crude product that was purified by flash silica column chromatography (hexane:EtOAc 100:0 to 70:30) to give the title compound (0.78 g, 56%) as an orange solid. **mp** 105-107 °C; **IR** $\nu_{max}$ (film) 2972, 1800, 1776, 1732, 1587, 1489, 1329, 1285, 1242, 1196, 1148, 1123, 1034, 1013, 997, 845; **$^1$H NMR** (400 MHz, $CDCl_3$) $\delta_H$: 1.64 (9H, s, C(C*H*$_3$)$_3$), 3.84 (3H, s, OC*H*$_3$), 7.20 (1H, d, *J* 2.8, C(4)*H*), 7.23 – 7.26 (1H, m, C(6)*H*), 8.00 (1H, d, *J* 8.9, C(7)*H*); **$^{13}$C{$^1$H} NMR** (126 MHz, $CDCl_3$) $\delta_C$: 28.2 (C(*C*H$_3$)$_3$), 56.0 (OCH$_3$), 85.5 (*C*(CH$_3$)$_3$), 107.8 (*C*(4)H), 118.4 (*C*(7)H), 119.4 (*C*(3a)), 126.3 (*C*(6)H), 142.7 (*C*(7a)), 148.5 (*C*(O)O), 156.1 (*C*(2)), 157.4 (*C*(5)), 180.5 (*C*(3)); **HRMS** (ESI$^+$) $C_{14}H_{15}O_5NNa$ [M + Na]$^+$ found 300.0835, requires 300.0842 (−2.3 ppm).

**3.2 tert-butyl 5-bromo-2,3-dioxoindoline-1-carboxylate S2**

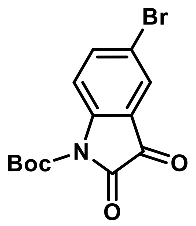


Following General Procedure A, 5-bromoindoline-2,3-dione (1.13 g, 5.0 mmol, 1.0 equiv.), di-*tert*-butyl dicarbonate (1.09 g, 5.0 mmol, 1.0 equiv.), and DMAP (61 mg, 0.50 mmol, 10 mol%) gave crude product that was purified by flash silica column chromatography (hexane:EtOAc 100:0 to 50:50) then recrystallised from heptane to give the title compound (0.64 g, 39%) as yellow crystals. **mp** 114-116 °C; **IR** $\nu_{max}$ (film) 2980, 1782, 1732, 1605, 1587, 1466, 1452, 1368, 1317, 1254, 1142, 1111, 1063, 1015, 912, 824; **$^1$H NMR** (400 MHz, $CDCl_3$) $\delta_H$: 1.64 (9H, s, C(C*H*$_3$)$_3$), 7.80 (1H, dd, *J* 8.7, 2.2, C(6)*H*), 7.84 (1H, dd, *J* 2.2, 0.5, C(4)*H*), 8.01

(1H, dd, $J$ 8.7, 0.5, C(7)$H$); $^{13}$**C{$^1$H} NMR** (126 MHz, $CDCl_3$) $\delta_C$: 27.9 (C($C$$H_3$)$_3$), 85.9 ($C$($CH_3$)$_3$), 118.6 ($C$(7)H), 118.6 ($C$(5)), 119.7 ($C$(3a)), 127.7 ($C$(4)H), 141.0 ($C$(6)H), 147.0 ($C$(7a)), 148.0 ($C$(O)O), 154.6 ($C$(2)), 179.0 ($C$(3)); **HRMS** ($EI^+$) $C_{13}H_{12}O_4NBr$ $[M]^+$ found 324.9948, requires 324.9944 (+1.2 ppm).

**tert-butyl 6-chloro-2,3-dioxoindoline-1-carboxylate S3**

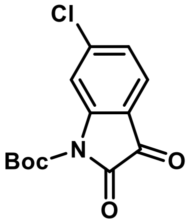


Following General Procedure A, 6-chloroindoline-2,3-dione (0.91 g, 5.0 mmol, 1.0 equiv.), di-*tert*-butyl dicarbonate (1.09 g, 5.0 mmol, 1.1 equiv.), and DMAP (61 mg, 0.5 mmol, 10 mol%) gave crude product that was purified by flash silica column chromatography (hexane:EtOAc 100:0 to 60:40) then recrystallised from heptane to give the title compound (0.40 g, 28%) as a yellow solid. **mp** 100-102 °C; **IR** $\nu_{max}$ (film) 2988, 1796, 1780, 1736, 1597, 1576, 1458, 1420, 1339, 1263, 1244, 1130, 1072, 995, 912, 880, 841, 766; $^1$**H NMR** (400 MHz, $CDCl_3$) $\delta_H$: 1.64 (9H, s, C(C$H_3$)$_3$), 7.26 (1H, dd, $J$ 8.1, 1.7, C(5)$H$), 7.67 (1H, d, $J$ 8.1, C(4)$H$), 8.16 (1H, d, $J$ 1.8, C(7)$H$); $^{13}$**C{$^1$H} NMR** (126 MHz, $CDCl_3$) $\delta_C$: 28.1 (C($C$$H_3$)$_3$), 86.2 ($C$($CH_3$)$_3$), 117.1 ($C$(3a)), 117.7 ($C$(7)H), 126.0 ($C$(4)H), 126.3 ($C$(5)H), 145.4 ($C$(7a)), 148.2 ($C$(6)), 149.1 ($C$(O)O), 155.4 ($C$(2)), 179.0 ($C$(3)); **HRMS** ($ESI^+$) $C_{13}H_{12}O_4NClNa$ $[M + Na]^+$ found 304.0340, requires 304.0347 (−2.3 ppm).

### 3.4 allyl 2,3-dioxoindoline-1-carboxylate S4

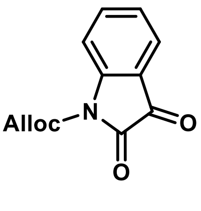


To a solution of indoline-2,3-dione (1.47 g, 10 mmol, 1.0 equiv.) and triethylamine (0.81 mL, 11 mmol, 1.1 equiv.) in anhydrous THF (0.2 M), was added dropwise allyl chloroformate (1.11 mL, 10.5 mmol, 1.05 equiv.) at 0 °C. The reaction mixture was allowed to warm to RT and stirred overnight. Once complete, the reaction was diluted with $H_2O$

and extracted with $Et_2O$. The combined organics were washed sequentially with 1 M HCl, sat. $NaHCO_3$, and brine, then dried over $MgSO_4$, filtered and concentrated. The crude product was purified by flash silica column chromatography (hexane:EtOAc 100:0 to 70:30) to give the title compound (0.35 g, 15%) as a yellow solid. **mp** 79-80 °C; **IR** $\nu_{max}$ (film) 2938, 1775, 1748, 1728, 1591, 1464, 1381, 1339, 1310, 1240, 1225, 1155, 1098, 997, 935, 889; **$^1$H NMR** (400 MHz, $CDCl_3$) $\delta_H$: 4.93 (2H, dt, *J*, 5.5, 1.4, C*H*$_2$O), 5.37 (1H, dq, *J* 10.5, 1.2, CH=C*H*$^A$H$^B$), 5.56 (1H, dq, *J* 17.2, 1.5, CH=CH$^A$C*H*$^B$), 6.05 (1H, ddt, *J* 17.2, 10.5, 5.5, C*H*=CH$_2$), 7.32 (1H, td, *J* 7.6, 0.9, C(5)*H*), 7.71 – 7.79 (2H, m, C(4)*H* and C(6)*H*), 8.15 (1H, dt, *J* 8.3, 0.8, C(7)*H*); **$^{13}$C{$^1$H} NMR** (126 MHz, $CDCl_3$) $\delta_C$: 68.3 (*C*H$_2$O), 117.1 (*C*(7)H), 119.0 (*C*(3a)), 119.9 (CH=*C*H$_2$), 125.6 (*C*(4)H), 125.9 (*C*(5)H), 130.7 (*C*H=CH$_2$), 139.1 (*C*(6)H), 148.0 (*C*(7a)), 150.0 (*C*(O)O), 155.5 (*C*(2)), 179.8 (*C*(3)); **HRMS** (EI$^+$) $C_{12}H_9O_4N$ [M]$^+$ found 231.0528, requires 231.0526 (+0.9 ppm).

## 4. Isothiourea catalysis products

### 4.1 *tert*-Butyl (2-((2*S*,3*S*)-1,4-bis(benzylamino)-2-hydroxy-1,4-dioxo-3-phenylbutan-2-yl)phenyl)carbamate 1

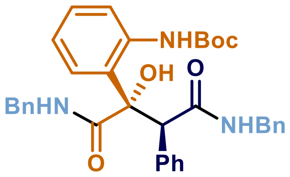


Following General Procedure B, phenylacetic anhydride (95.3 mg, 0.38 mmol, 1.5 equiv.), *tert*-butyl 2,3-dioxoindoline-1-carboxylate (61.8 mg, 0.25 mmol, 1.0 equiv.), (2*S*,3*R*)-HyperBTM (3.9 mg, 0.012 mmol, 5 mol%) and $^{i}Pr_2NEt$ (54 μL, 0.31 mmol, 1.3 equiv.) in $CH_2Cl_2$ (0.04 M), followed by benzylamine (82 μL, 0.75 mmol, 3.0 equiv.) gave crude product that was purified by flash silica column chromatography (hexane:EtOAc 90:10 to 40:60) to give the title compound as a pale yellow glass (91 mg, 77%). $[\alpha]_D^{20}$ −127.0 (*c* 2.3 in $CHCl_3$); **Chiral HPLC analysis,** Chiralpak IA (95:5 *n*-hexane : $^{i}$PrOH, flow rate 2.0 mL·min$^{-1}$, 211 nm, 40 °C) $t_R$ (2*S*,3*S*)-**1** 23.8 min, $t_R$ (2*R*,3*R*)-**1** 28.1 min, >99:1 er; **IR** $\nu_{max}$ (film) 3397, 3316, 3088, 3065, 3030, 2978, 2928, 2249, 1717, 1651, 1639, 1587, 1522, 1497, 1452, 1441, 1391, 1366, 1308, 1231, 1157, 1121, 1045, 1026, 961, 908, 831, 754; **$^{1}$H NMR** (400 MHz, $CDCl_3$) $\delta_H$: 1.46 (9H, s, OC(C$H_3$)$_3$), 4.25 (1H, dd, *J* 15.1, 5.4, NC$H_2$), 4.35 (1H, dd, *J* 15.2, 5.5, NC$H_2$), 4.47 (1H, dd, *J* 15.1, 6.2, NC$H_2$), 4.50 (1H, dd, *J* 15.2, 6.1, NC$H_2$), 4.73 (1H, br s, C(3)*H*), 6.86 (1H, dd, *J* 8.0, 7.2, Ar*H*), 7.05 (2H, d, *J* 7.4, Ar*H*), 7.11 – 7.39 (16H, m, Ar*H* and O*H*), 7.54 (1H, d, *J* 8.0, Ar*H*), 7.99 (1H, s, N*H*), 8.01 (1H, s, N*H*), 9.15 (1H, br s, N*H*); **$^{13}$C{$^{1}$H} NMR** (101 MHz, $CDCl_3$) $\delta_C$: 28.5 (OC($CH_3$)$_3$), 43.5 ($NCH_2$), 43.6 ($NCH_2$), 55.8 (*C*(2)), 79.4 (O*C*($CH_3$)$_3$), 85.0 (*C*(3)H), 122.0 (ArCH), 126.3 (ArCH), 127.3 (ArCH), 127.4 (ArCH), 127.4 (ArCH), 127.5 (ArCH), 128.0(ArCH), 128.4 (ArCH), 128.7 (ArCH), 128.9 (ArCH), 129.3 (ArCH), 133.5 (Ar*C*), 137.5 (Ar*C*), 137.6 (Ar*C*), 138.8 (Ar*C*), 152.5 (*C*=O), 174.4 (*C*=O), 175.2 (*C*=O); **HRMS** (ESI$^{+}$) $C_{25}H_{37}N_3O_5Na$ $[M + Na]^{+}$found 602.2622, requires 602.2625 (−0.5 ppm).

### 4.2 *tert*-Butyl (2-((2*S*,3*S*)-2-hydroxy-1,4-dioxo-3-phenyl-1,4-bis((pyridin-2-ylmethyl)amino)butan-2-yl)phenyl)carbamate 2

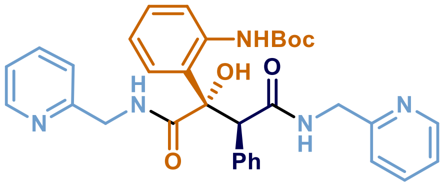


Following General Procedure B, phenylacetic anhydride (127 mg, 0.50 mmol, 1.5 equiv.), *tert*-butyl 2,3-dioxoindoline-1-carboxylate (81.6 mg, 0.33 mmol, 1.0 equiv.), (2*S*,3*R*)-HyperBTM (5.1 mg, 0.017 mmol, 5 mol%) and *i*-$Pr_2NEt$ (75 µL, 0.43 mmol, 1.3 equiv.) in $CH_2Cl_2$ (0.04 M), followed by 2-picolylamine (104 µL, 1.00 mmol, 3.0 equiv.) gave crude product that was purified by flash silica column chromatography ($CH_2Cl_2$:10% $NH_4OH$ in MeOH 99:1 to 93:7) to give the title compound as a clear glass (142 mg, 73%). $[\alpha]_D^{20}$ −138.5 (*c* 1.4 in $CHCl_3$); **Chiral HPLC analysis,** Chiralpak IA (80:20 *n*-hexane : $^i$PrOH, flow rate 1.0 mL·min$^{-1}$, 270 nm, 30 °C) $t_R$ (2*R*,3*R*)-**2** 15.9 min, $t_R$ (2*S*,3*S*)-**2** 18.5 min, 98:2 er; **IR** $\nu_{max}$ (film) 3345, 2972, 1722, 1651, 1589, 1518, 1476, 1437, 1366, 1306, 1233, 1157, 1045, 1024, 951; **$^1$H NMR** (500 MHz, $CDCl_3$) $\delta_H$: 1.50 (9H, s, OC(C*H*$_3$)$_3$), 4.48 (1H, dd, *J* 16.3, 4.9, NC*H*$_2$), 4.52 (1H, dd, *J* 16.3, 5.1, NC*H*$_2$), 4.60 (1H, dd, *J* 16.5, 5.3, NC*H*$_2$), 4.67 (1H, dd, *J* 16.5, 5.9, NC*H*$_2$), 4.76 (1H, s, C(3)*H*), 6.90 (1H, ddd, *J* 8.3, 7.2, 1.4, Ar*H*), 7.01 (2H, m, Ar*H*), 7.11 – 7.20 (9H, m, Ar*H*), 7.23 (1H, d, *J* 7.8, Ar*H*), 7.35 (1H, t, *J* 4.2, N*H*), 7.53 (1H, dd, *J* 8.1, 1.6, Ar*H*), 7.59 (1H, ddd, *J* 7.9, 7.7, 1.9, Ar*H*), 7.62 (1H, ddd, *J* 7.9, 7.8, 1.9, Ar*H*), 7.92 (1H, br s, N*H*), 8.47 (1H, ddd, *J* 4.9, 1.8, 0.9, Ar*H*), 8.52 (1H, ddd, *J* 4.9, 1.8, 1.0, Ar*H*), 9.22 (1H, br s, N*H*); **$^{13}$C{$^1$H} NMR** (126 MHz, $CDCl_3$) $\delta_C$: 28.5 (C($CH_3$)$_3$), 44.4 ($NCH_2$), 44.8 ($NCH_2$), 79.4 (O*C*($CH_3$)$_3$), 121.2 (ArCH), 121.6 (ArCH), 122.2 (ArCH), 122.4 (ArCH), 122.6 (ArC), 126.3 (ArCH), 127.8 (ArCH), 128.1 (ArCH), 128.7 (ArCH), 129.3 (ArCH), 133.5 (ArC), 136.7 (ArCH), 136.8 (ArCH), 138.9 (ArC), 148.9 (ArCH), 149.1 (ArCH), 155.4 (ArC), 156.5 (ArC), 174.8 (*C*=O), 175.0 (*C*=O); **HRMS** (ESI$^+$) $C_{33}H_{35}N_5O_5Na$ $[M + Na]^+$ found 604.2522, requires 604.2530 (−1.4 ppm).

### 4.3 *tert*-Butyl (2-((2*S*,3*S*)-2-hydroxy-1,4-dimorpholino-1,4-dioxo-3-phenylbutan-2-yl)phenyl) carbamate 3

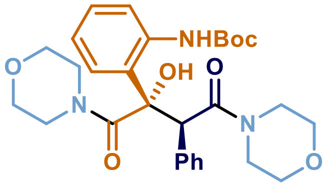


Following General Procedure B, phenylacetic anhydride (127 mg, 0.50 mmol, 1.5 equiv.), *tert*-butyl 2,3-dioxoindoline-1-carboxylate (81.6 mg, 0.33 mmol, 1.0 equiv.), (2*S*,3*R*)-HyperBTM (5.1 mg, 0.017 mmol, 5 mol%) and $^{i}Pr_2NEt$ (75 μL, 0.43 mmol, 1.3 equiv.) in $CH_2Cl_2$ (0.04 M), followed by morpholine (87 μL, 1.0 mmol, 3.0 equiv.) gave crude product that was purified by flash silica column chromatography ($CH_2Cl_2$:10% $NH_4OH$ in MeOH 99:1 to 95:5) to give the title compound as a clear glass (106 mg, 59%). $[\alpha]_D^{20}$ −176.3 (*c* 0.4 in $CHCl_3$); **Chiral HPLC analysis,** Chiralpak IA (98:2 *n*-hexane : $^{i}$PrOH, flow rate 1.5 mL·min$^{-1}$, 254 nm, 40 °C) $t_R$ (2*S*,3*S*)-**3** 10.9 min, $t_R$ (2*R*,3*R*)-**3** 21.3 min, 98:2 er; **IR** $\nu_{max}$ (film) 3339, 2855, 1722, 1632, 1614, 1587, 1529, 1439, 1366, 1304, 1242, 1227, 1157, 1111, 1045; **$^1$H NMR** (400 MHz, $CDCl_3$) $\delta_H$: 1.41 (9H, s, C(C*H*$_3$)$_3$), 2.98 – 3.06 (2H, m, OC*H*$_2$), 3.26 – 3.33 (2H, m, NC*H*$_2$), 3.44 (1H, ddd, *J* 11.4, 5.8, 2.9, OC*H*$_2$), 3.49 – 3.67 (7H, m, NC*H*$_2$ and OC*H*$_2$), 3.71 – 3.86 (4H, m, NC*H*$_2$ and OC*H*$_2$), 4.41 (1H, s, C(3)*H*), 6.82 (2H, d, *J* 7.1, Ar*H*), 6.95 (1H, ddd, *J* 8.1, 7.2, 1.3, Ar*H*), 7.08 (1H, dd, *J* 7.9, 1.6, Ar*H*), 7.13 – 7.22 (4H, m, Ar*H*), 8.03 (1H, d, *J* 8.4, Ar*H*), 8.63 (1H, s, O*H*), 8.91 (1H, s, N*H*); **$^{13}$C{$^1$H} NMR** (101 MHz, $CDCl_3$) $\delta_C$: 28.4 (C($CH_3$)$_3$), 42.3 ($NCH_2$), 43.5 ($NCH_2$), 46.2 ($NCH_2$), 47.2 ($NCH_2$), 55.1 (*C*(3)H), 66.0 ($OCH_2$), 66.5 ($OCH_2$), 66.6 ($OCH_2$), 79.2 (O*C*($CH_3$)$_3$), 86.6 (*C*(2)), 119.2 (ArCH), 121.4 (ArCH), 123.0 (ArC), 125.7 (ArCH), 128.0 (ArCH), 128.3 (ArCH), 128.9 (ArCH), 129.6 (ArCH), 132.6 (ArC), 139.3 (ArC), 151.9 (*C*=O), 171.8 (*C*=O), 174.5 (*C*=O); **HRMS** (ESI$^+$) $C_{29}H_{37}N_3O_5Na$ $[M + Na]^+$ found 562.2515, requires 562.2524 (−1.6 ppm).

### 4.4 Dimethyl 2,2'-(((2S,3S)-2-(2-((*tert*-butoxycarbonyl)amino)phenyl)-2-hydroxy-3-phenylsuccinyl)bis(azanediyl))diacetate 4

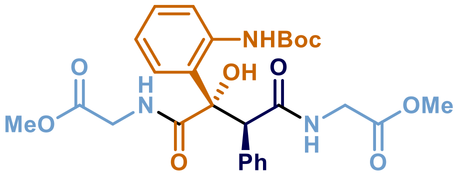


Following General Procedure B, phenylacetic anhydride (127 mg, 0.50 mmol, 1.5 equiv.), *tert*-butyl 2,3-dioxoindoline-1-carboxylate (81.6 mg, 0.33 mmol, 1.0 equiv.), (2*S*,3*R*)-HyperBTM (5.1 mg, 0.017 mmol, 5 mol%) and $^{i}Pr_2NEt$ (75 µL, 0.43 mmol, 1.3 equiv.) in $CH_2Cl_2$ (0.04 M), followed by glycine methyl ester hydrochloride (126 mg, 1.00 mmol, 3.0 equiv.) and $^{i}Pr_2NEt$ (170 µL, 1.00 mmol, 3.0 equiv.) gave crude product that was purified by flash silica column chromatography (hexane:EtOAc 100:0 to 0:100) to give the title compound as a colourless glass (50 mg, 28%). $[\alpha]_D^{20}$ −106.9 (*c* 1.5 in $CHCl_3$); **Chiral HPLC analysis,** Chiralpak IA (70:30 *n*-hexane : $^{i}$PrOH, flow rate 1.0 mL·min$^{-1}$, 254 nm, 30 °C) $t_R$ (2*S*,3*S*)-**4** 6.7 min, $t_R$ (2*R*,3*R*)-**4** 12.7 min, 99:1 er; **IR** $\nu_{max}$ (film) 3335, 2978, 1749, 1728, 1655, 1589, 1526, 1443, 1368, 1308, 1234, 1209, 1163, 1047, 1026, 756; **$^{1}$H NMR** (500 MHz, $CDCl_3$) $\delta_H$: 1.46 (9H, s, OC(C$H_3$)$_3$), 3.69 (3H, s, OC$H_3$), 3.70 (3H, s, OC$H_3$), 3.92 – 4.08 (4H, m, NC$H_2$), 4.64 (1H, br s, O*H*), 6.72 (1H, br s, N*H*), 6.84 (1H, ddd, *J* 8.2, 7.2, 1.3, Ar*H*), 6.94 – 7.00 (2H, m, Ar*H*), 7.09 – 7.19 (4H, m, Ar*H*), 7.37 (1H, br s, Ar*H*), 7.42 (1H, dd, *J* 8.1, 1.5, Ar*H*), 7.89 (1H, br s, N*H*), 9.01 (1H, br s, N*H*); **$^{13}$C{$^{1}$H} NMR** (126 MHz, $CDCl_3$) $\delta_C$: 28.6 (C($CH_3$)$_3$), 41.5 ($NCH_2$), 41.7 ($NCH_2$), 52.4 ($OCH_3$), 52.5 ($OCH_3$), 79.5 (O*C*($CH_3$)$_3$), 120.9 (ArC), 122.2 (ArCH), 126.4 (ArCH), 128.1 (ArCH), 128.2 (ArCH), 128.9 (ArCH), 129.4 (ArCH), 133.0 (ArCH), 138.5 (Ar*C*), 152.6 (Ar*C*), 169.5 (*C*=O), 169.6 (*C*=O), 174.7 (*C*=O), 175.2 (*C*=O); **HRMS** (ESI$^+$) $C_{27}H_{33}N_3O_9Na$ $[M + Na]^+$ found 566.2108, requires 566.2109 (−0.2 ppm).

### 4.5 *tert*-Butyl (2-((2*S*,3*S*)-2-hydroxy-3-(*p*-anisyl)-1,4-dimorpholino-1,4-dioxobutan-2-yl) phenyl)carbamate 5

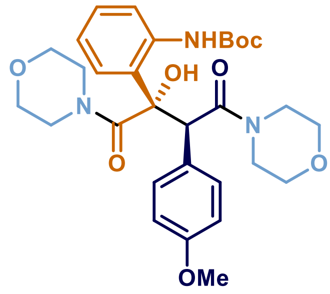


Following General Procedure B, *p*-anisylacetic anhydride (157 mg, 0.50 mmol, 1.5 equiv.), *tert*-butyl 2,3-dioxoindoline-1-carboxylate (81.6 mg, 0.33 mmol, 1.0 equiv.), (2*S*,3*R*)-HyperBTM (5.1 mg, 0.017 mmol, 5 mol%) and $^i$Pr$_2$NEt (75 μL, 0.43 mmol, 1.3 equiv.) in $CH_2Cl_2$ (0.04 M), followed by morpholine (87 μL, 1.0 mmol, 3.0 equiv.) gave crude product that was purified by flash silica column chromatography (hexane:EtOAc 70:30 to 20:80) to give the title compound as a clear glass (120 mg, 63%). $[\alpha]_D^{20}$ −108.0 (*c* 1.4 in $CHCl_3$); **Chiral HPLC analysis,** Chiralpak IA (98:2 *n*-hexane : $^i$PrOH, flow rate 1.5 mL·min$^{-1}$, 254 nm, 40 °C) $t_R$ (2*S*,3*S*)-**5** 12.3 min, $t_R$ (2*R*,3*R*)-**5** 28.1 min, 99:1 er; **IR** $\nu_{max}$ (film) 3337, 2972, 2856, 1722, 1632, 1611, 1589, 1512, 1439, 1366, 1302, 1242, 1159, 1113, 1034, 910; **$^1$H NMR** (400 MHz, $CDCl_3$) $\delta_H$: 1.41 (9H, s, C(C*H*$_3$)$_3$), 2.99 (1H, ddd, *J* 10.9, 7.5, 2.8, OC*H*$_2$), 3.05 (1H, ddd, *J* 11.1, 7.81, 2.9, OC*H*$_2$), 3.24 – 3.34 (2H, m, NC*H*$_2$), 3.40 – 3.64 (10H, m, NC*H*$_2$ and OC*H*$_2$), 3.72 (3H, s, OC*H*$_3$), 3.74 – 3.86 (2H, m, NC*H*$_2$), 4.35 (1H, s, C(3)*H*), 6.64 – 6.74 (4H, m, Ar*H*), 6.94 (1H, ddd, *J* 8.1, 7.2, 1.3, Ar*H*), 7.07 (1H, dd, *J* 7.9, 1.5, Ar*H*), 7.14 – 7.22 (1H, m, Ar*H*), 8.07 (1H, d, *J* 8.5, Ar*H*), 8.52 (1H, s, O*H*), 8.91 (1H, s, N*H*); **$^{13}$C{$^1$H} NMR** (101 MHz, $CDCl_3$) $\delta_C$: 28.3 (C($CH_3$)$_3$), 42.3 ($NCH_2$), 43.5 ($NCH_2$), 46.2 ($NCH_2$), 47.2 ($NCH_2$), 54.3 (*C*(3)H), 55.0 ($OCH_3$), 66.0 ($OCH_2$), 66.1 ($OCH_2$), 66.5 ($OCH_2$), 66.6 ($OCH_2$), 79.2 (O*C*($CH_3$)$_3$), 86.6 (*C*(2)), 113.8 (ArCH), 119.2 (ArCH), 121.4 (ArCH), 123.2 (Ar*C*), 124.3 (ArC), 125.8 (ArCH), 128.9 (ArCH), 130.7 (ArCH), 139.4 (Ar*C*), 152.1 (*C*=O), 159.1 (ArC), 171.9 (*C*=O), 174.8 (*C*=O); **HRMS** (ESI$^+$) $C_{30}H_{39}N_3O_8Na$ [M + Na]$^+$ found 592.2624, requires 592.2629 (−0.9 ppm).

### 4.6 *tert*-Butyl (2-((2*S*,3*S*)-1,4-bis(benzylamino)-3-(4-bromophenyl)-2-hydroxy-1,4-dioxobutan-2-yl)phenyl)carbamate 6

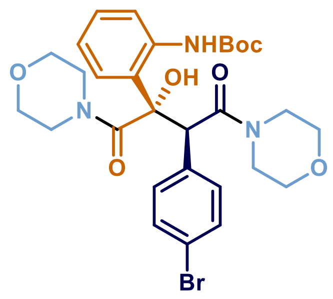


Following General Procedure B, 2-(4-bromophenyl)acetic anhydride (206 mg, 0.50 mmol, 1.5 equiv.), *tert*-butyl 2,3-dioxoindoline-1-carboxylate (81.6 mg, 0.33 mmol, 1.0 equiv.), (2*S*,3*R*)-HyperBTM (5.1 mg, 0.017 mmol, 5 mol%) and $^i$Pr$_2$NEt (75 µL, 0.43 mmol, 1.3 equiv.) in $CH_2Cl_2$ (0.04 M) followed by benzylamine (109 µL, 1.00 mmol, 3.0 equiv.) gave crude product that was purified by flash silica column chromatography (hexane:EtOAc 100:0 to 70:30) to give the combined *anti* and *syn* diastereoisomers (80:20 dr) (88 mg, 41%) as an inseparable mixture as a pale yellow glass. $[\alpha]_D^{20}$ −56.2 (*c* 1.3 in $CHCl_3$); **Chiral HPLC analysis,** Chiralpak AD-H (70:30 *n*-hexane : $^i$PrOH, flow rate 1.0 mL·min$^{-1}$, 211 nm, 30 °C) $t_R$ (2*R*,3*R*)-**6** 9.7 min, $t_R$ (2*S*,3*S*)-**6** 19.7 min, 97:3 er; $t_R$ (2*S*,3*R*)-**6** 11.4 min, $t_R$ (2*R*,3*S*)-**6** 15.0 min, 77:23 er; **IR** $\nu_{max}$ (film) 3312, 3030, 2976, 2930, 2357, 2320, 1717, 1647, 1587, 1522, 1489, 1443, 1366, 1308, 1234, 1161, 1047, 1026, 1013; **HRMS** (ESI$^+$) $C_{35}H_{36}O_5N_3BrNa$ [M + Na]$^+$found 680.1712, requires 680.1731 (−2.8 ppm); NMR data for major diastereoisomer: **$^1$H NMR** (500 MHz, $CDCl_3$) $\delta_H$: 1.46 (9H, s, C(C*H*$_3$)$_3$), 4.16 – 4.53 (4H, m, C*H*$_2$N), 4.66 (1H, br s, O*H*), 6.77 – 6.90 (4H, m, Ar*H*), 7.06 – 7.29 (14H, m, Ar*H*), 7.99 (2H, br s, 2 × N*H*), 9.12 (1H, br s, N*H*); **$^{13}$C{$^1$H} NMR** (126 MHz, $CDCl_3$) $\delta_C$: 28.6 (C(CH$_3$)$_3$), 43.6 (CH$_2$N), 43.6 (CH$_2$N), 55.4 (*C*(3)H), 79.7 (*C*(CH$_3$)$_3$), 86.6 (*C*(2)), 122.3 (ArCH), 126.2 (ArCH), 127.1 (ArCH), 127.3 (ArCH), 127.3 (ArCH), 127.4 (ArCH), 127.6 (ArCH), 127.7 (ArCH), 128.7 (ArCH), 128.8 (ArCH), 129.3 (ArC), 131.0 (ArCH), 131.3 (ArCH), 131.7(ArC), 132.0 (ArC), 132.7 (ArC), 136.9 (ArC), 137.3 (ArC), 137.5 (ArC), 152.5 (*C*=O), 174.4 (*C*=O), 174.8 (*C*=O); NMR data for minor diastereoisomer: **$^1$H NMR** (500 MHz, $CDCl_3$) (*selected*) $\delta_H$: 1.52 (9H, s, C(C*H*$_3$)$_3$), 3.81 – 3.88 (1H, m, NC*H*$_2$), 4.00 – 4.07 (1H, m, NC*H*$_2$), 4.97 – 4.97 (1H, m, NC*H*$_2$), 6.57 – 6.62 (2H, m, Ar*H*), 7.34 – 7.39 (1H, m, Ar*H*), 7.65 – 7.70 (1H, m, Ar*H*), 7.74 – 7.78 (1H, m, Ar*H*), 10.03 (1H, br s, N*H*); **$^{13}$C{$^1$H} NMR** (126 MHz, $CDCl_3$) (*selected*) $\delta_C$: 28.7 (C(CH$_3$)$_3$), 43.1 (CH$_2$N), 43.7 (CH$_2$N), 80.4 (*C*(CH$_3$)$_3$), 86.6 (*C*(2)),

122.7 (ArCH), 125.1 (ArCH), 128.7 (ArCH), 133.1 (ArC), 136.2 (ArC), 155.0 (C=O), 171.8 (C=O), 172.4 (C=O).

### 4.7 *tert*-Butyl (2-((2*S*,3*S*)-2-hydroxy-1,4-dimorpholino-1,4-dioxo-3-(thiophen-3-yl)butan-2-yl)phenyl) carbamate 7

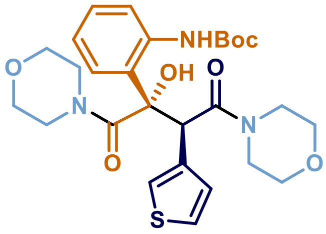


Following General Procedure B, (thiophen-3-yl)acetic anhydride (133 mg, 0.50 mmol, 1.5 equiv.), *tert*-butyl 2,3-dioxoindoline-1-carboxylate (81.6 mg, 0.33 mmol, 1.0 equiv.), (2*S*,3*R*)-HyperBTM (5.1 mg, 0.017 mmol, 5 mol%) and $^{i}Pr_2NEt$ (75 μL, 0.43 mmol, 1.3 equiv.) in $CH_2Cl_2$ (0.04 M), followed by morpholine (87 μL, 1.0 mmol, 3.0 equiv.) gave crude product that was purified by flash silica column chromatography (hexane:EtOAc 50:50 to 10:90) to give the title compound as a clear glass (73 mg, 40%). $[\alpha]_D^{20}$ −151.8 (*c* 0.9 in $CHCl_3$); **Chiral HPLC analysis,** Chiralpak IA (98:2 *n*-hexane : $^{i}$PrOH, flow rate 1.5 mL·min$^{-1}$, 211 nm, 40 °C) $t_R$ (2*S*,3*S*)-**7** 13.1 min, $t_R$ (2*R*,3*R*)-**7** 26.5 min, 98:2 er; **IR** $\nu_{max}$ (film) 3335, 2857, 1721, 1632, 1614, 1587, 1530, 1439, 1365, 1233, 1157, 1111, 1045, 995; **$^1$H NMR** (400 MHz, $CDCl_3$) $\delta_H$: 1.45 (9H, s, OC(C$H_3$)$_3$), 3.00 (1H, ddd, *J* 10.8, 7.4, 2.8, OC$H_2$), 3.12 (1H, ddd, *J* 11.1, 7.8, 2.9, OC$H_2$), 3.26 – 3.36 (2H, m, NC$H_2$), 3.41 – 3.46 (1H, m, OC$H_2$), 3.50 – 3.63 (6H, m, NC$H_2$ and OC$H_2$), 3.67 – 3.87 (5H, m, NC$H_2$ and OC$H_2$), 4.55 (1H, s, C(3)*H*), 6.43 (1H, dd, *J* 5.0, 1.3, Ar*H*), 6.82 (1H, dd, *J* 3.1, 1.3, Ar*H*), 6.95 (1H, ddd, *J* 8.1, 7.2, 1.3, Ar*H*), 7.05 (1H, dd, *J* 7.9, 1.5, Ar*H*), 7.12 (1H, dd, *J* 4.9, 3.0, Ar*H*), 7.21 (1H, ddd, *J* 8.5, 7.1, 1.5, Ar*H*), 8.12 (1H, d, *J* 8.4, Ar*H*), 8.66 (1H, s, O*H*), 9.06 (1H, s, N*H*); **$^{13}$C{$^1$H} NMR** (101 MHz, $CDCl_3$) $\delta_C$: 28.4 (C($CH_3$)$_3$), 42.3 ($NCH_2$), 43.5 ($NCH_2$), 46.3 ($NCH_2$), 47.1 ($NCH_2$), 50.5 (*C*(3)H), 66.0 ($OCH_2$), 66.1 ($OCH_2$), 66.5 ($OCH_2$), 66.6 ($OCH_2$), 79.4 (O*C*($CH_3$)$_3$), 86.3 (*C*(2)), 119.3 (ArCH), 121.5 (ArCH), 123.3 (Ar*C*), 124.5 (ArCH), 125.4 (ArCH), 125.6 (ArCH), 128.3 (ArCH), 129.0 (ArCH), 132.7 (Ar*C*), 139.4 (Ar*C*), 152.3 (*C*=O), 171.6 (*C*=O), 174.6 (*C*=O); **HRMS** (ESI$^+$) $C_{27}H_{35}N_3O_7SNa$ [M + Na]$^+$ found 568.2083, requires 568.2088 (−0.9 ppm).

### 4.8 *tert*-Butyl (2-((2*S*,3*S*,*E*)-1-(benzylamino)-3-(benzylcarbamoyl)-2-hydroxy-6-methyl-1-oxohept-4-en-2-yl)phenyl)carbamate 8

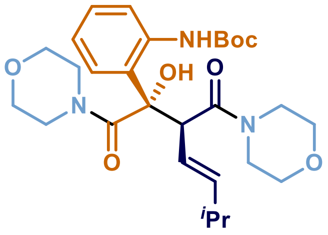


Following General Procedure B, (*E*)-5-methylhex-3-enoic anhydride (119 mg, 0.50 mmol, 1.5 equiv.), *tert*- butyl 2,3-dioxoindoline-1-carboxylate (81.6 mg, 0.33 mmol, 1.0 equiv.), (2*S*,3*R*)-HyperBTM (5.1 mg, 0.017 mmol, 5 mol%) and $^i$Pr₂NEt (75 μL, 0.43 mmol, 1.3 equiv.) in $CH_2Cl_2$ (0.04 M) followed by benzylamine (109 μL, 1.00 mmol, 3.0 equiv.) gave crude product that was purified by flash silica column chromatography (hexane:EtOAc 100:0 to 70:30) to give the title compound (93 mg, 49%) as a colourless solid. **mp** 80-82 °C; $[\alpha]_D^{20}$ −89.7 (*c* 0.7 in $CHCl_3$); **Chiral HPLC analysis,** Chiralpak IA (97:3 *n*-hexane : $^i$PrOH, flow rate 1.0 mL·min$^{-1}$, 211 nm, 40 °C) $t_R$ (2*S*,3*S*)-**8** 30.6 min, $t_R$ (2*R*,3*R*)-**8** 34.4 min, 97:3 er; **IR** $\nu_{max}$ (film) 3312, 2961, 2928, 2359, 2344, 1732, 1717, 1636, 1589, 1558, 1522, 1437, 1364, 1306, 1234, 1161, 1045, 1026, 972; **$^1$H NMR** (400 MHz, $CDCl_3$) $\delta_H$: 0.75 (3H, d, *J* 6.7, CHC*H*₃), 0.80 (3H, d, *J* 6.8, CHC*H*₃), 1.53 (9H, s, C(C*H*₃)₃), 2.06 – 2.16 (1H, m, C*H*(CH₃)₂), 4.08 – 4.17 (1H, m, C(3)*H*), 4.25 – 4.38 (2H, m, C*H*$^A$H$^B$N and CH$^A$*H*$^B$N), 4.46 (1H, dd, *J* 15.1, 6.3, C*H*$^A$H$^B$N), 4.56 (1H, dd, *J* 15.1, 6.3, CH$^A$*H*$^B$N), 5.34 (1H, dd, *J* 15.5, 6.5, C(5)*H*), 5.45 (1H, dd, *J* 15.5, 8.7, C(4)*H*), 6.68 (1H, br s, N*H*), 6.93 (1H, t, *J* 7.4, Ar*H*), 7.11 – 7.16 (2H, m, Ar*H*), 7.21 – 7.36 (9H, m, Ar*H*), 7.40 (1H, br s, N*H*), 7.57 (2H, d, *J* 8.1, Ar*H*), 8.07 (1H, br s, O*H*), 9.76 (1H, br s, N*H*); **$^{13}$C{$^1$H} NMR** (126 MHz, $CDCl_3$) $\delta_C$: 21.8 (CHCH₃), 22.1 (CHCH₃), 28.6 (C(CH₃)₃), 31.2 (CH(CH₃)₂), 43.4 (CH₂N), 43.5 (CH₂N), 53.6 (*C*(3)H), 79.9 (*C*(CH₃)₃), 83.1 (*C*(2)OH), 119.4 (*C*(4)H), 121.7 (Ar*C*), 122.6 (2 × ArCH), 126.4 (ArCH), 127.3 (ArCH), 127.4 (ArCH), 127.5 (ArCH), 127.7 (ArCH), 128.6 (ArCH), 128.7 (ArCH), 128.8 (ArCH), 137.5 (ArC), 137.6 (Ar*C*), 137.9 (Ar*C*), 144.5 (*C*(5)H), 153.2 (*C*=O), 174.6 (*C*=O), 175.5 (*C*=O); **HRMS** (ESI$^+$) $C_{34}H_{41}O_5N_3Na$ [M + Na]$^+$found 594.2928, requires 594.2938 (−1.7 ppm).

### 4.9 *tert*-Butyl (2-((2*S*,3*S*)-1,4-bis(benzylamino)-2-hydroxy-1,4-dioxo-3-phenylbutan-2-yl)-4-methoxyphenyl)carbamate 9

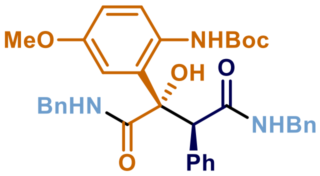


Following General Procedure B, phenylacetic anhydride (127 mg, 0.50 mmol, 1.5 equiv.), *tert*-butyl 5-methoxy-2,3-dioxoindoline-1-carboxylate (91.5 mg, 0.33 mmol, 1.0 equiv.), (2*S*,3*R*)-HyperBTM (5.1 mg, 0.017 mmol, 5 mol%) and $^{i}Pr_2NEt$ (75 μL, 0.43 mmol, 1.3 equiv.) in $CH_2Cl_2$ (0.04 M) followed by benzylamine (109 μL, 1.00 mmol, 3.0 equiv.) gave crude product that was purified by flash silica column chromatography (hexane:EtOAc 95:5 to 50:50) to give the title compound (160 mg, 80%) as a colourless solid. **mp** 78-80 °C; $[\alpha]_D^{20}$ −116.8 (*c* 1.0 in $CHCl_3$); **Chiral HPLC analysis,** Chiralpak IA (90:10 *n*-hexane : $^{i}PrOH$, flow rate 1.0 mL·min$^{-1}$, 211 nm, 30 °C) $t_R$ (2*S*,3*S*)-**9** 29.0 min, $t_R$ (2*R*,3*R*)-**9** 46.5 min, >99:1 er; **IR** $\nu_{max}$ (film) 3318, 3063, 3030, 2976, 2932, 1719, 1653, 1522, 1454, 1412, 1366, 1288, 1227, 1163, 1042, 1026, 810; **$^1$H NMR** (400 MHz, $CDCl_3$) $\delta_H$: 1.45 (9H, s, C(C*H*$_3$)$_3$), 3.63 (3H, s, OC*H*$_3$), 4.25 (1H, dd, *J* 15.1, 5.4, C*H*$^A$H$^B$N), 4.34 (1H, dd, *J* 15.1, 5.5, CH$^A$*H*$^B$N), 4.40 – 4.53 (2H, m, C*H*$_2$N), 4.76 (1H, br s, O*H*), 6.72 (1H, dd, *J* 9.1, 3.0, Ar*H*), 7.05 – 7.19 (10H, m, Ar*H*), 7.19 – 7.29 (7H, m, Ar*H*), 7.29 – 7.38 (1H, m, N*H*), 7.81 (1H, br s, N*H*), 8.95 (1H, br s, N*H*); **$^{13}$C{$^1$H} NMR** (126 MHz, $CDCl_3$) $\delta_C$: 28.6 (C(*C*H$_3$)$_3$), 43.5 (*C*H$_2$N), 43.5 (*C*H$_2$N), 55.4 (O*C*H$_3$), 77.4 (*C*H), 79.3 (*C*(CH$_3$)$_3$), 83.6 (*C*OH), 112.0 (Ar*C*H), 113.7 (Ar*C*H), 122.4 (Ar*C*), 127.3 (Ar*C*H), 127.4 (Ar*C*H), 127.5 (Ar*C*H), 127.5 (Ar*C*H), 128.0 (Ar*C*H), 128.2 (Ar*C*H), 128.7 (Ar*C*H), 128.7 (Ar*C*H), 129.4 (Ar*C*H), 131.4 (Ar*C*), 133.5 (Ar*C*), 137.4 (Ar*C*), 137.6 (Ar*C*), 152.9 (*C*=O), 154.8 (Ar*C*), 174.6 (*C*=O), 175.1 (*C*=O); **HRMS** (ESI$^+$) $C_{36}H_{39}O_6N_3Na$ [M + Na]$^+$found 632.2721, requires 632.2731 (−1.6 ppm).

### 4.10 *tert*-Butyl (2-((2*S*,3*S*)-1,4-bis(benzylamino)-2-hydroxy-1,4-dioxo-3-phenylbutan-2-yl)-4-bromophenyl)carbamate 10

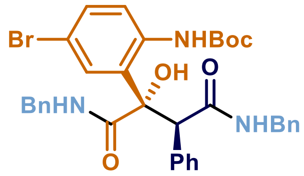


Following General Procedure B, phenylacetic anhydride (127 mg, 0.50 mmol, 1.5 equiv.), *tert*-butyl 5-bromo-2,3-dioxoindoline-1-carboxylate (108 mg, 0.33 mmol, 1.0 equiv.), (2*S*,3*R*)-HyperBTM (5.1 mg, 0.017 mmol, 5 mol%) and $^{i}Pr_2NEt$ (75 μL, 0.43 mmol, 1.3 equiv.) in $CH_2Cl_2$ (0.04 M) followed by benzylamine (109 μL, 1.00 mmol, 3.0 equiv.) gave crude product that was purified by flash silica column chromatography (hexane:EtOAc 100:0 to 70:30) to give the combined *anti* and *syn* diastereoisomers (94:6 dr) (182 mg, 84%) as an inseparable mixture as a colourless solid. **mp** 77-79 °C; $[\alpha]_D^{20}$ −88.4 (*c* 0.9 in $CHCl_3$); **Chiral HPLC analysis,** Chiralpak IA (90:10 *n*-hexane : $^{i}$PrOH, flow rate 1.0 mL·min$^{-1}$, 211 nm, 30 °C) $t_R$ (2*S*,3*S*)-**10** 12.2 min, $t_R$ (2*R*,3*R*)-**10** 30.7 min, 99:1 er; **IR** $\nu_{max}$ (film) 3316, 3030, 2978, 2928, 2357, 2320, 1717, 1647, 1454, 1393, 1366, 1300, 1233, 1161, 1049, 1026, 824; **$^1$H NMR** (500 MHz, $CDCl_3$) $\delta_H$: 1.44 (9H, s, C(C$H_3$)$_3$), 4.21 – 4.44 (3H, m, C$H_2$N), 4.51 (1H, dd, *J* 15.1, 6.2, C$H^A$$H^B$N), 4.67 (1H, br s, C(3)*H*), 7.04 – 7.33 (17H, m, Ar*H*), 7.68 (1H, d, *J* 2.4, Ar*H*), 7.90 (1H, br s, N*H*), 8.07 (1H, br s, N*H*), 9.03 (1H, br s, N*H*); **$^{13}$C{$^1$H} NMR** (126 MHz, $CDCl_3$) $\delta_C$: 28.5 (C($CH_3$)$_3$), 43.5 ($CH_2$N), 43.6 ($CH_2$N), 56.5 (*C*(3)H), 79.7 (*C*($CH_3$)$_3$), 84.9 (*C*OH), 114.8 (Ar*C*), 121.3 (ArCH), 127.4 (2 × ArCH), 127.6 (ArCH), 127.6 (ArCH), 128.2 (ArCH), 128.4 (ArCH), 128.7 (ArCH), 128.8 (ArCH), 129.4 (ArCH), 131.8 (Ar*C*), 133.2 (Ar*C*), 137.3 (Ar*C*), 137.5 (Ar*C*), 152.2 (*C*=O), 174.0 (*C*=O), 175.0 (*C*=O); **HRMS** (ESI$^+$) $C_{35}H_{36}O_5N_3BrNa$ [M + Na]$^+$found 680.1723, requires 680.1731 (−1.2 ppm).

### 4.11 *tert*-Butyl (2-((2*S*,3*S*)-1,4-bis(benzylamino)-2-hydroxy-1,4-dioxo-3-phenylbutan-2-yl)-5-chlorophenyl)carbamate 11

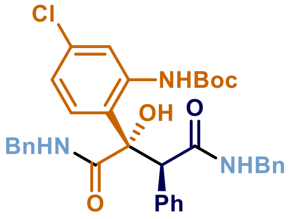


Following General Procedure B, phenylacetic anhydride (127 mg, 0.50 mmol, 1.5 equiv.), *tert*-butyl 6-chloro-2,3-dioxoindoline-1-carboxylate (93.0 mg, 0.33 mmol, 1.0 equiv.), (2*S*,3*R*)-HyperBTM (5.1 mg, 0.017 mmol, 5 mol%) and $^{i}Pr_2NEt$ (75 μL, 0.43 mmol, 1.3 equiv.) in $CH_2Cl_2$ (0.04 M) followed by benzylamine (109 μL, 1.00 mmol, 3.0 equiv.) gave crude product that was purified by flash silica column chromatography (hexane:EtOAc 100:0 to 70:30) to give the combined *anti* and *syn* diastereoisomers (95:5 dr) (160 mg, 79%) as an inseparable mixture as a colourless solid. **mp** 83-85 °C; $[\alpha]_D^{20}$ −109.0 (*c* 0.7 in $CHCl_3$); **Chiral HPLC analysis,** Chiralpak ID (90:10 *n*-hexane : $^{i}$PrOH, flow rate 1.0 mL·min$^{-1}$, 254 nm, 30 °C) $t_R$ (2*S*,3*S*)-**11** 10.1 min, $t_R$ (2*R*,3*R*)-**11** 28.6 min, 99:1 er; **IR** $\nu_{max}$ (film) 3319, 3063, 3030, 2978, 2930, 1730, 1717, 1653, 1578, 1522, 1454, 1414, 1366, 1281, 1233, 1161, 1051, 1028, 860; **$^1$H NMR** (500 MHz, $CDCl_3$) $\delta_H$: 1.46 (9H, s, C(C*H*$_3$)$_3$), 4.27 (1H, dd, *J* 15.0, 5.5, C*H*$^A$H$^B$N), 4.36 (1H, dd, *J* 15.1, 5.7, CH$^A$*H*$^B$N), 4.41 – 4.52 (2H, m, C*H*$_2$N), 4.61 (1H, br s, C*H*), 6.83 (1H, dd, *J* 8.6, 2.2, Ar*H*), 6.99 – 7.07 (2H, m, Ar*H*), 7.11 – 7.18 (4H, m, Ar*H*), 7.18 – 7.36 (10H, m, Ar*H*), 7.44 – 7.50 (1H, m, Ar*H*), 8.12 (2H, br s, N*H*), 9.14 (1H, br s, N*H*); **$^{13}$C{$^1$H} NMR** (126 MHz, $CDCl_3$) $\delta_C$: 28.5 (C(*C*H$_3$)$_3$), 43.5 (*C*H$_2$N), 43.6 (*C*H$_2$N), 56.4 (*C*H), 79.8 (*C*(CH$_3$)$_3$), 84.8 (*C*OH), 121.8 (Ar*C*H), 127.3 (Ar*C*H), 127.4 (Ar*C*H), 127.6 (Ar*C*H), 127.6 (Ar*C*H), 128.2 (Ar*C*H), 128.4 (Ar*C*H), 128.7 (2 × Ar*C*H), 129.3 (Ar*C*H), 133.2 (Ar*C*), 134.8 (Ar*C*), 137.3 (Ar*C*), 137.5 (Ar*C*), 140.2 (Ar*C*), 152.1 (*C*=O), 174.0 (*C*=O), 175.0 (*C*=O); **HRMS** (ESI$^+$) $C_{35}H_{36}O_5N_3ClNa$ [M + Na]$^+$found 636.2232, requires 636.2236 (−0.6 ppm).

### 4.12 Allyl (2-((2*S*,3*S*)-1,4-bis(benzylamino)-2-hydroxy-1,4-dioxo-3-phenylbutan-2-yl)phenyl)carbamate 12

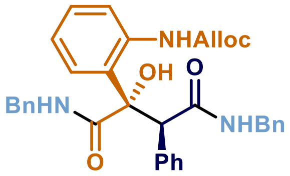


Following General Procedure B, phenylacetic anhydride (127 mg, 0.50 mmol, 1.5 equiv.), allyl 2,3-dioxoindoline-1-carboxylate (76.3 mg, 0.33 mmol, 1.0 equiv.), (2*S*,3*R*)-HyperBTM (5.1 mg, 0.017 mmol, 5 mol%) and $^{i}Pr_2NEt$ (75 μL, 0.43 mmol, 1.3 equiv.) in $CH_2Cl_2$ (0.04 M) followed by benzylamine (109 μL, 1.00 mmol, 3.0 equiv.) gave crude product that was purified by flash silica column chromatography (hexane:EtOAc 90:10 to 70:30) to give the combined *anti* and *syn* diastereoisomers (92:8 dr) (143 mg, 77%) as an inseparable mixture as a colourless solid. **mp** 73-75 °C; $[\alpha]_D^{20}$ −115.6 (*c* 1.0 in $CHCl_3$); **Chiral HPLC analysis,** Chiralpak ID (60:40 *n*-hexane : $^{i}$PrOH, flow rate 1.0 mL·min$^{-1}$, 254 nm, 40 °C) $t_R$ (2*S*,3*S*)-**12** 6.4 min, $t_R$ (2*R*,3*R*)-**12** 12.3 min, >99:1 er; **IR** $\nu_{max}$ (film) 3316, 3063, 3030, 1732, 1717, 1647, 1589, 1526, 1447, 1308, 1219, 1045; **$^1$H NMR** (400 MHz, $CDCl_3$) $\delta_H$: 4.26 (1H, dd, *J* 15.2, 5.5, C$H^AH^B$N), 4.33 (1H, dd, *J* 15.2, 5.9, C$H^AH^B$N), 4.40 – 4.58 (4H, m, C$H_2$O and C$H_2$N), 4.72 (1H, br s, C*H*), 5.22 – 5.28 (1H, m, CH=C5.31 – 5.38 (1H, m, CH=C6.87 – 6.93 (1H, m, Ar*H*), 7.00 – 7.06 (2H, m, Ar*H*), 7.11 – 7.31 (14H, m, Ar*H*), 7.33 – 7.46 (1H, m, N*H*), 7.52 – 7.59 (1H, m, Ar*H*), 7.89 – 7.96 (1H, m, Ar*H*), 8.09 (1H, br s, N*H*), 9.38 (1H, br s, N*H*); **$^{13}$C{$^1$H} NMR** (126 MHz, $CDCl_3$) $\delta_C$: 43.4 ($CH_2$N), 43.5 ($CH_2$N), 56.2 (CH), 65.3 ($CH_2$O), 85.0 (*C*OH), 117.6 (CH=$CH_2$), 120.6 (ArCH), 122.6 (ArCH), 126.3 (ArC), 127.3 (ArCH), 127.4 (ArCH), 127.5 (ArCH), 127.5 (ArCH), 127.8 (ArCH), 128.3 (ArCH), 128.7 (2 × ArCH), 129.0 (ArCH), 129.3 (ArCH), 133.0 (CH=$CH_2$), 133.5 (ArC), 137.4 (ArC), 137.6 (ArC), 152.9 (*C*=O), 174.3 (*C*=O), 175.1 (*C*=O); **HRMS** (ESI$^+$) $C_{34}H_{33}O_5N_3Na$ $[M + Na]^+$found 586.2306, requires 586.2312 (−1.1 ppm).

**Single crystal X-ray diffraction data**

X-ray diffraction data for compound **2** were collected at 125 K using a Rigaku MM-007HF High Brilliance RA generator/confocal optics [Cu K$\alpha$ radiation ($\lambda$ = 1.54187 Å)] with XtaLAB P200 diffractometer. The intensity data were collected (using a calculated strategy) and processed (including correction for Lorentz, polarization and absorption) using CrysAlisPro.[3] The structure was solved by dual-space methods (SHELXT)[4] and refined by full-matrix least-squares against $F^2$ (SHELXL-2019/3).[5] Non-hydrogen atoms were refined anisotropically, and hydrogen atoms were refined using a riding model, except for those bound to nitrogen or oxygen which were located from the difference Fourier map and refined isotropically subject to a distance restraint. All calculations were performed using the Olex2[6] interface. Selected crystallographic data are presented in Table **S1**. CCDC 2364405contains the supplementary crystallographic data for this paper. These data can be obtained free of charge from The Cambridge Crystallographic Data Centre via www.ccdc.cam.ac.uk/structures.

Table **S1**: X-ray data for **2**

| | (2*S*,3*S*)-**2** |
|---|---|
| CCDC | 2364405 |
| formula | $C_{33}H_{35}N_5O_5$ |
| fw | 581.66 |
| crystal description | Colourless needle |
| crystal size [mm$^3$] | 0.32×0.04×0.01 |
| space group | *C*2 |
| *a* [Å] | 18.3444(4) |
| *b* [Å] | 8.72591(13) |
| *c* [Å] | 18.8351(3) |
| $\beta$ [°] | 91.4776(16) |
| vol [Å]$^3$ | 3013.96(9) |
| *Z* | 4 |
| $\rho$ (calc) [g/cm$^3$] | 1.282 |
| $\mu$ [mm$^{-1}$] | 0.713 |
| F(000) | 1232 |
| reflections collected | 25846 |
| independent reflections ($R_{\text{int}}$) | 6070 (0.0696) |
| parameters, restraints | 407, 5 |
| GoF on $F^2$ | 1.091 |
| $R_1$ [$I > 2\sigma(I)$] | 0.0591 |
| $wR_2$ (all data) | 0.1854 |
| largest diff. peak/hole [e/Å$^3$] | 0.354, -0.280 |

**Datablock 2** - ellipsoid plot

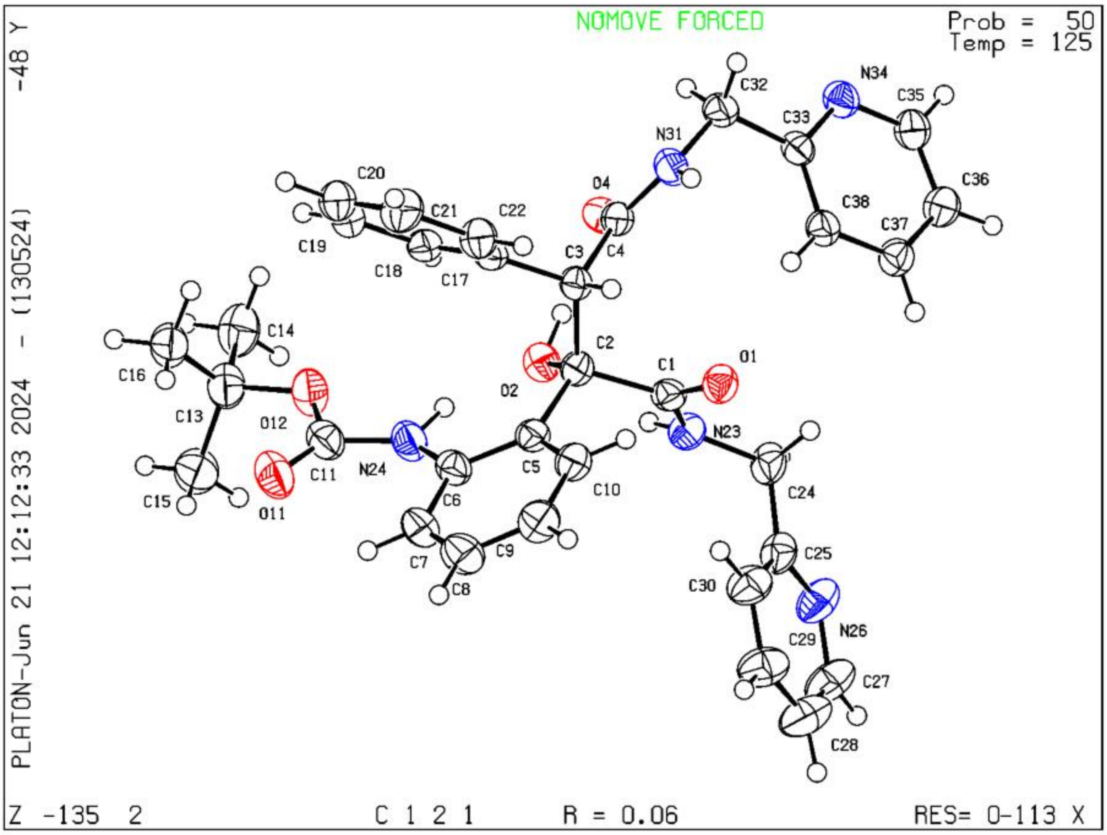


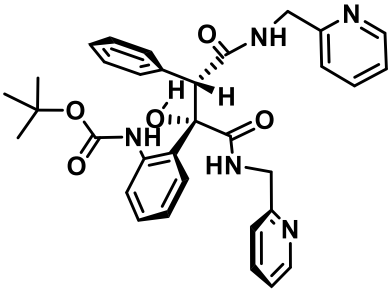

Figure **S1**: ORTEP and Sketch of X-Ray of **2**.

## References


1. Y. Abdelhamid, K. Kasten, J. Dunne, W. C. Hartley, C. M. Young, D. B. Cordes, A. M. Z. Slawin, S. Ng and A. D. Smith, *Org. Lett.*, 2022, **24**, 5444-5449.
2. G. Wille and W. Steglich, *Synthesis*, 2001, **2001**, 759-762.
3. *Journal*, 2020-2023.
4. G. Sheldrick, *Acta Crystallogr. A*, 2015, **71**, 3-8.
5. G. Sheldrick, *Acta Crystallogr. C*, 2015, **71**, 3-8.
6. O. V. Dolomanov, L. J. Bourhis, R. J. Gildea, J. A. K. Howard and H. Puschmann, *J. Appl. Crystallogr.*, 2009, **42**, 339-341.

# Appendix I: $^{1}H$ and $^{13}C\{^{1}H\}$ NMR Spectra

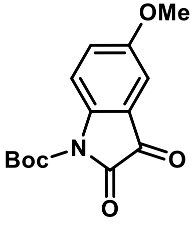


**S1**

[1]H NMR, $CDCl_3$, 400 MHz

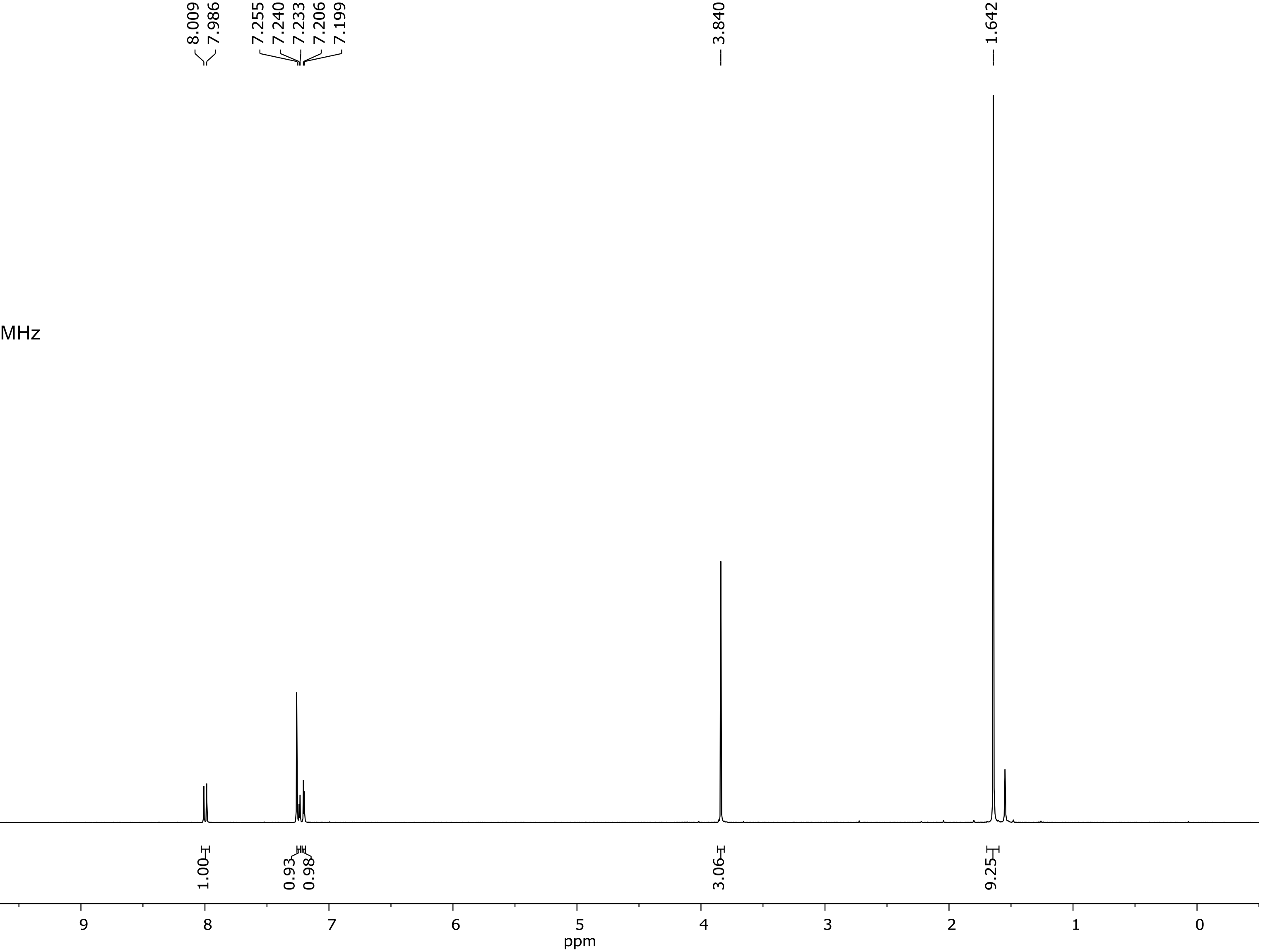

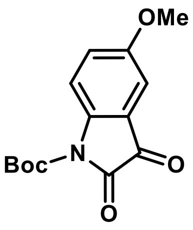


**S1**

$^{13}C\{^{1}H\}$ NMR, $CDCl_3$, 126 MHz

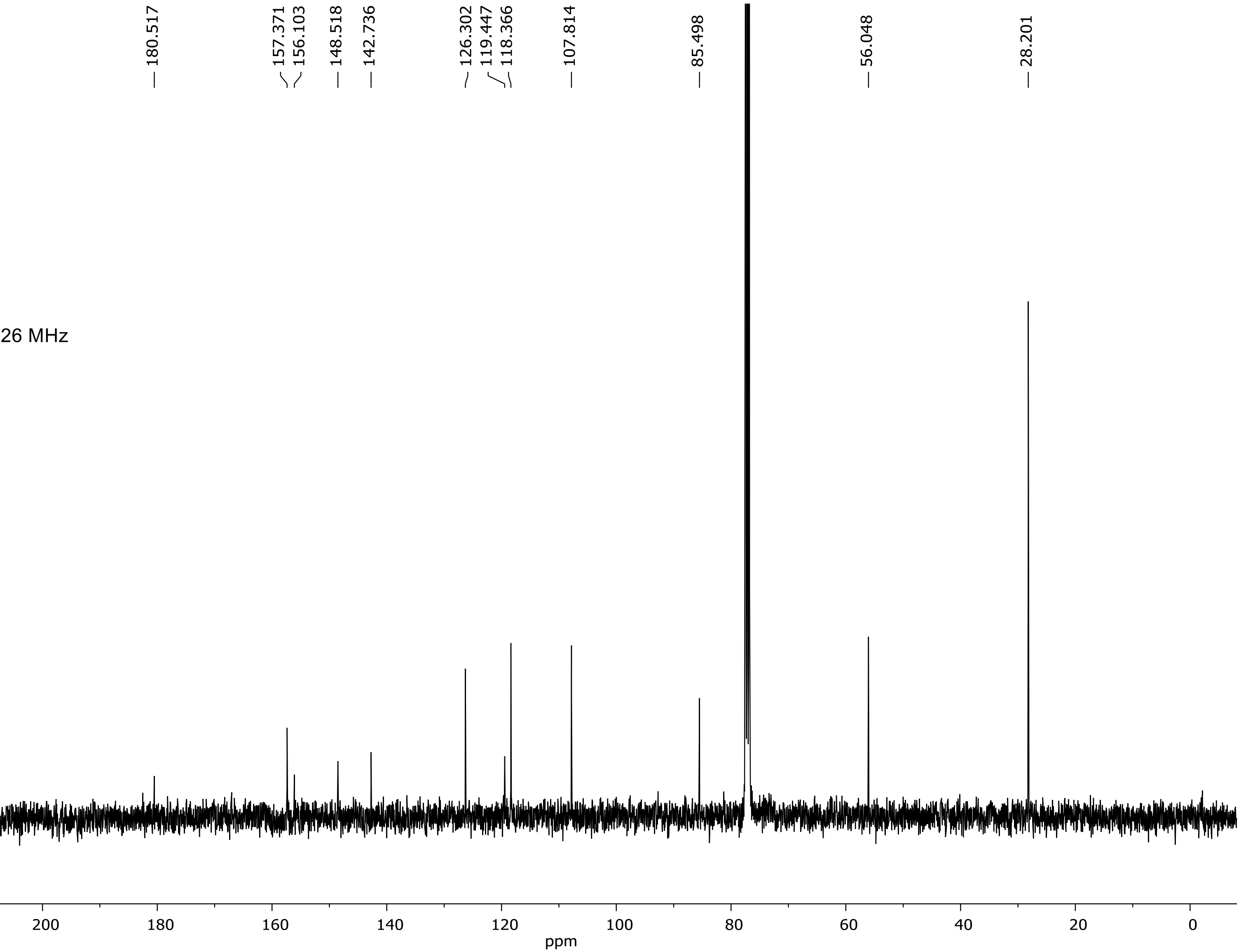

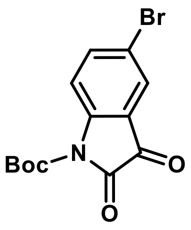

**S2**

$^1H$ NMR, $CDCl_3$, 400 MHz

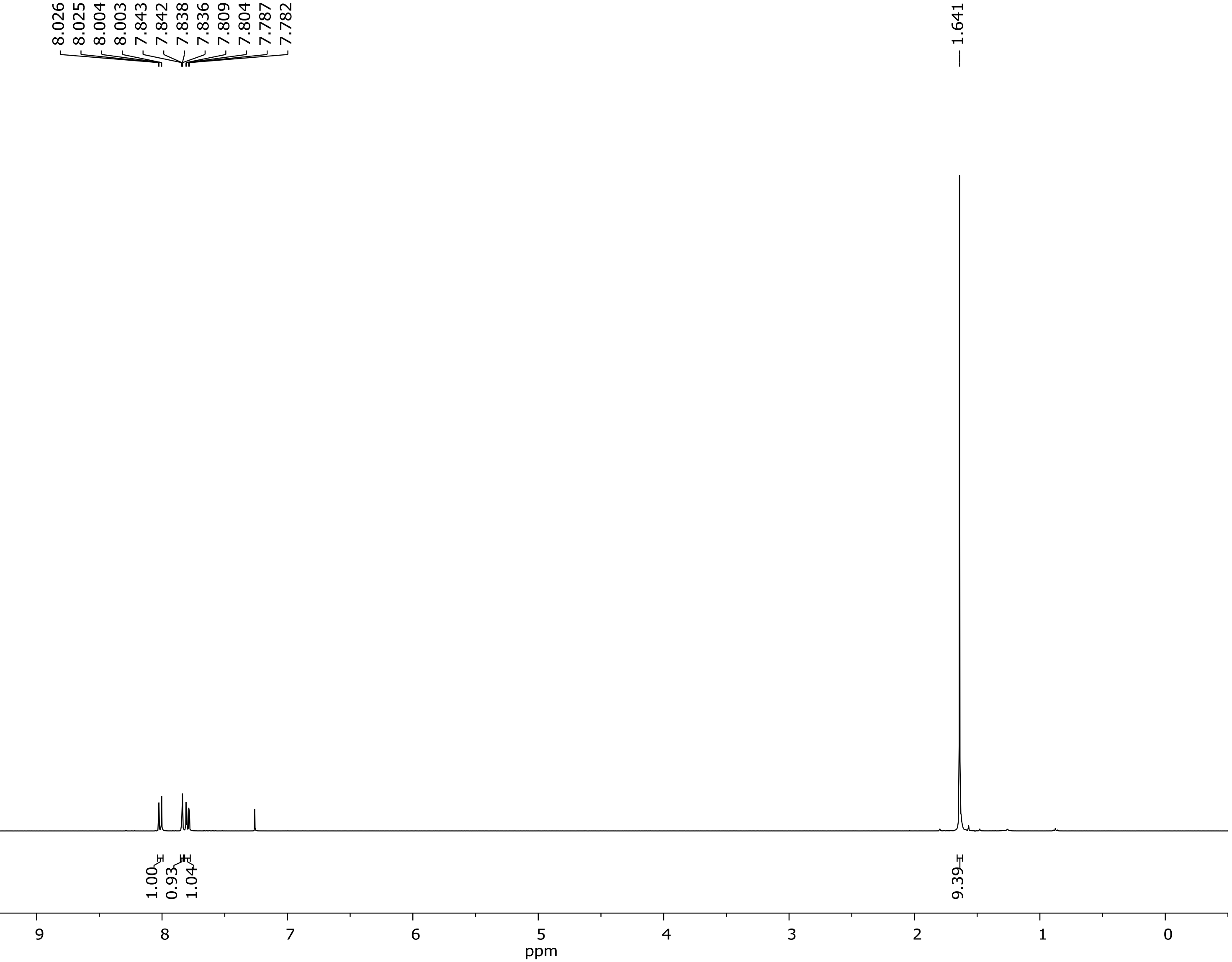

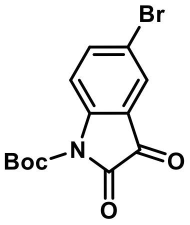


**S2**

$^{13}C\{^1H\}$ NMR, $CDCl_3$, 126 MHz

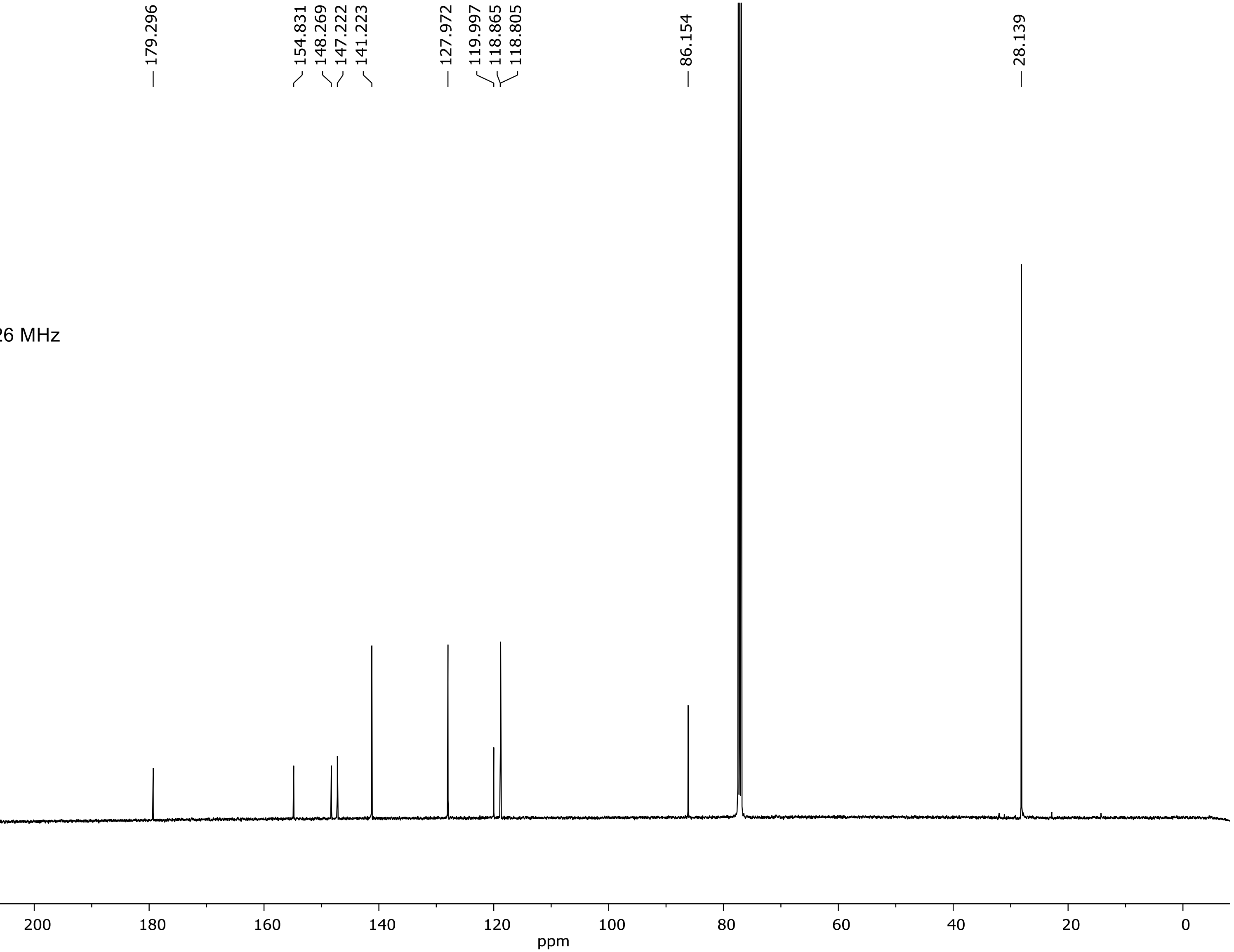

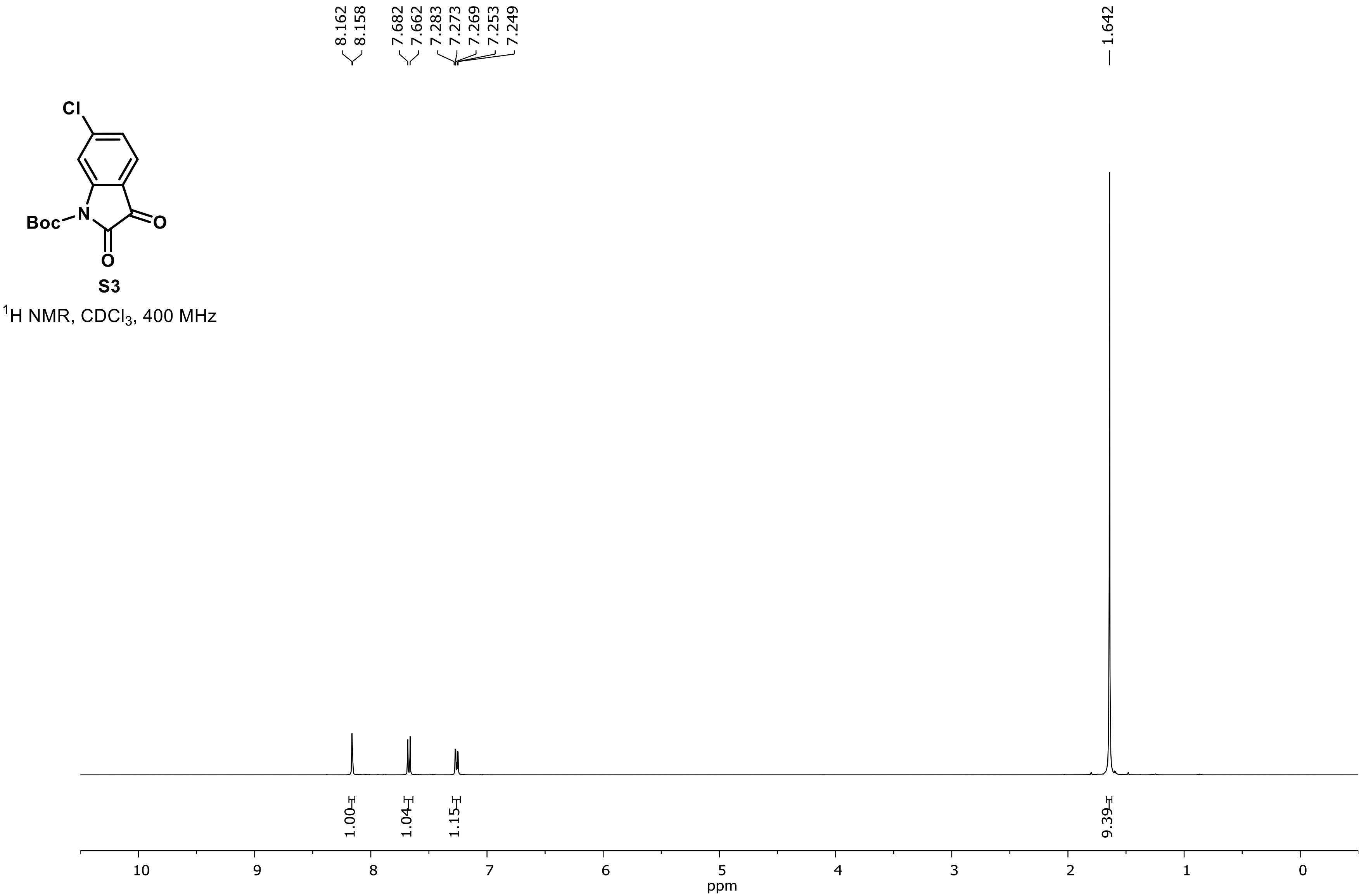


**S3**

$^1$H NMR, $CDCl_3$, 400 MHz

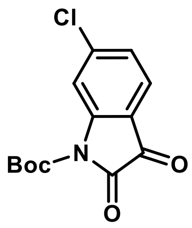

**S3**

$^{13}C\{^1H\}$ NMR, $CDCl_3$, 126 MHz

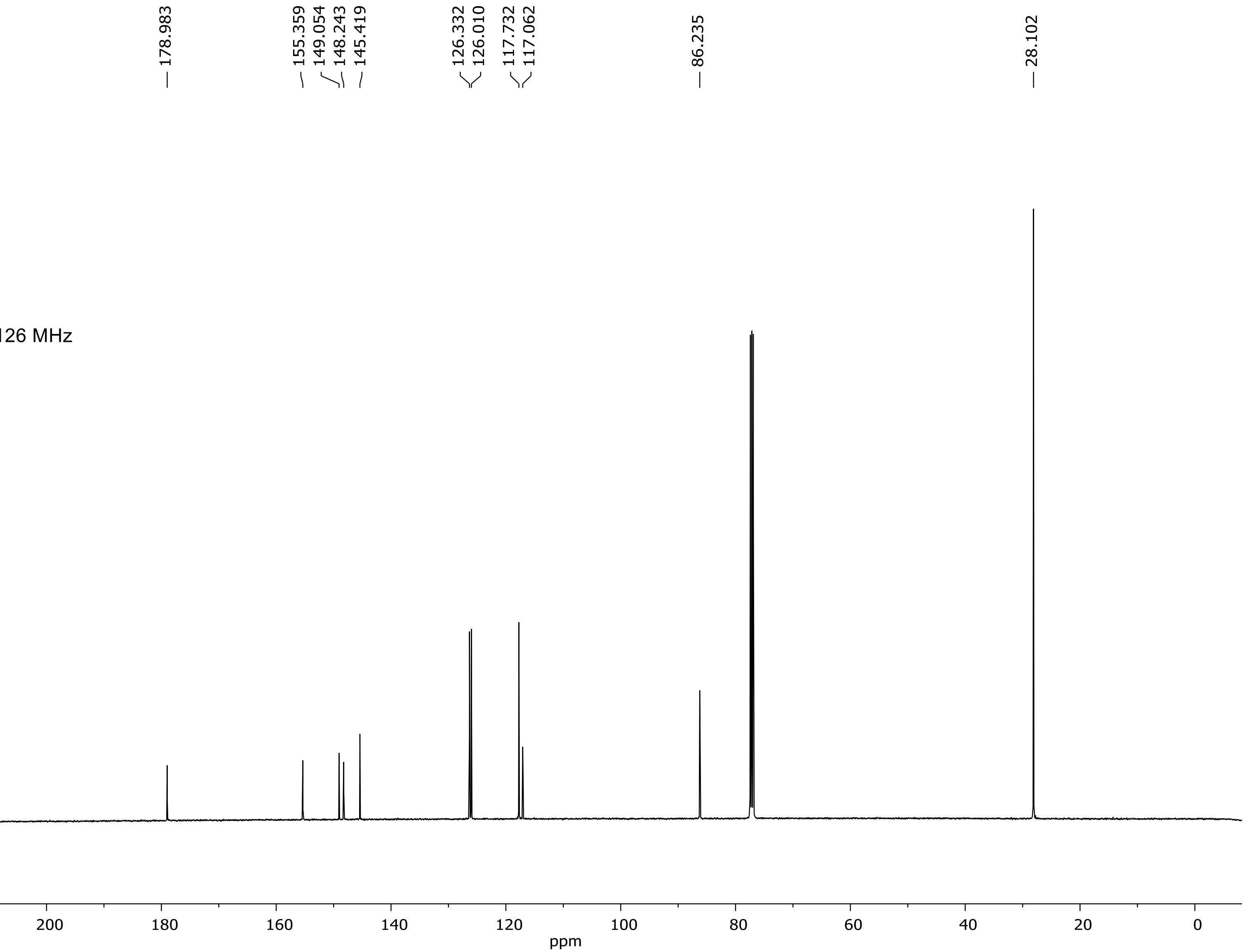

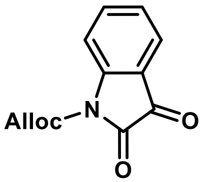


**S3**

$^{1}$H NMR, $CDCl_3$, 400 MHz

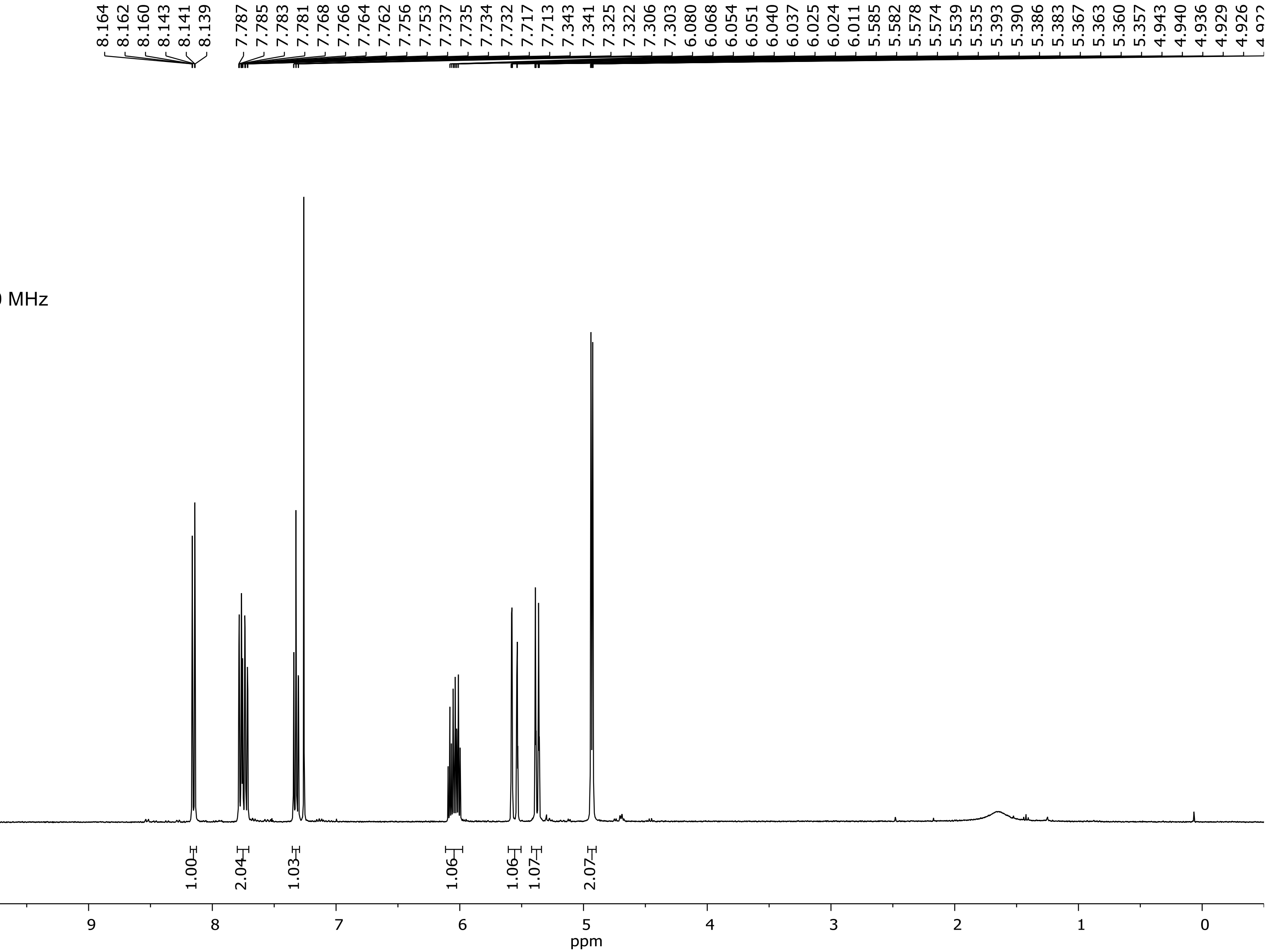

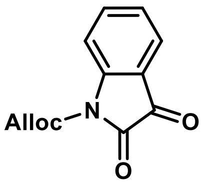


**S3**

$^{13}C\{^{1}H\}$ NMR, $CDCl_3$, 126 MHz

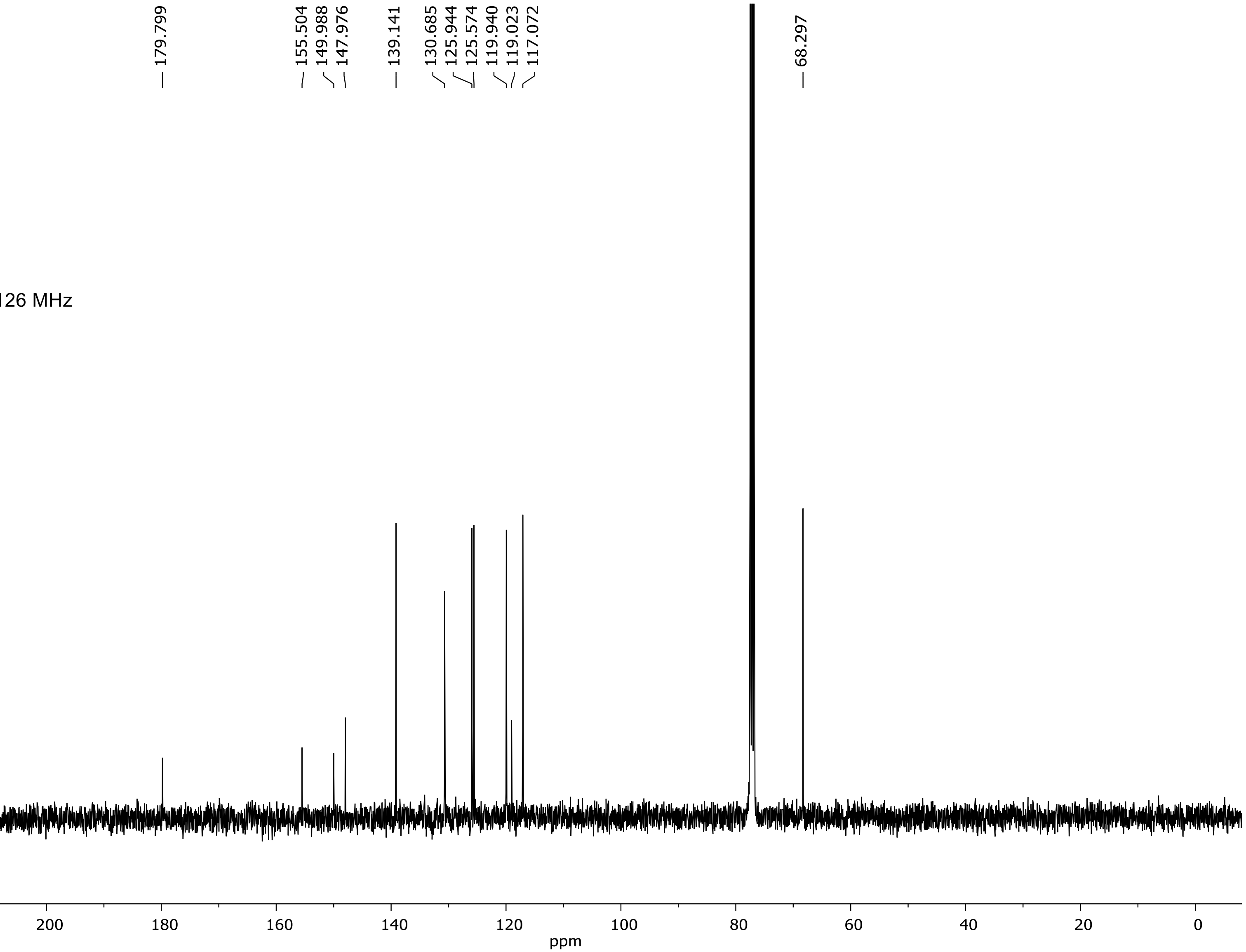

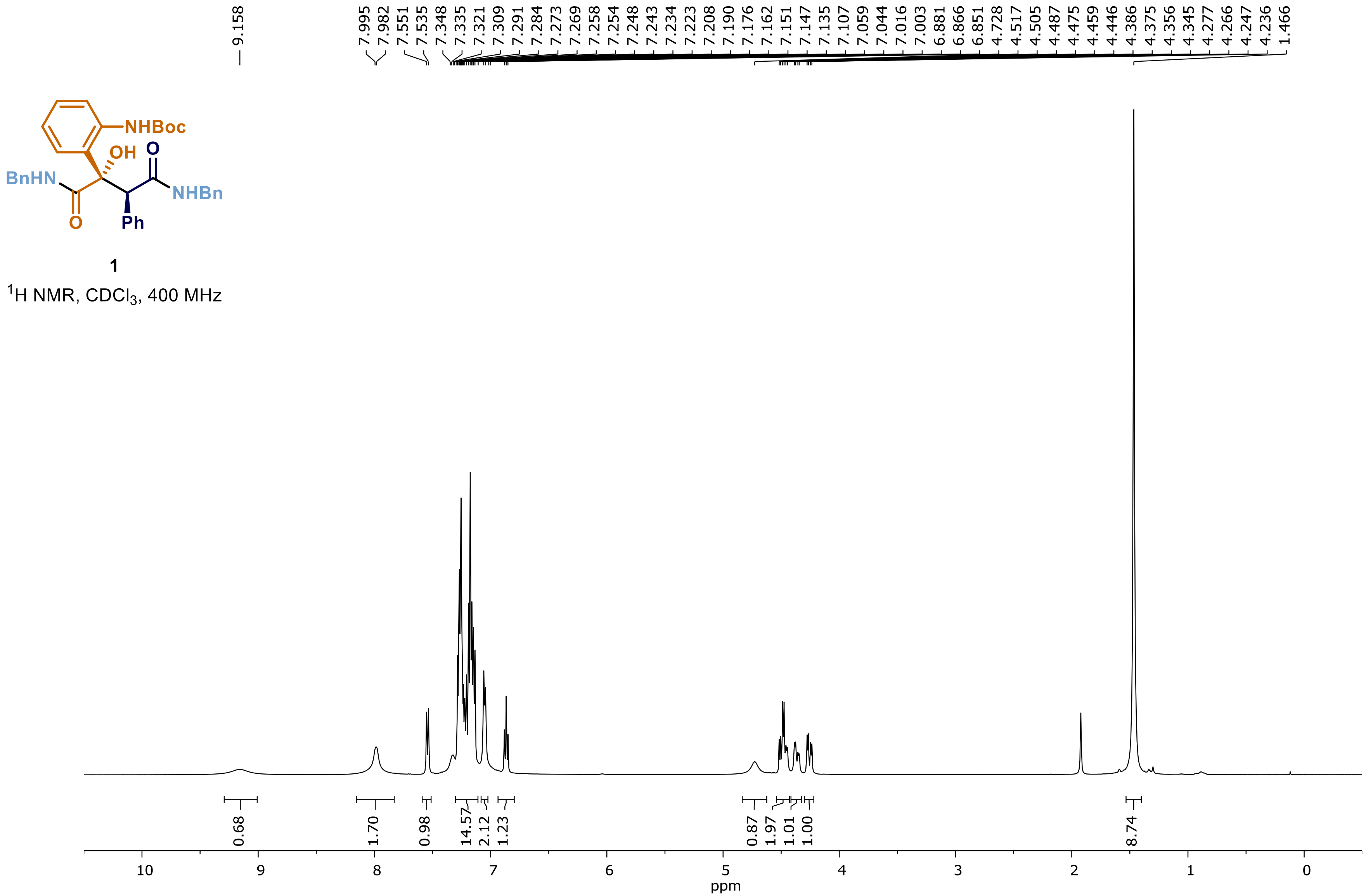


**1**

$^{1}$H NMR, $CDCl_3$, 400 MHz

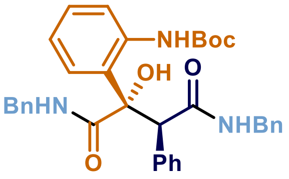


**1**

$^{13}C\{^1H\}$ NMR, $CDCl_3$, 101 MHz

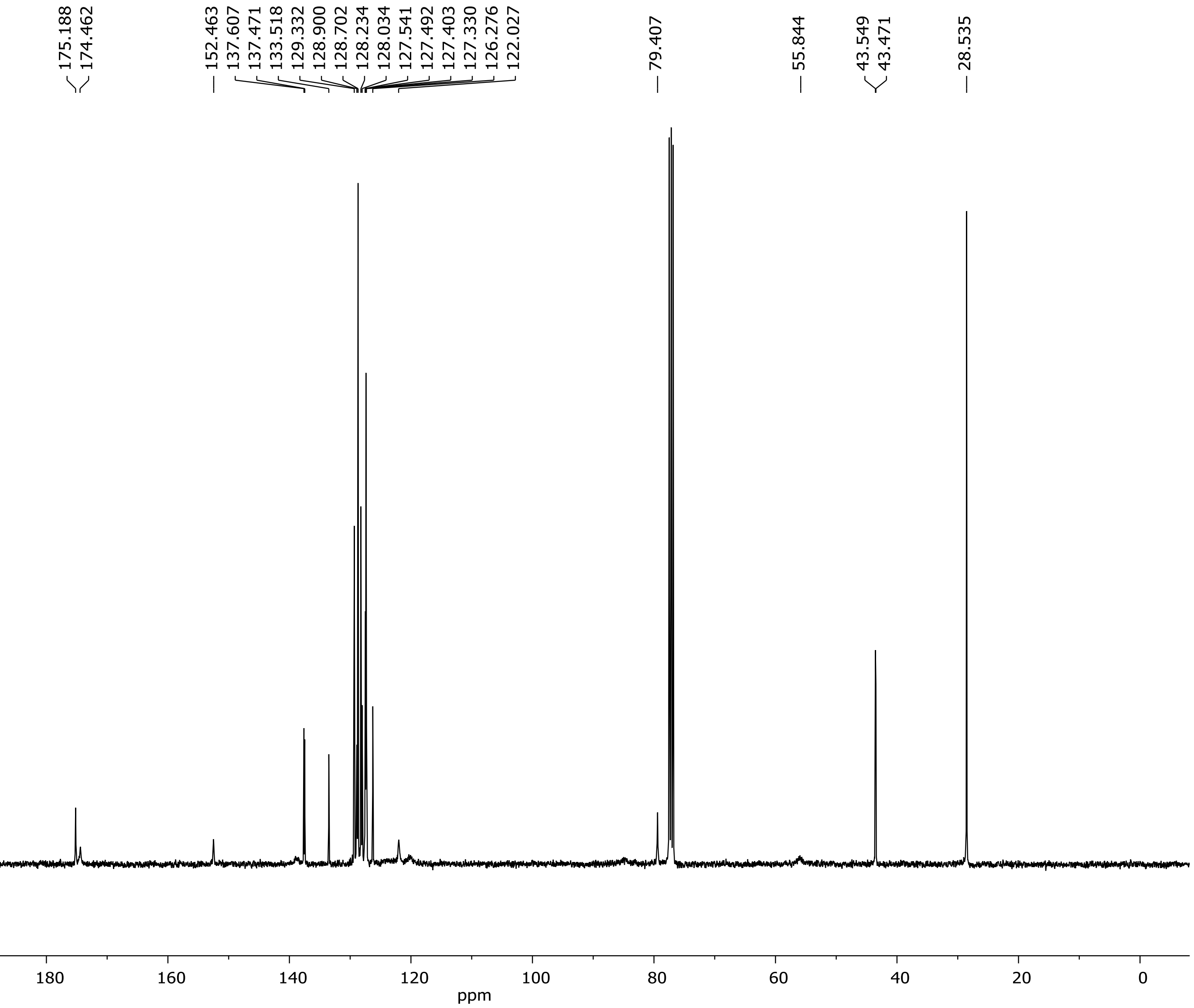

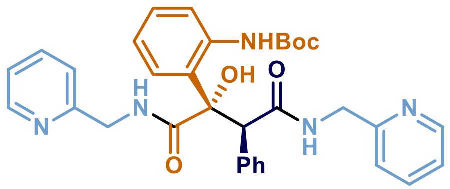


**2**

$^1$H NMR, $CDCl_3$, 500 MHz

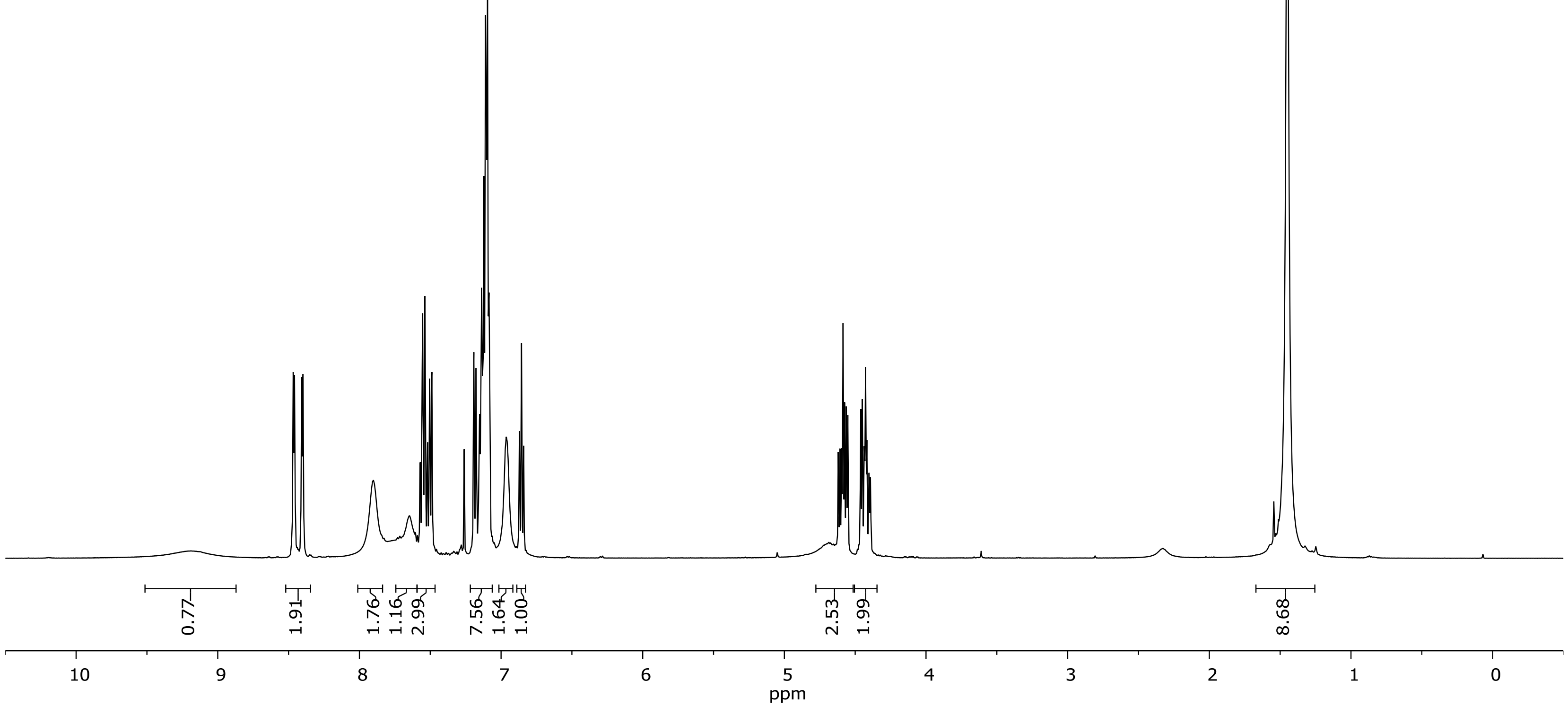

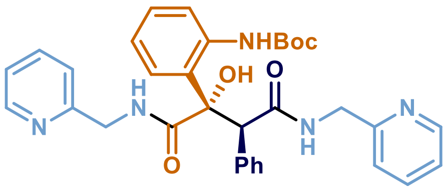

**2**

$^{13}C\{^{1}H\}$ NMR, $CDCl_3$, 126 MHz

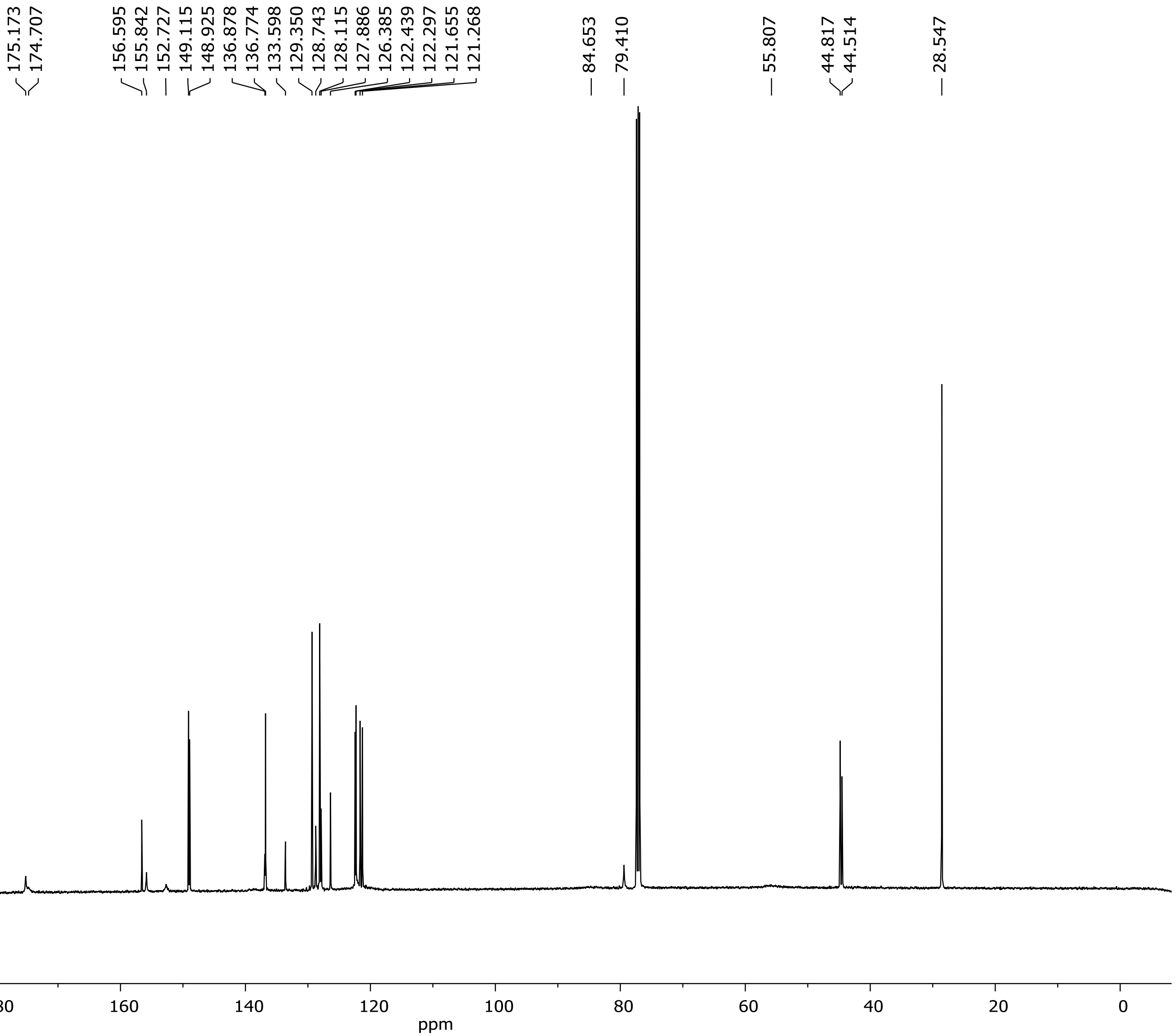

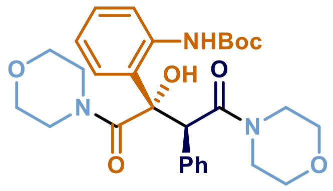


**3**

$^{1}H$ NMR, $CDCl_3$, 400 MHz

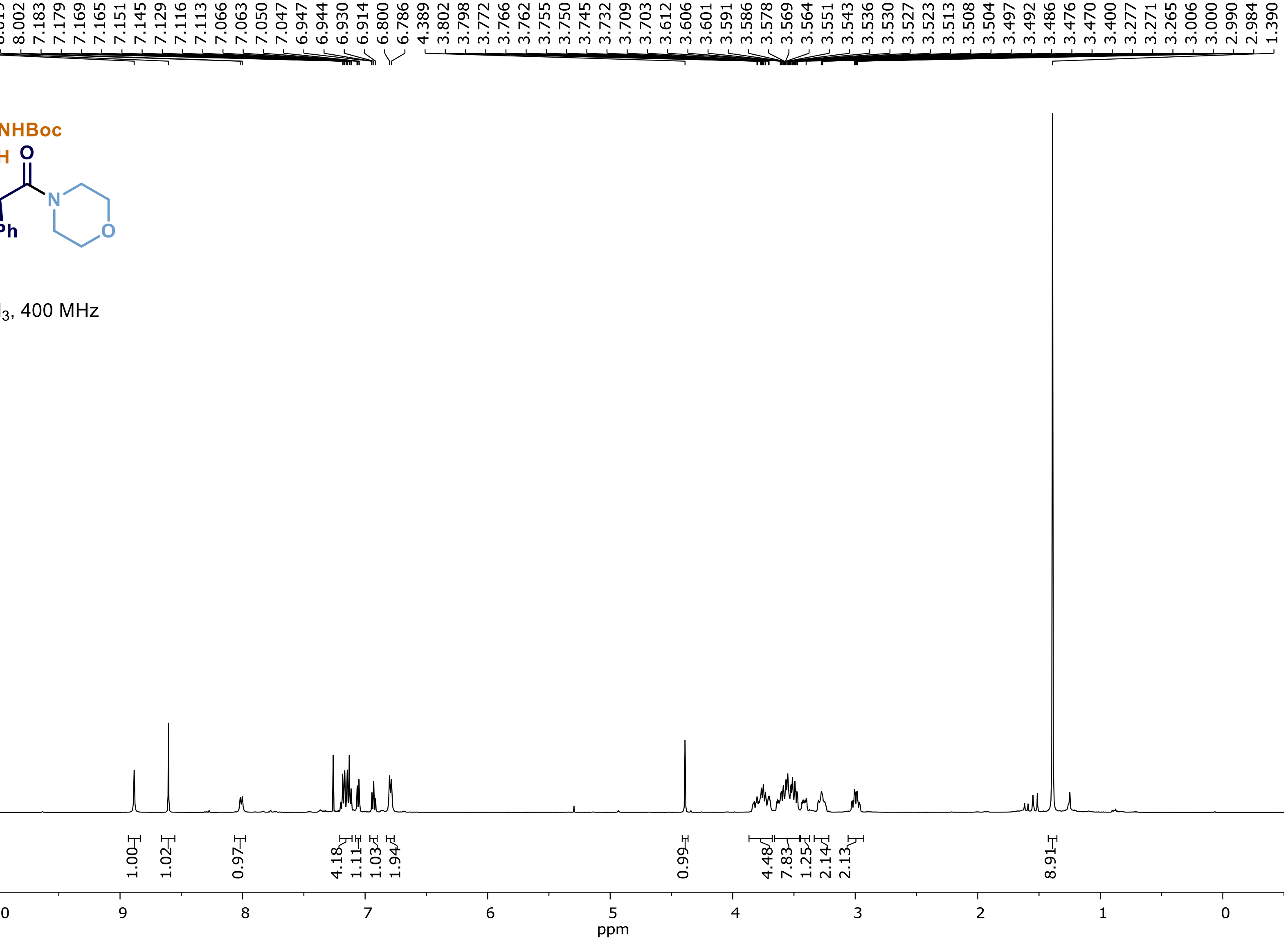

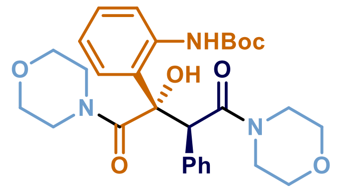


**3**

$^{13}C\{^{1}H\}$ NMR, $CDCl_3$, 101 MHz

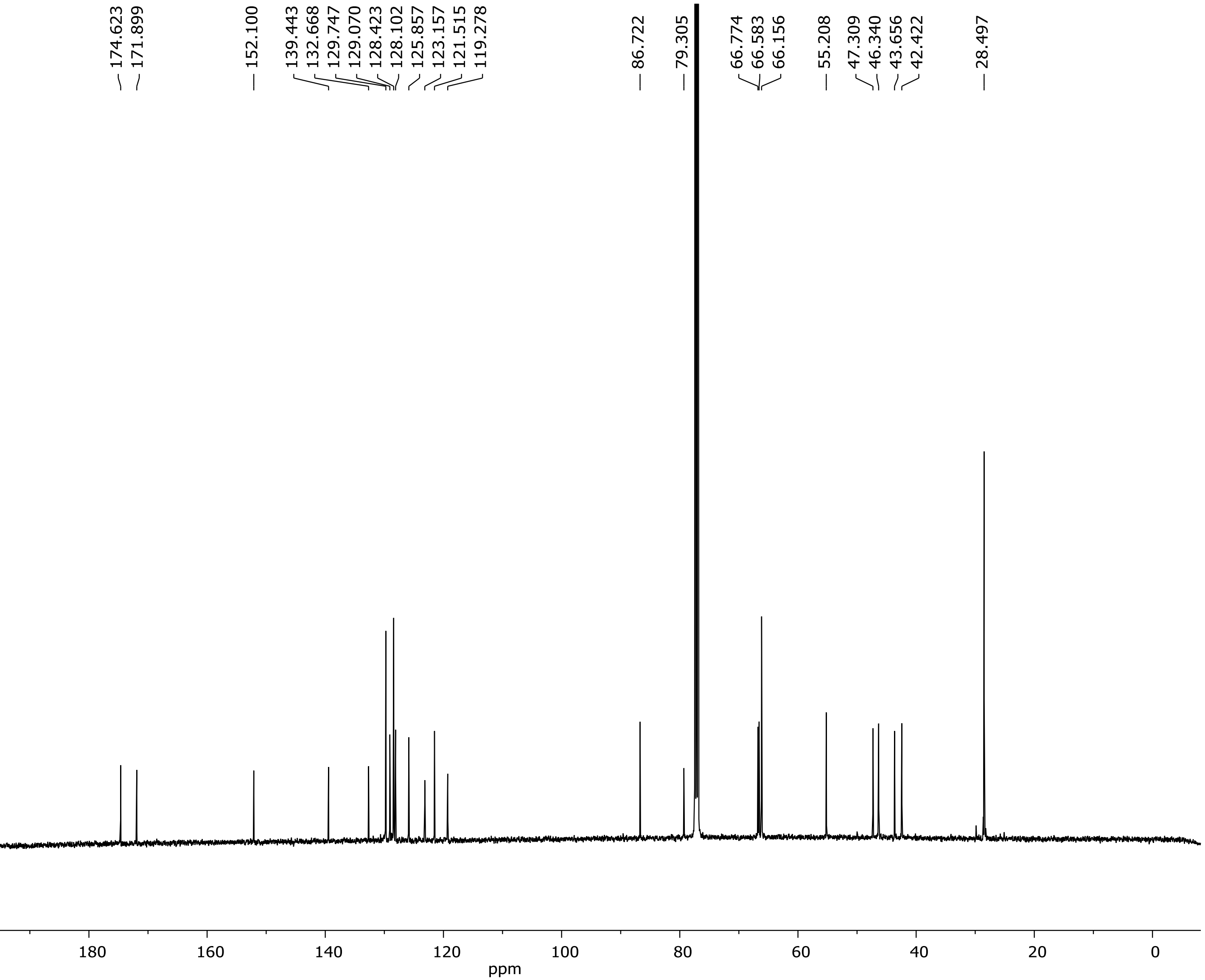

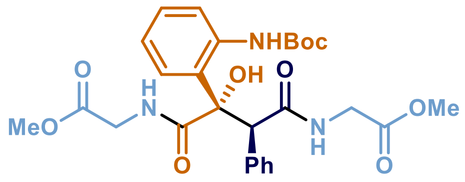


**4**

$^{1}H$ NMR, $CDCl_3$, 500 MHz

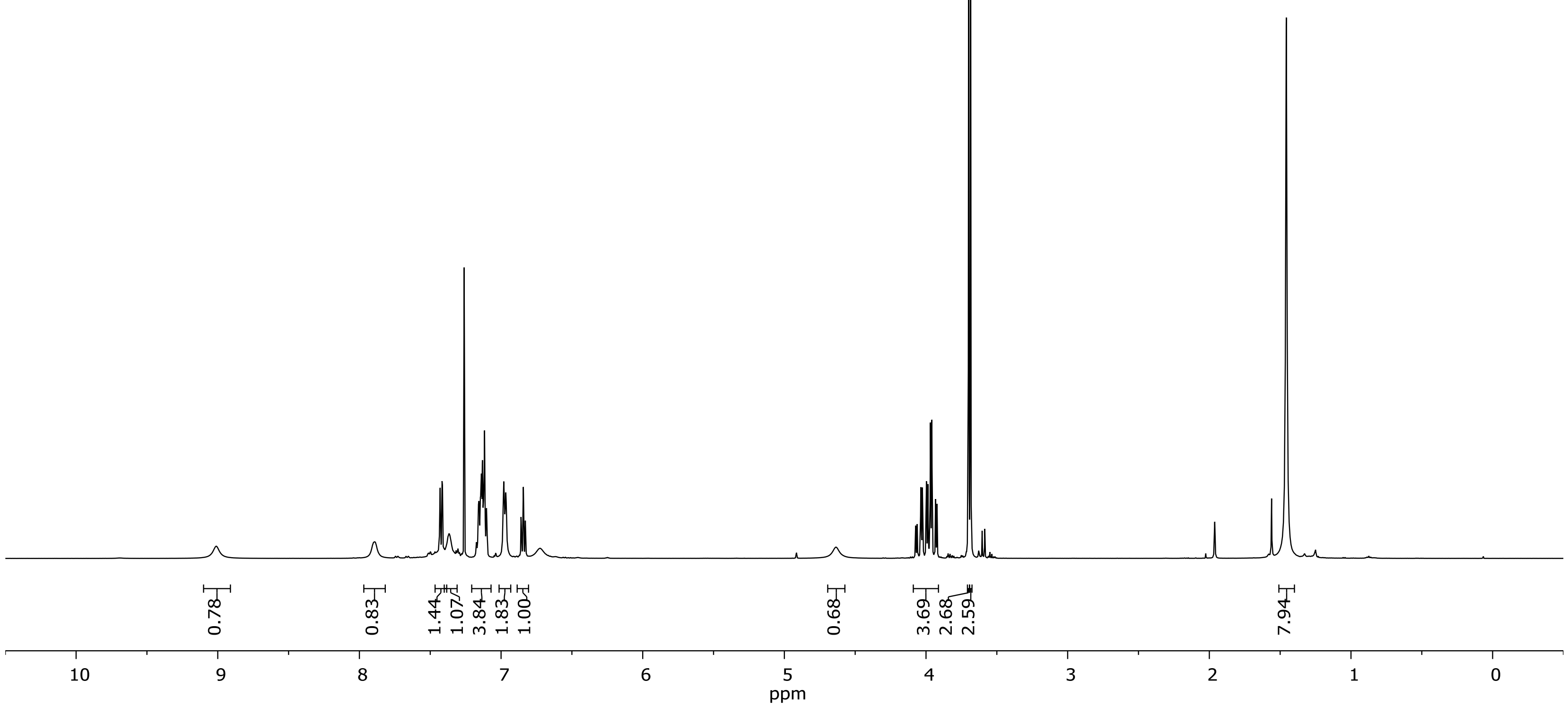

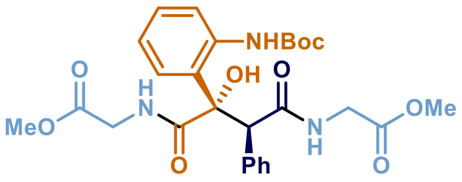


**4**

$^{13}C\{^{1}H\}$ NMR, $CDCl_3$, 126 MHz

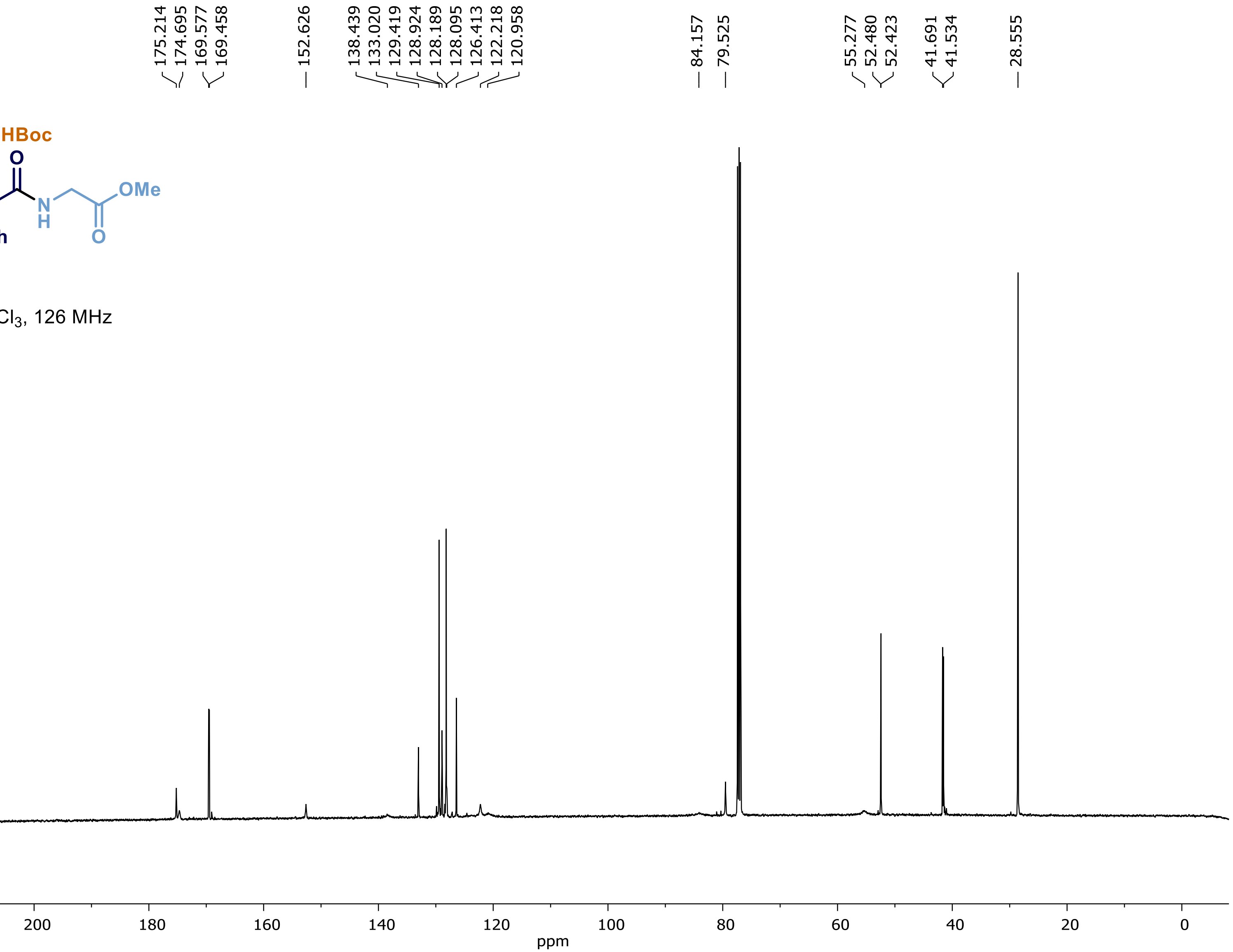

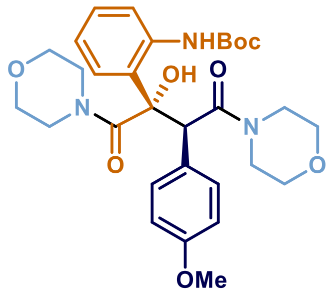

**5**

[1]H NMR, $CDCl_3$, 400 MHz

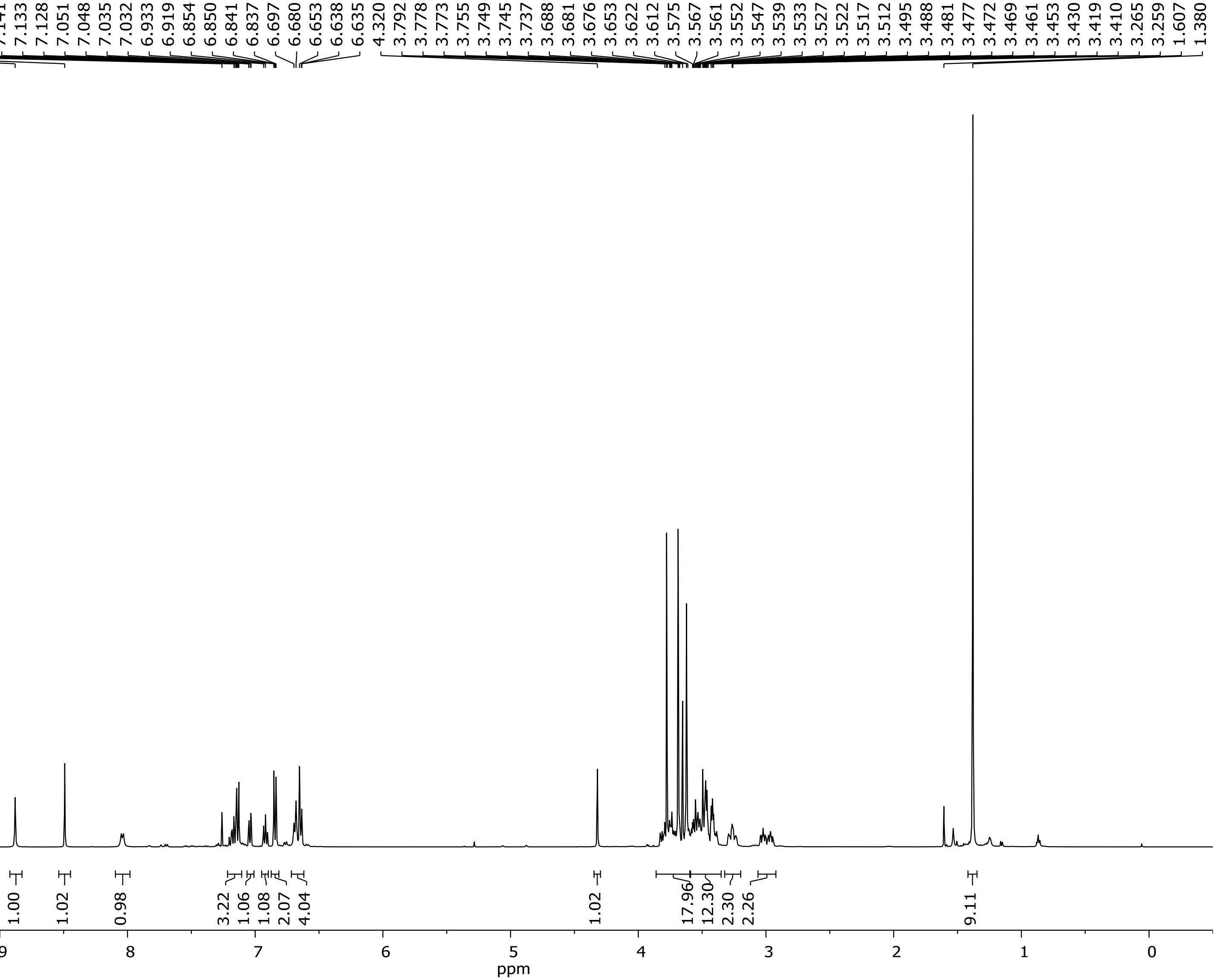

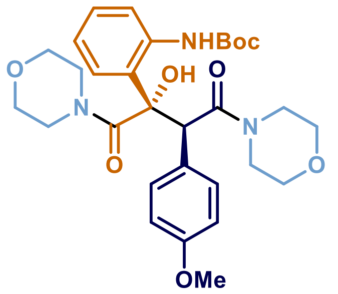


**5**

$^{13}C\{^{1}H\}$ NMR, $CDCl_3$, 101 MHz

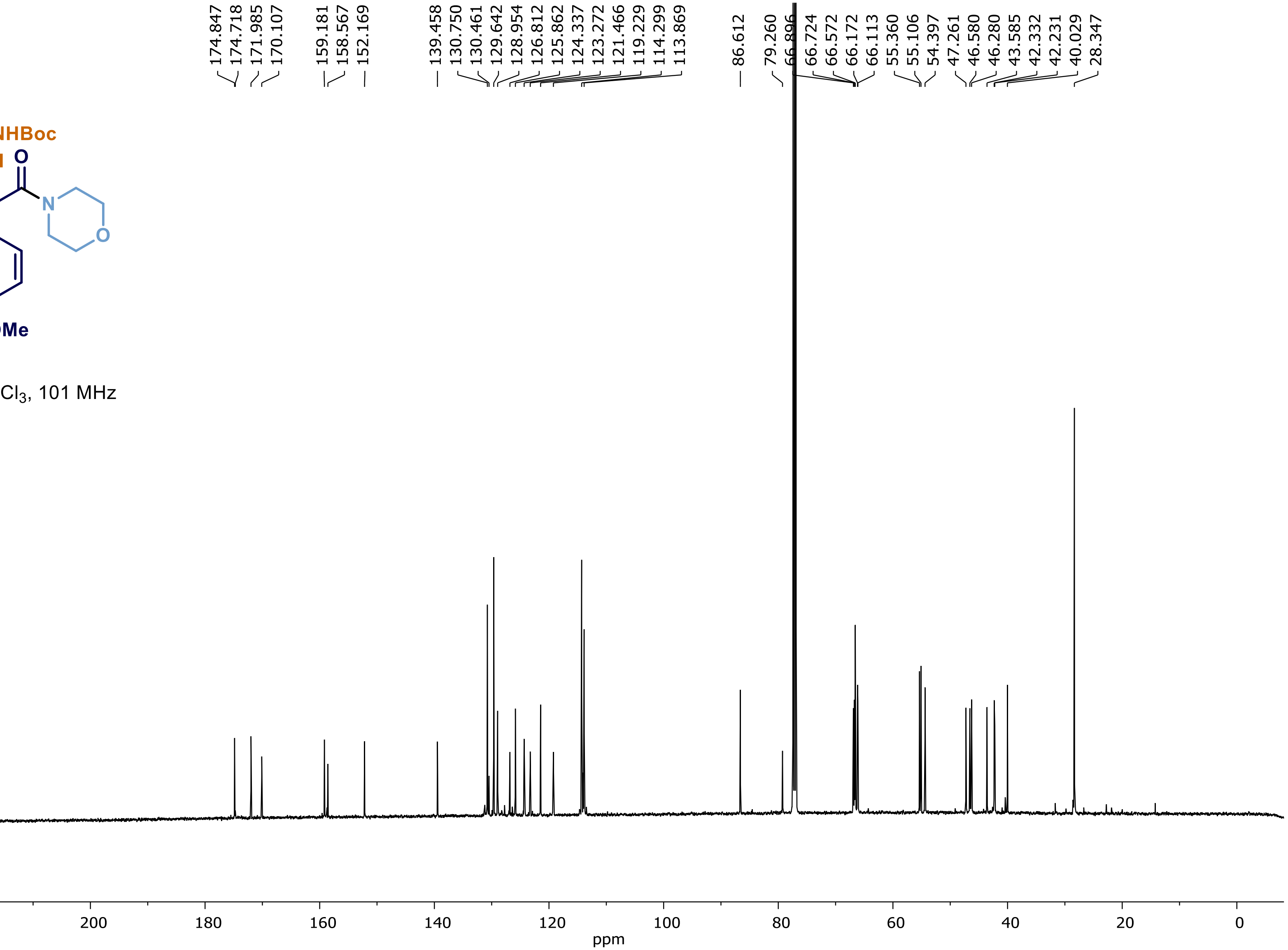

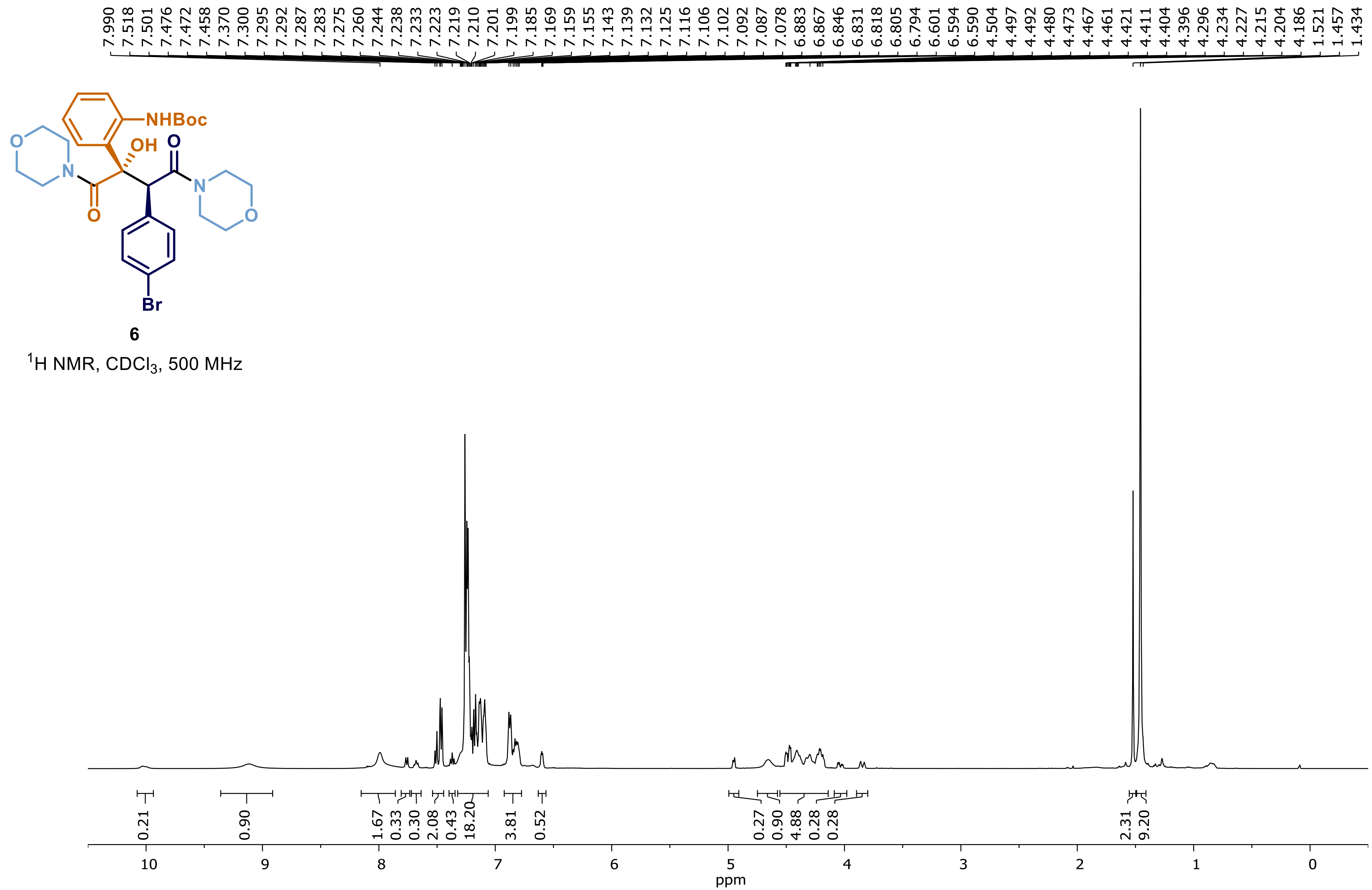


**6**

$^1$H NMR, $CDCl_3$, 500 MHz

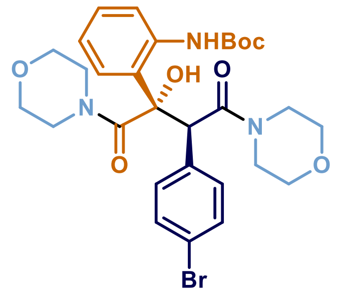

**6**

$^{13}C\{^{1}H\}$ NMR, $CDCl_3$, 126 MHz

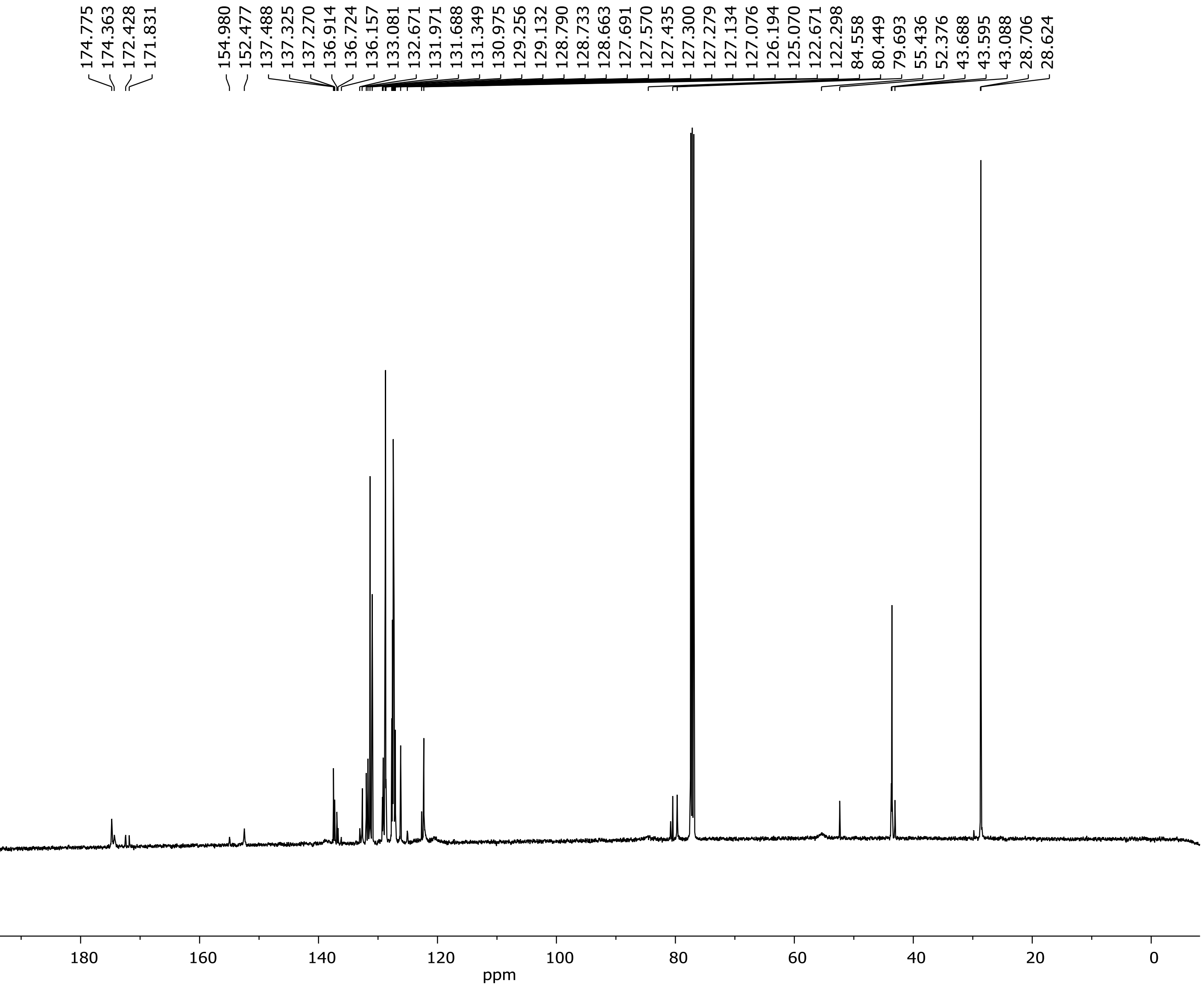

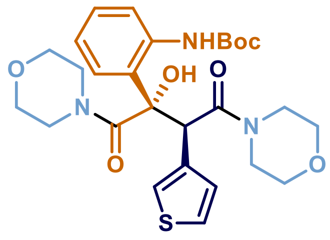

7

$^1H$ NMR, $CDCl_3$, 400 MHz

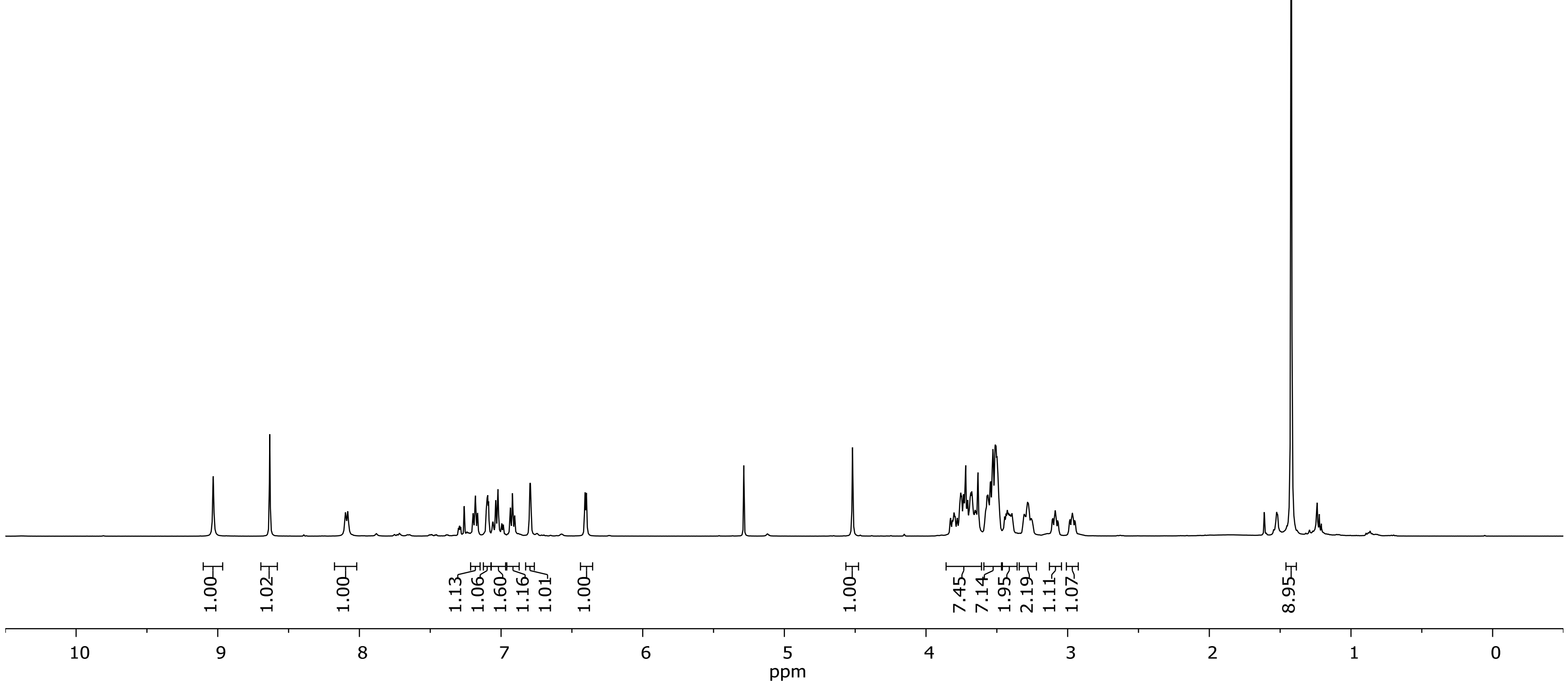

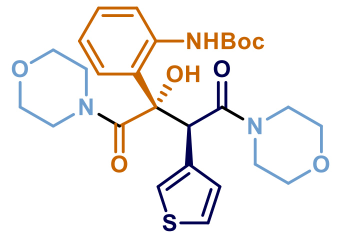


**7**

$^{13}C\{^1H\}$ NMR, $CDCl_3$, 101 MHz

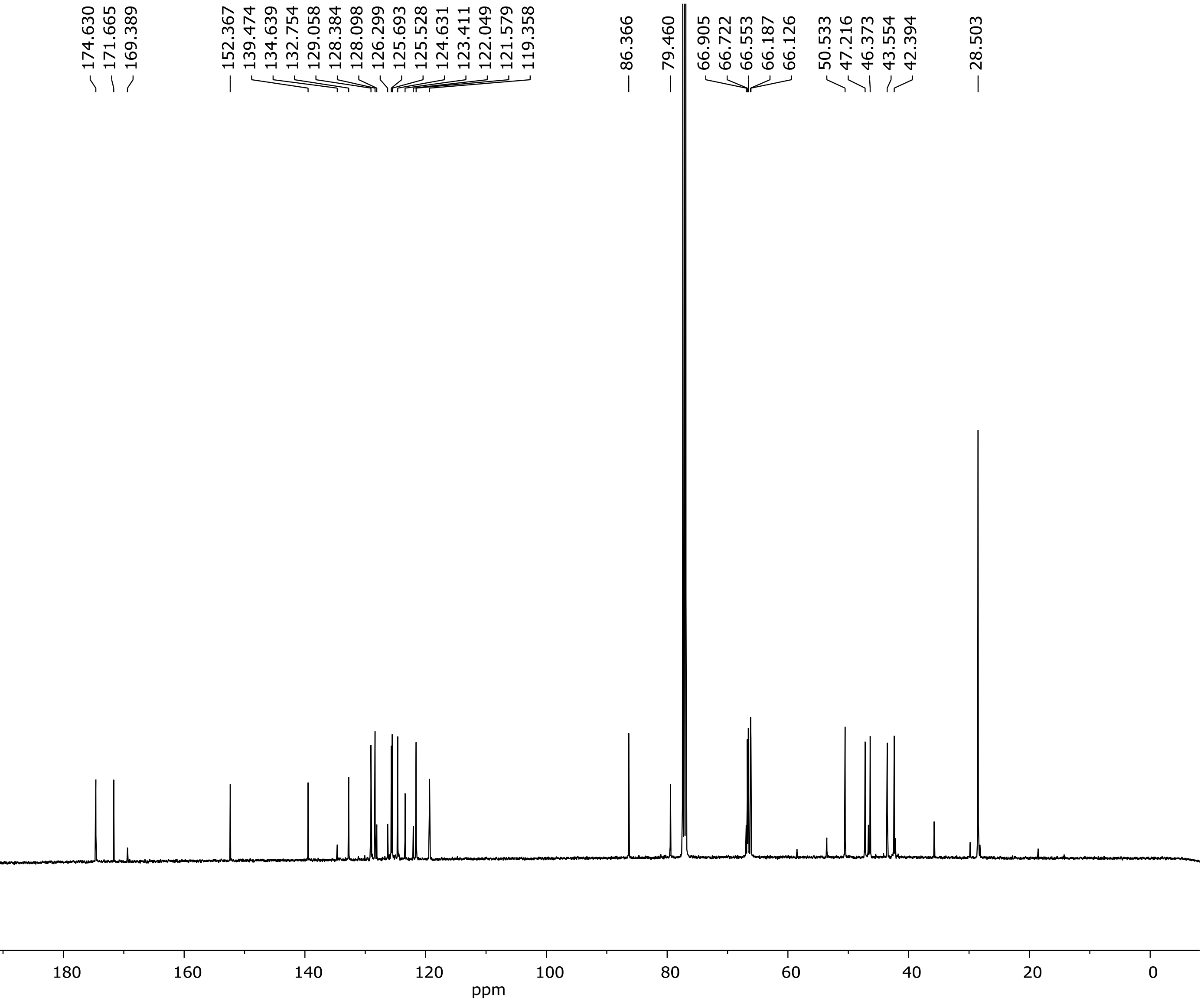

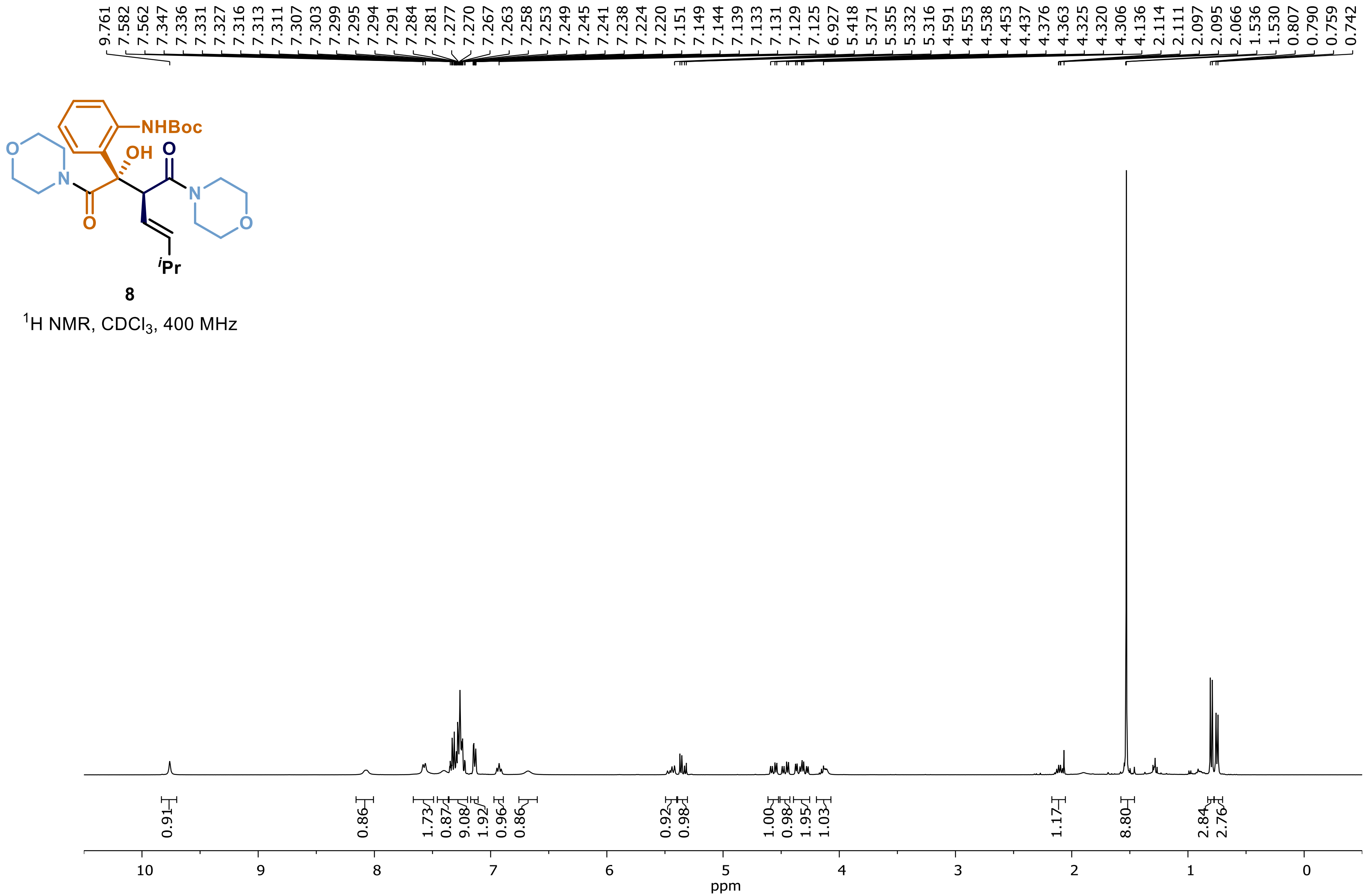


**8**

$^1$H NMR, $CDCl_3$, 400 MHz

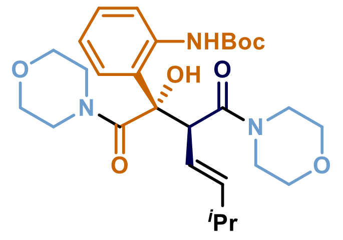

8

$^{13}C\{^1H\}$ NMR, $CDCl_3$, 126 MHz

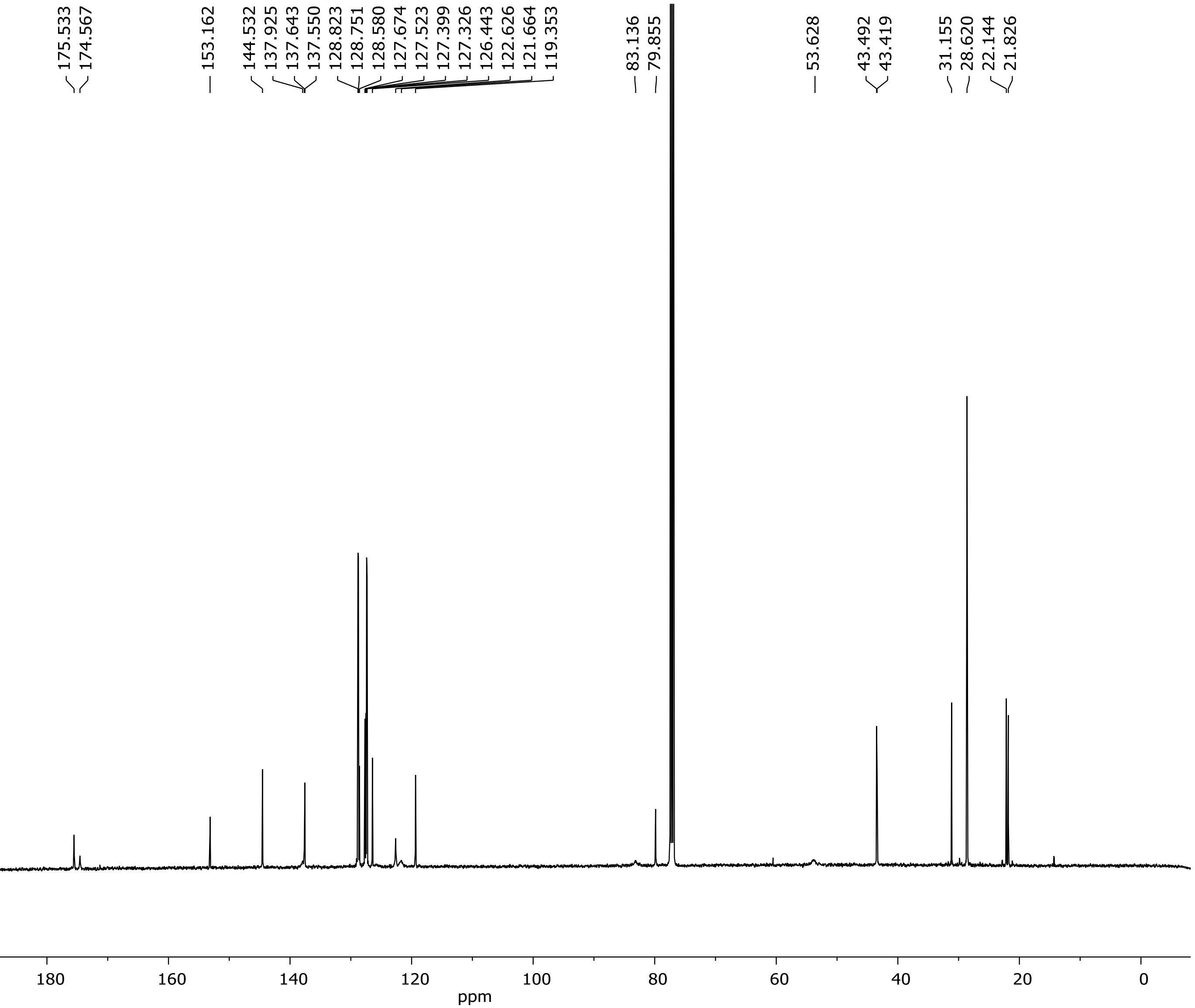

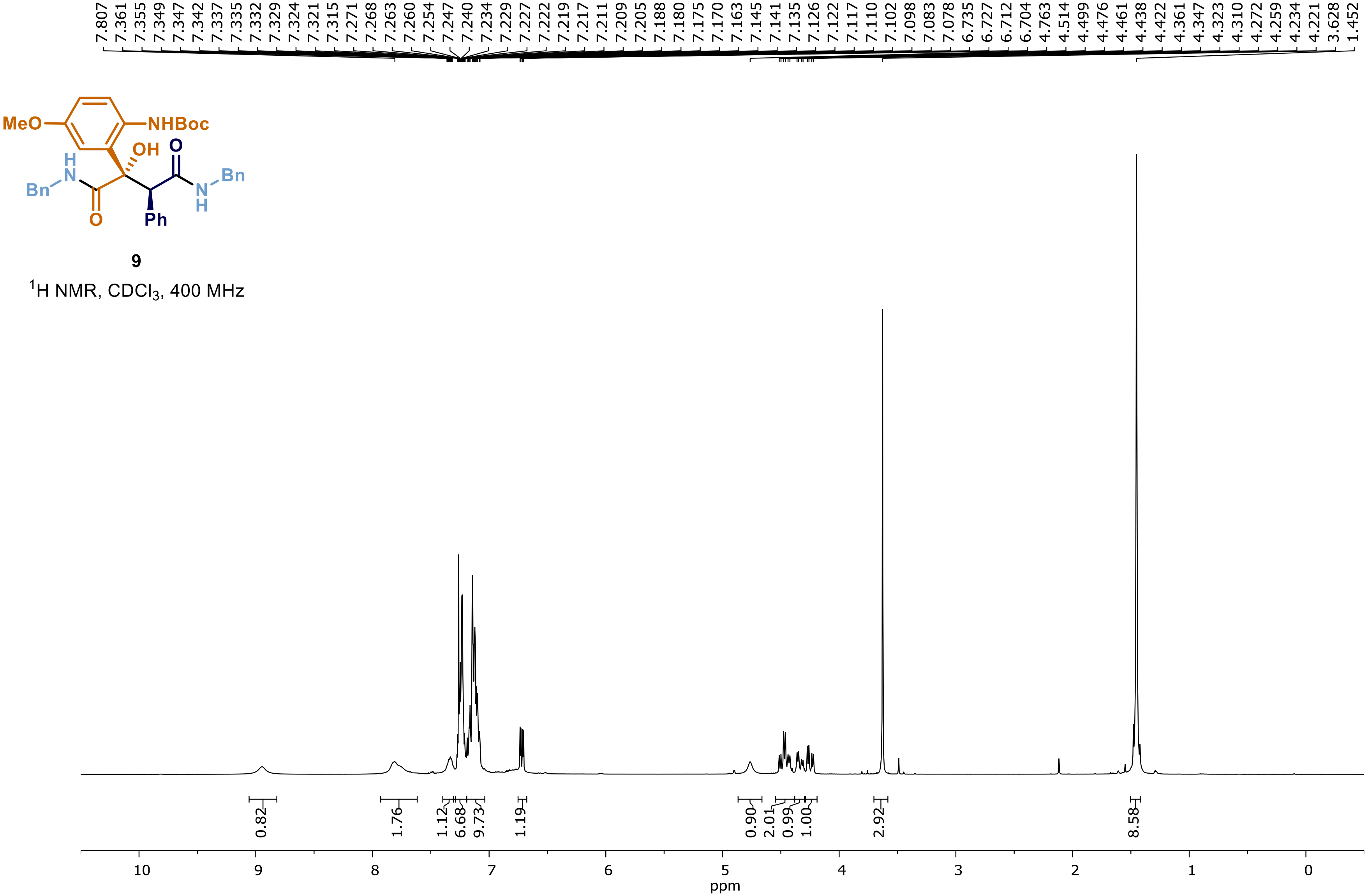


**9**

$^{1}H$ NMR, $CDCl_3$, 400 MHz

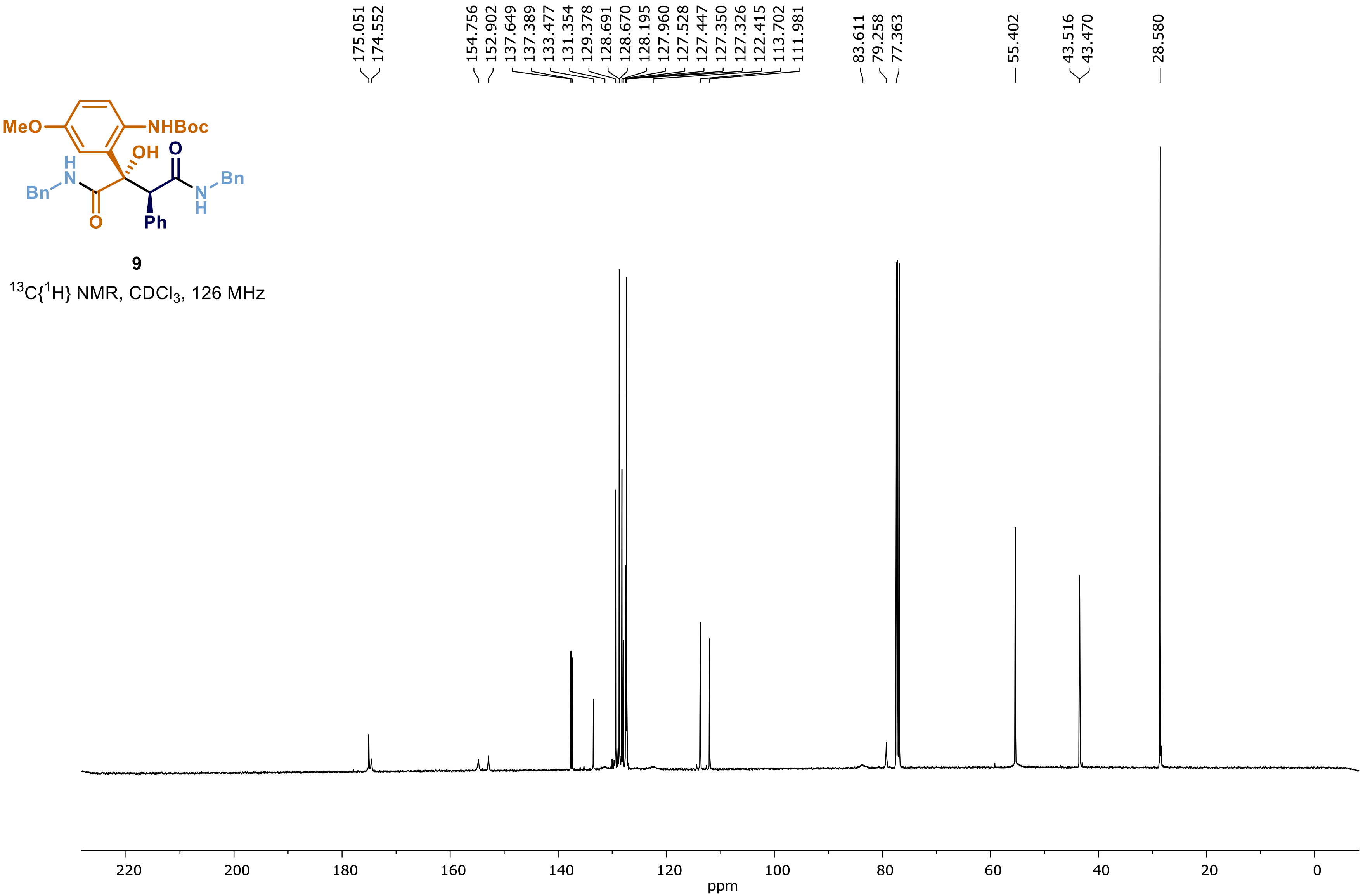
MeO
NHBoc
OH
O
H
N
Bn
Bn
N
H
O
Ph
9
$^{13}C\{^1H\}$ NMR, $CDCl_3$, 126 MHz
175.051
174.552
154.756
152.902
137.649
137.389
133.477
131.354
129.378
128.691
128.670
128.195
127.960
127.528
127.447
127.350
127.326
122.415
113.702
111.981
83.611
79.258
77.363
55.402
43.516
43.470
28.580
220
200
180
160
140
120
100
80
60
40
20
0
ppm

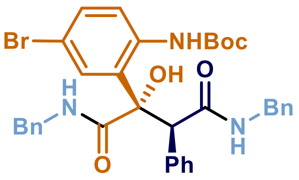


**10**

$^{1}$H NMR, $CDCl_3$, 500 MHz

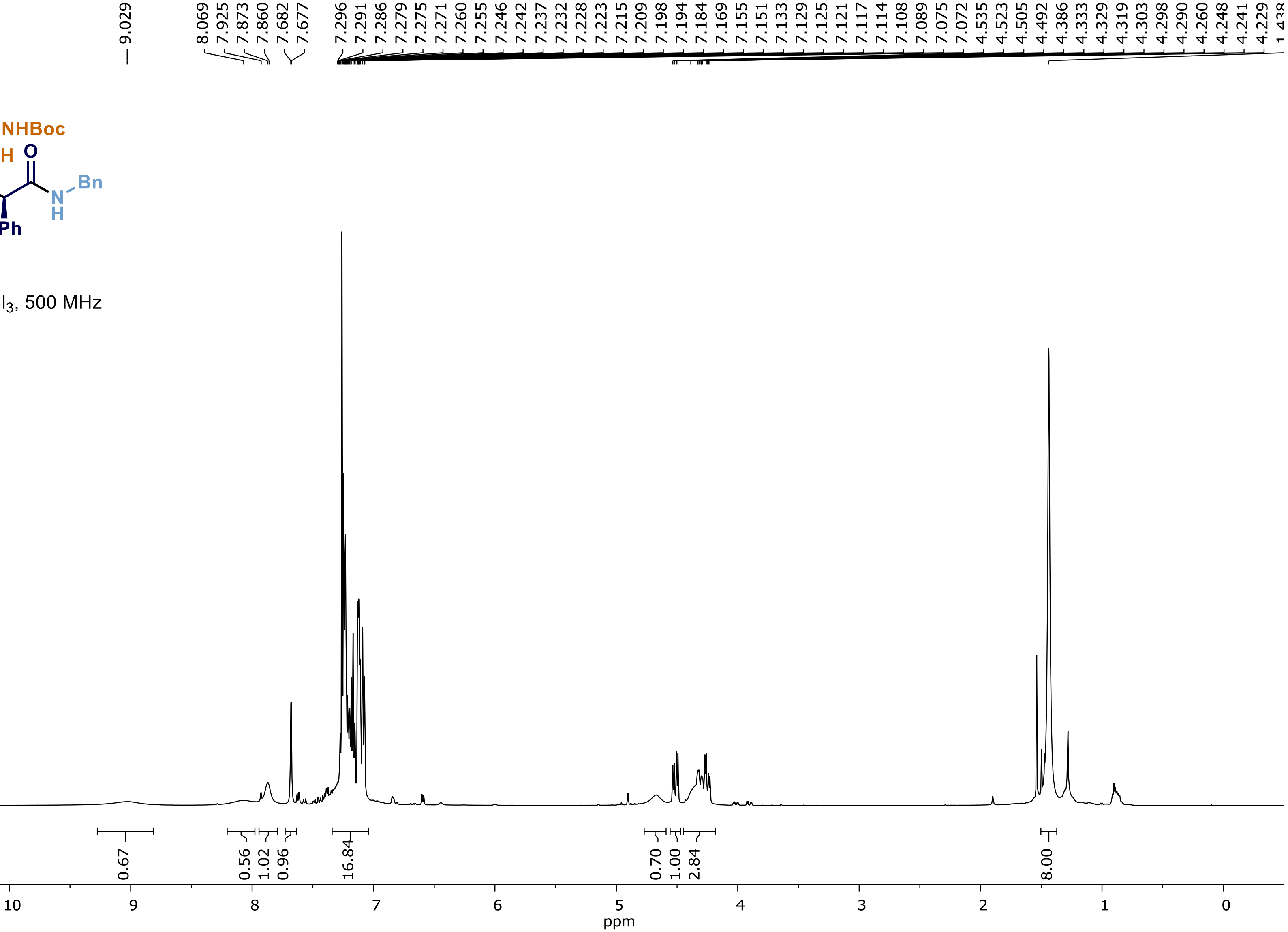

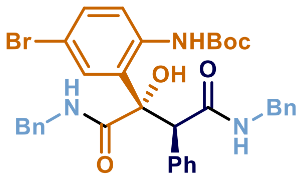


**10**

$^{13}C\{^{1}H\}$ NMR, $CDCl_3$, 126 MHz

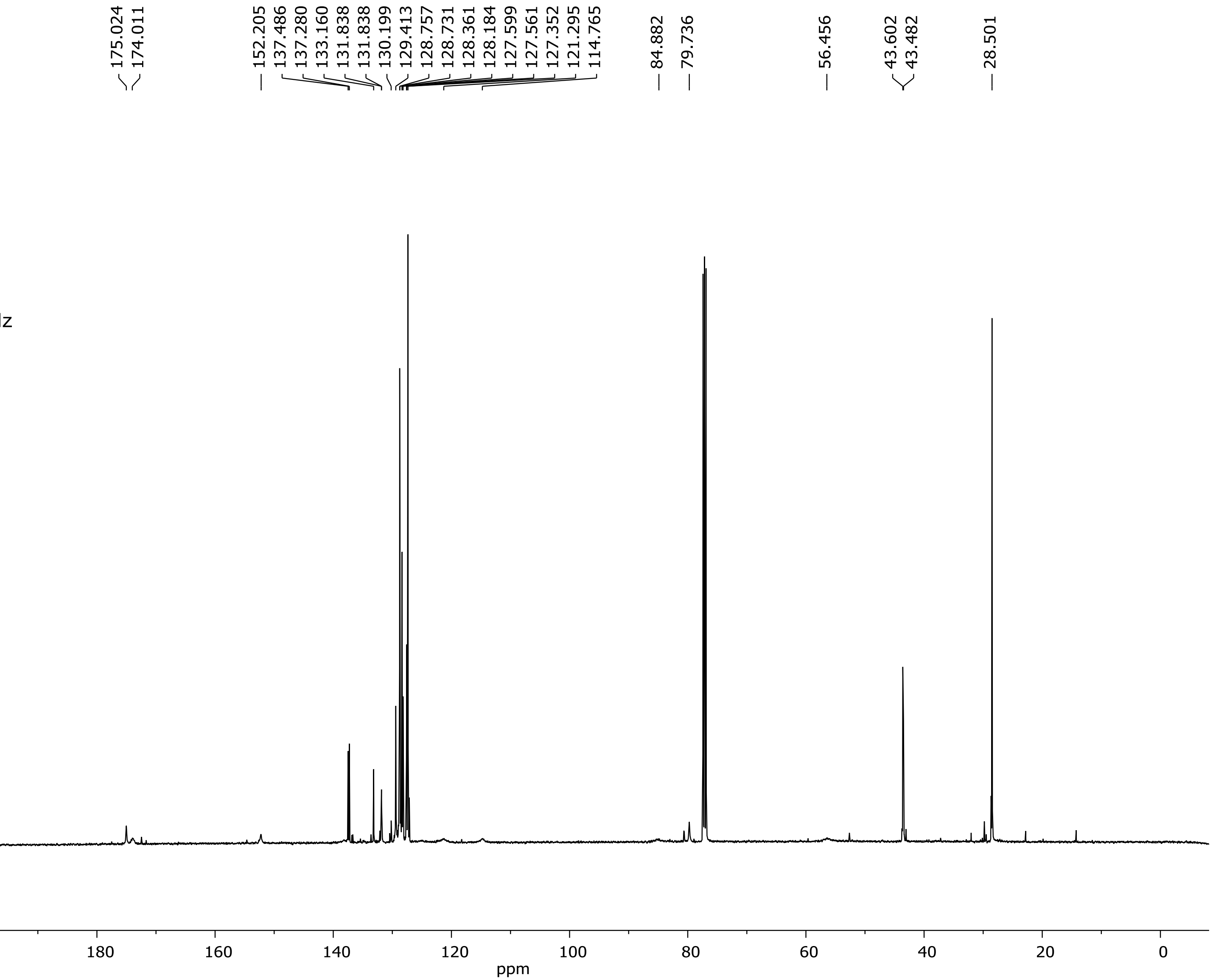

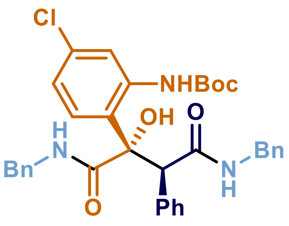


**11**

$^{1}$H NMR, $CDCl_3$, 500 MHz

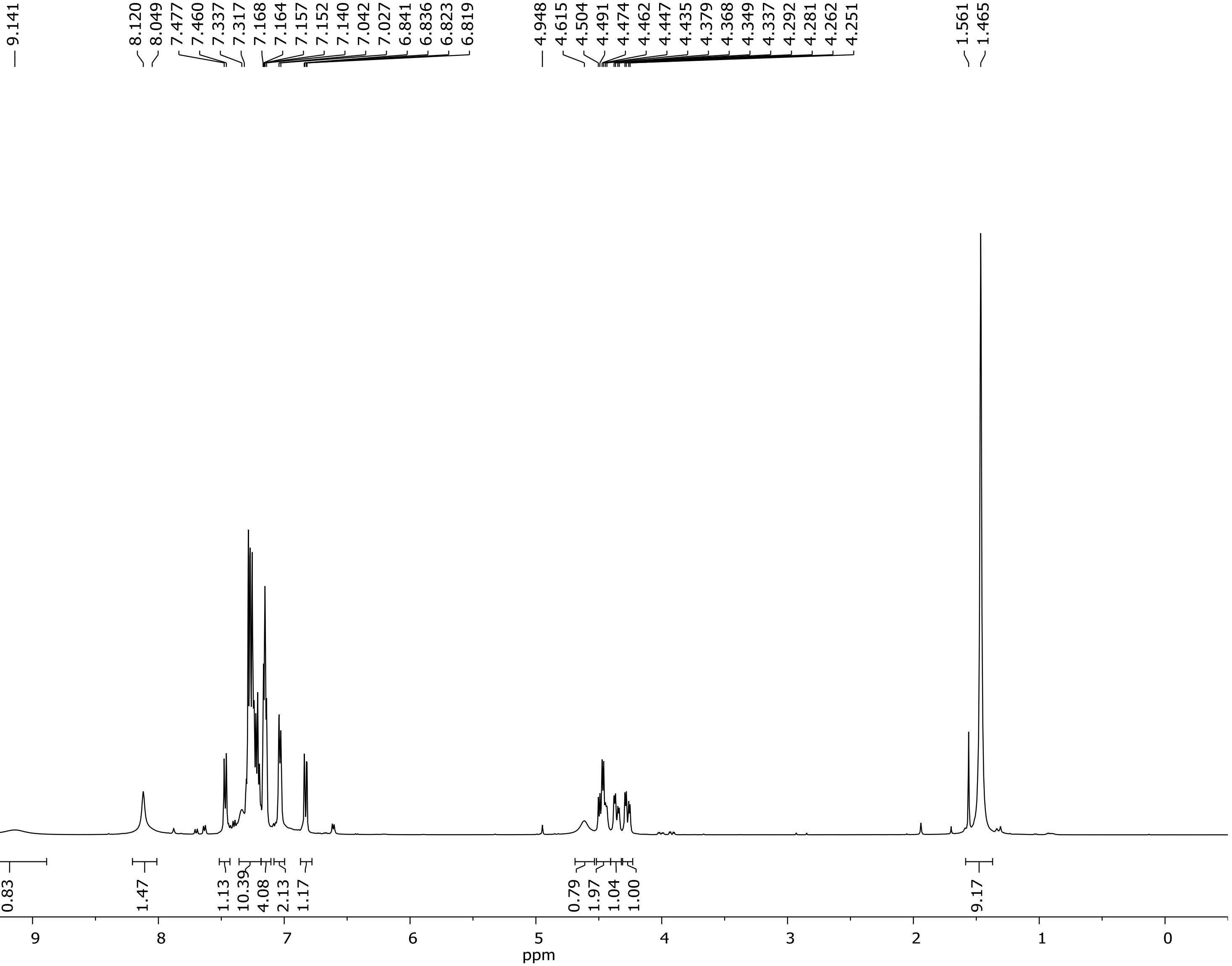

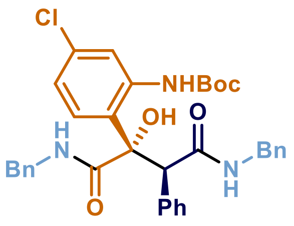


**11**

$^{13}C\{^{1}H\}$ NMR, $CDCl_3$, 126 MHz

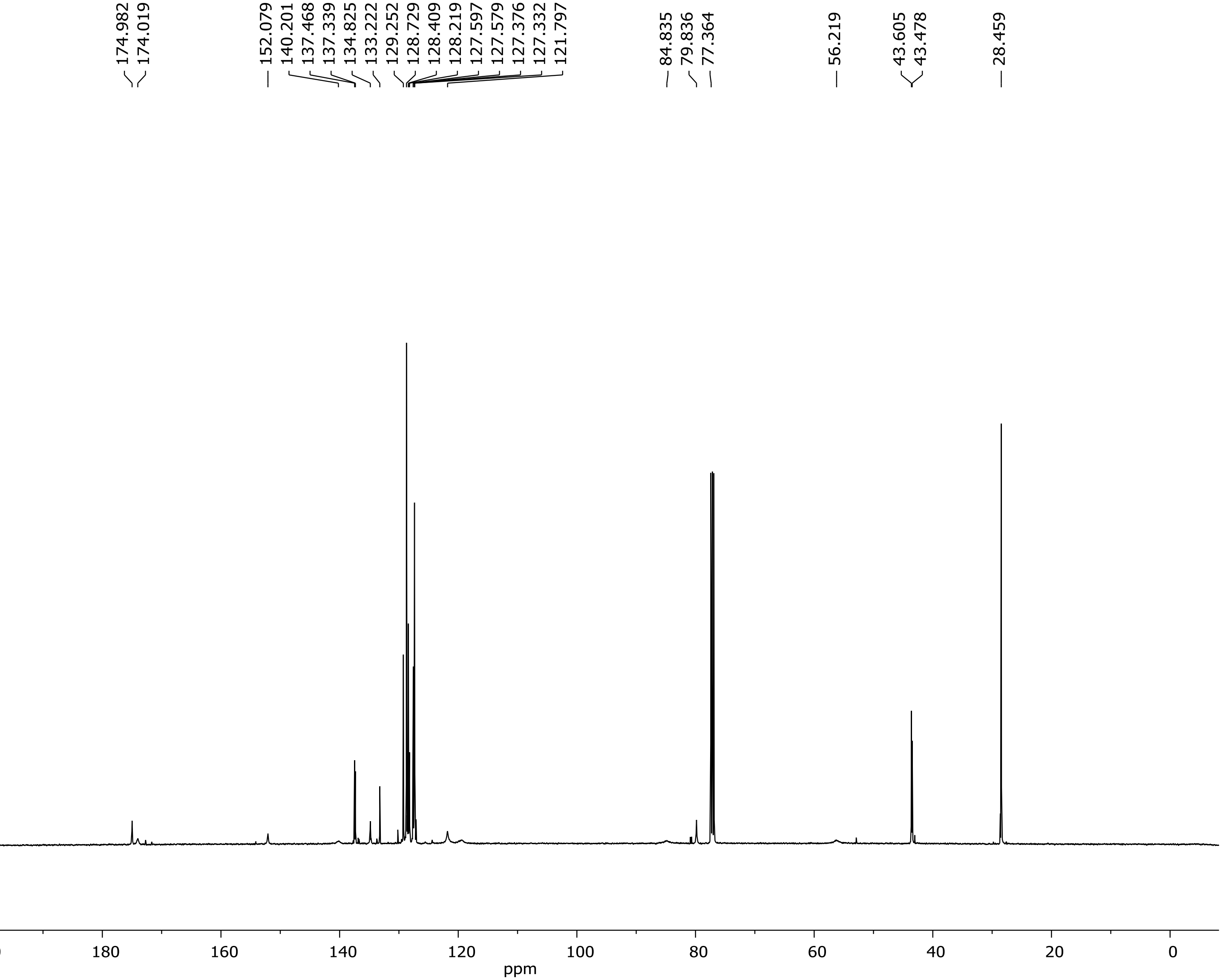

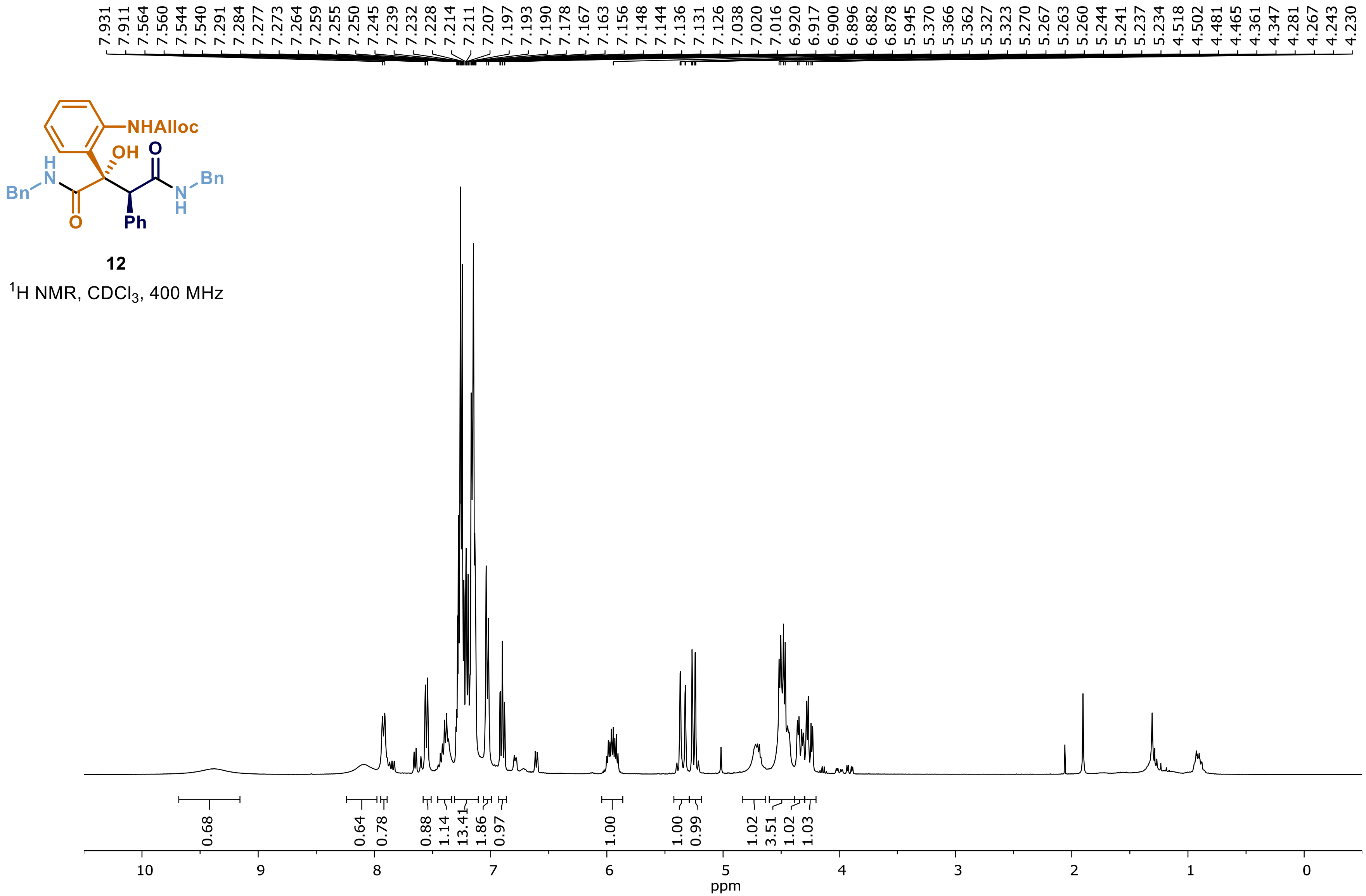
NHAlloc
OH
O
H
N
Bn
Bn
N
H
O
Ph
12
1H NMR, CDCl3, 400 MHz
ppm

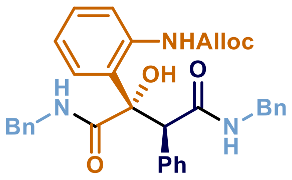


**12**

$^{13}C\{^1H\}$ NMR, $CDCl_3$, 126 MHz

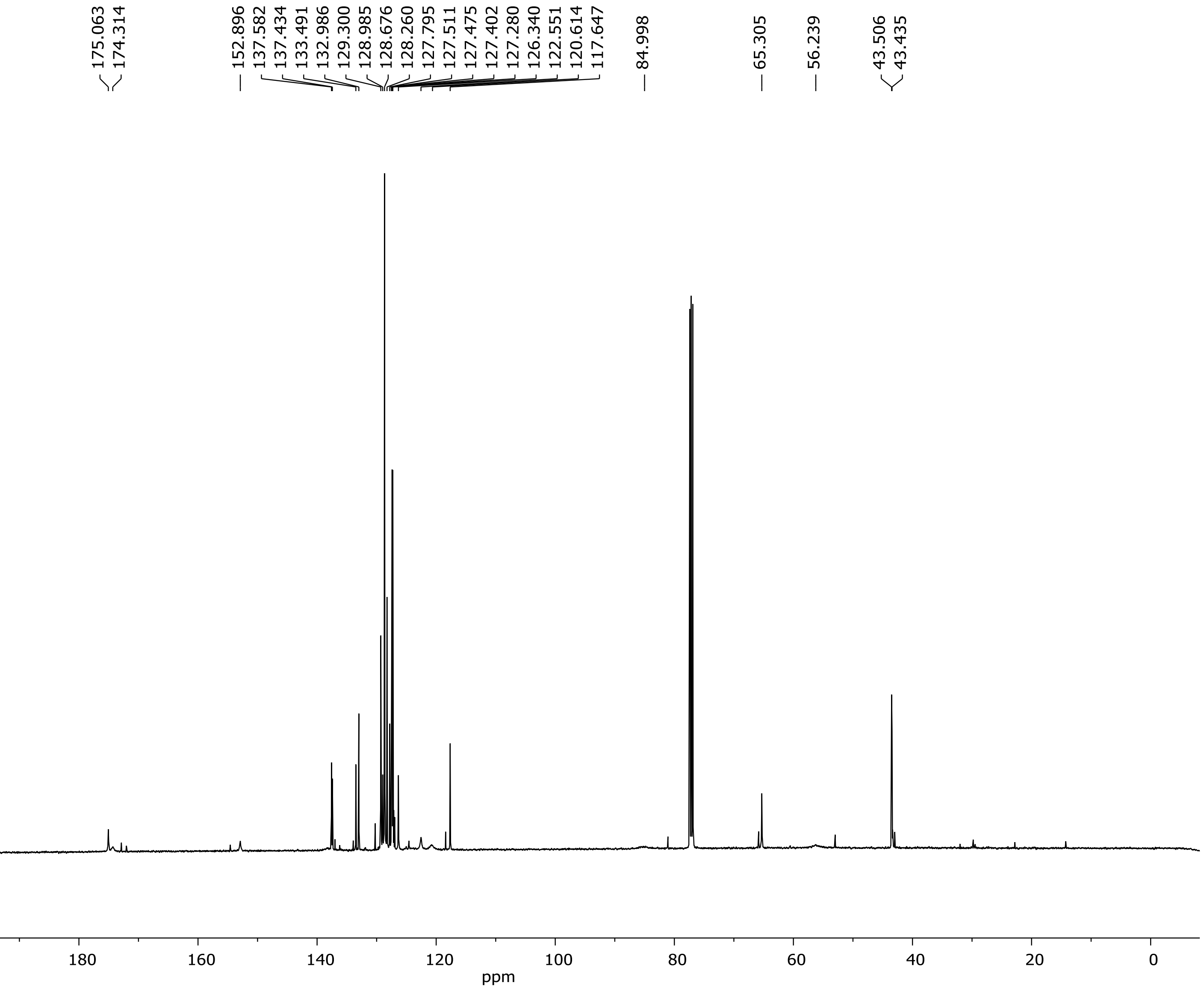

# Appendix II: HPLC traces of novel compounds

HPLC data for **1**: Chiralpak IA (95:5 *n*-hexane : $^i$PrOH, flow rate 2.0 mL·min$^{-1}$, 211 nm, 40 °C) $t_R$ (2*S*,3*S*)-**1** 23.8 min, $t_R$ (2*R*,3*R*)-**1** 28.1 min, >99:1 er.

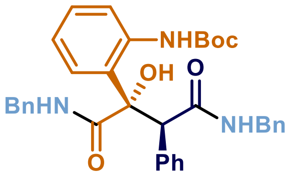


PDA Ch1 211nm

| Peak# | Ret. Time | Area% |
|---|---|---|
| 1 | 23.777 | 49.681 |
| 2 | 28.106 | 50.319 |
| Total | | 100.000 |

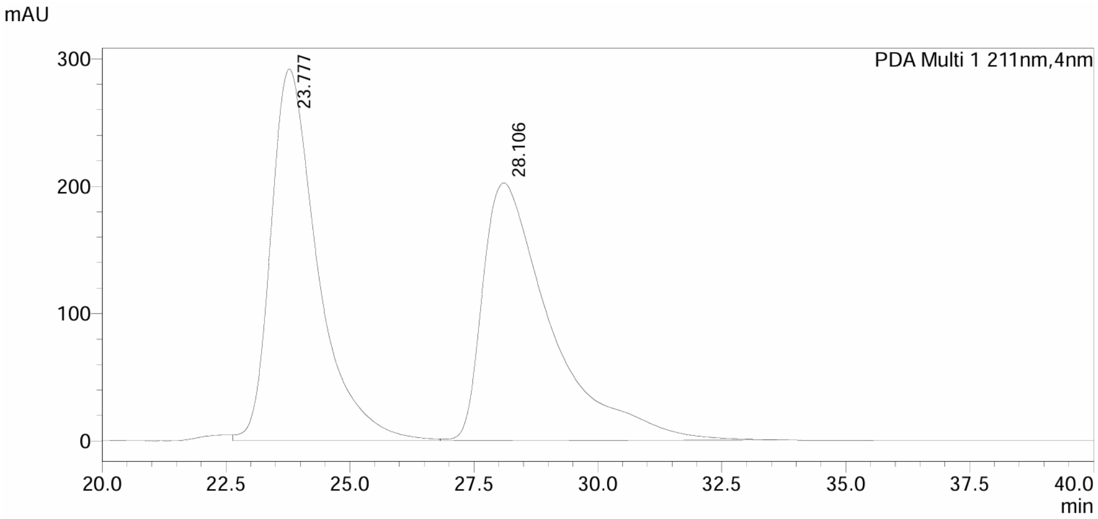


PDA Ch1 211nm

| Peak# | Ret. Time | Area% |
|---|---|---|
| 1 | 23.660 | 100.009 |
| 2 | 28.247 | -0.009 |
| Total | | 100.000 |

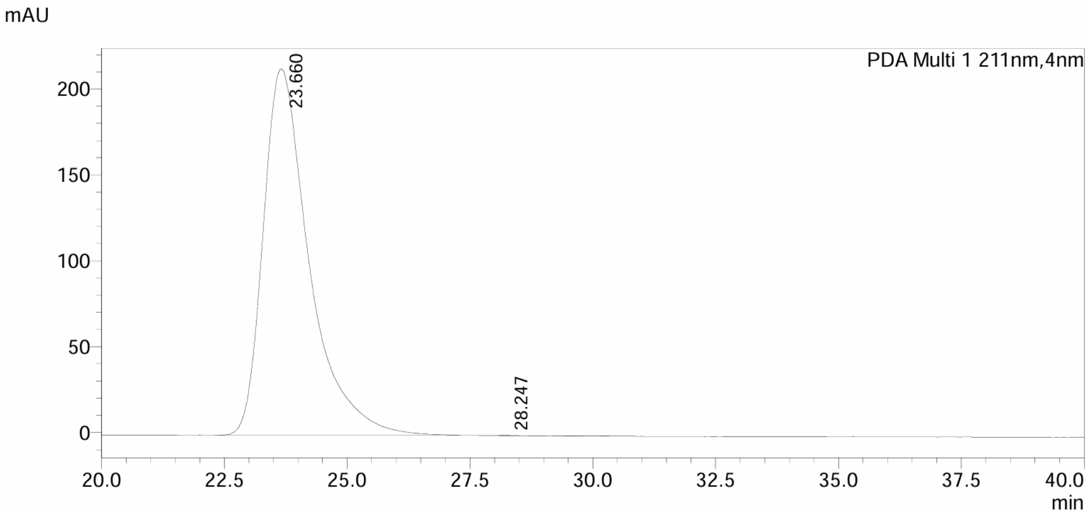

HPLC data for **2**: Chiralpak IA (80:20 *n*-hexane : $^{i}$PrOH, flow rate 1.0 mL·min$^{-1}$, 270 nm, 30 °C) $t_R$ (2*R*,3*R*)-**2** 15.9 min, $t_R$ (2*S*,3*S*)-**2** 18.5 min, 98:2 er.

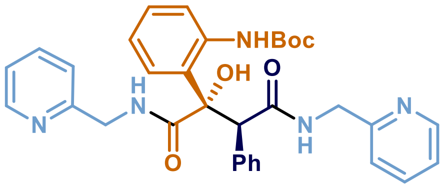


PDA Ch4 270nm

| Peak# | Ret. Time | Area% |
|---|---|---|
| 1 | 15.544 | 49.279 |
| 2 | 19.332 | 50.721 |
| Total | | 100.000 |

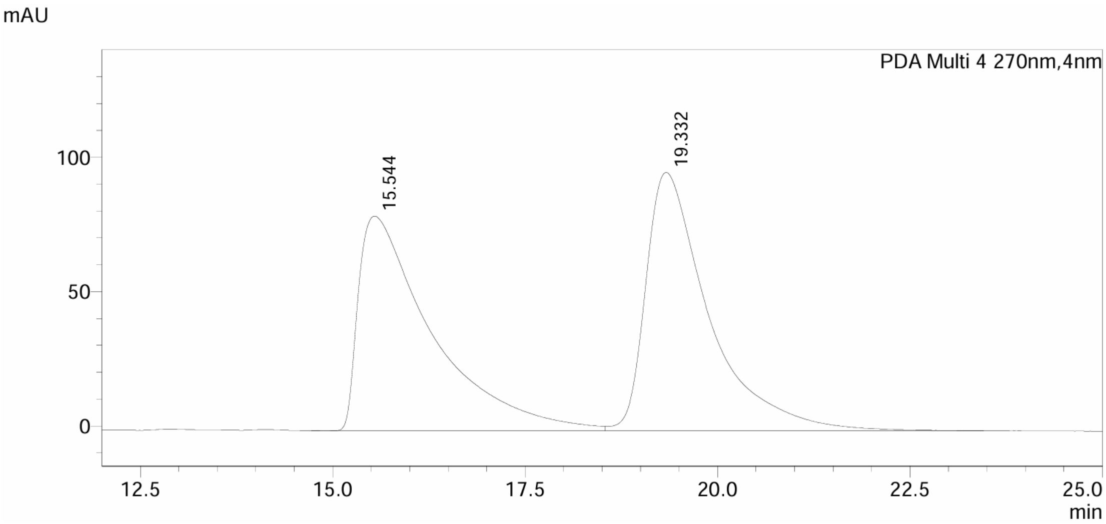


PDA Ch4 270nm

| Peak# | Ret. Time | Area% |
|---|---|---|
| 1 | 15.919 | 1.926 |
| 2 | 18.530 | 98.074 |
| Total | | 100.000 |

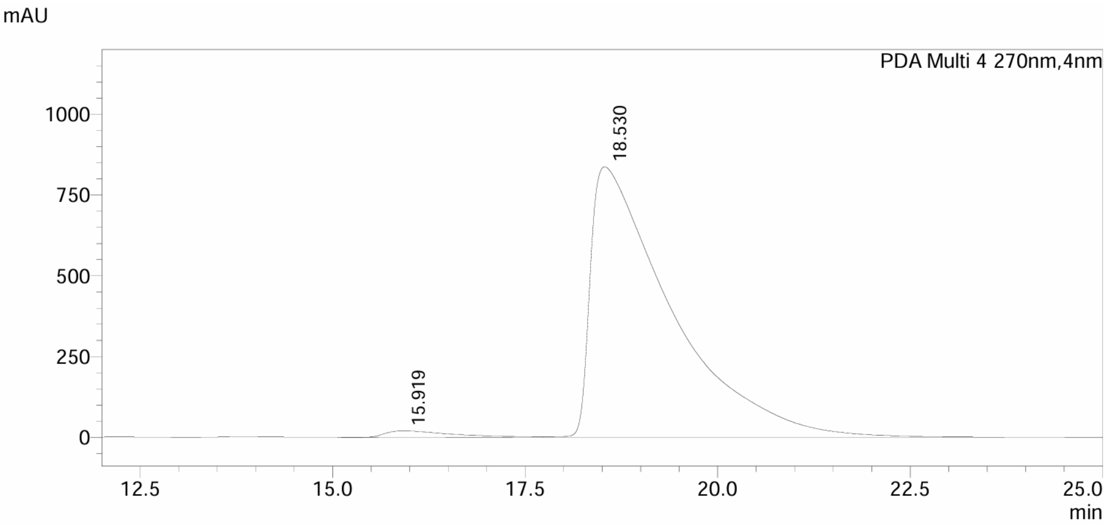

HPLC data for **3**: Chiralpak IA (98:2 *n*-hexane : $^{i}$PrOH, flow rate 1.5 mL·min$^{-1}$, 254 nm, 40 °C) $t_R$ (2*S*,3*S*)-**3** 10.9 min, $t_R$ (2*R*,3*R*)-**3** 21.3 min, 98:2 er.

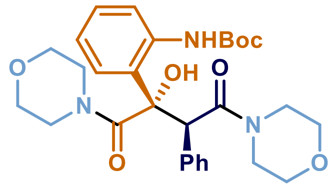


PDA Ch2 220nm

| Peak# | Ret. Time | Area% |
|---|---|---|
| 1 | 11.103 | 49.692 |
| 2 | 20.886 | 50.308 |
| Total | | 100.000 |

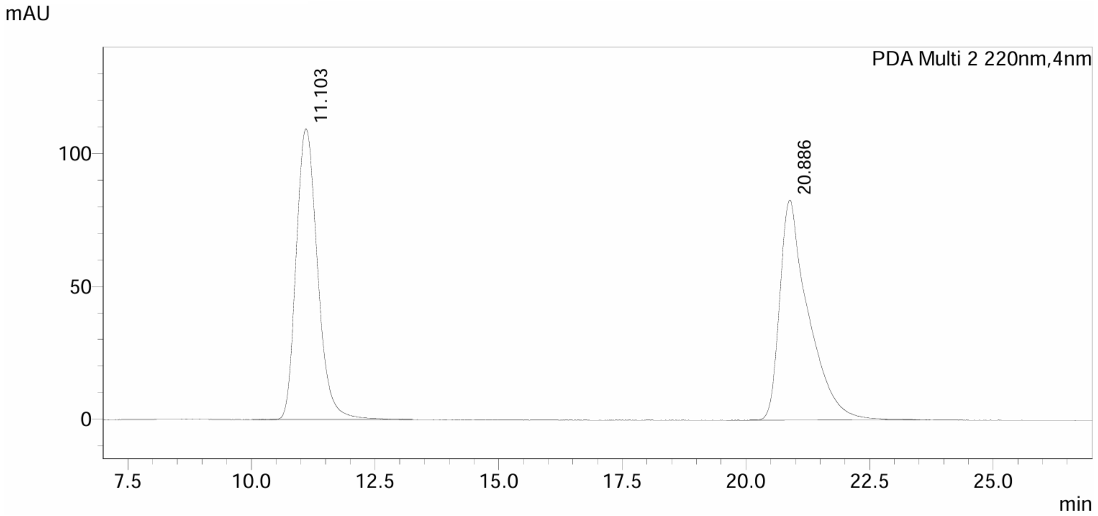


PDA Ch2 220nm

| Peak# | Ret. Time | Area% |
|---|---|---|
| 1 | 10.931 | 98.375 |
| 2 | 21.274 | 1.625 |
| Total | | 100.000 |

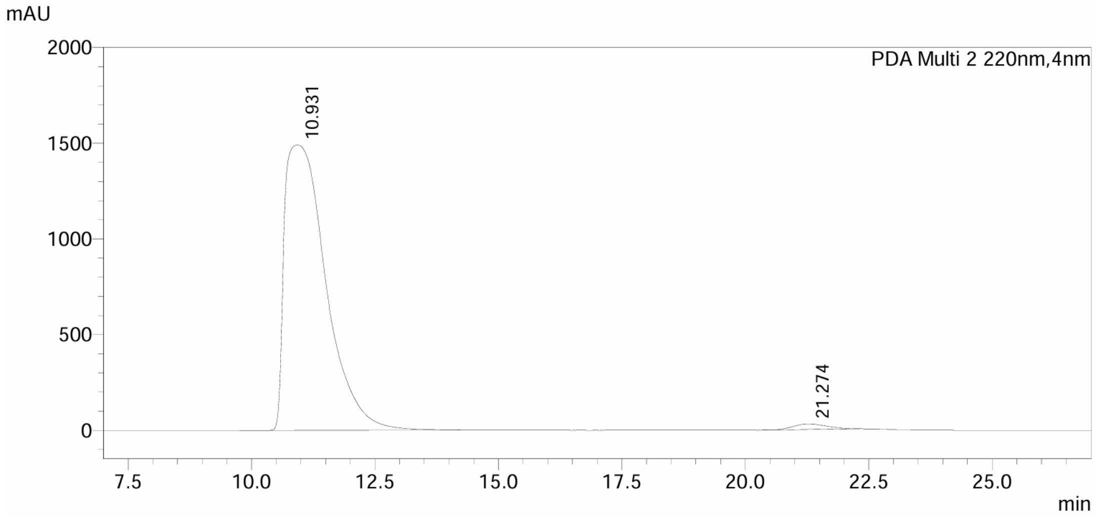

HPLC data for **4**: Chiralpak IA (70:30 *n*-hexane : $^i$PrOH, flow rate 1.0 mL·min$^{-1}$, 254 nm, 30 °C) $t_R$ (2*S*,3*S*)-**4** 6.7 min, $t_R$ (2*R*,3*R*)-**4** 12.7 min, 99:1 er.

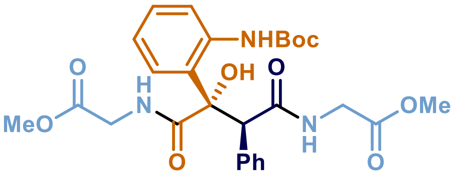


PDA Ch3 254nm

| Peak# | Ret. Time | Area% |
|---|---|---|
| 1 | 6.748 | 50.136 |
| 2 | 12.615 | 49.864 |
| Total | | 100.000 |

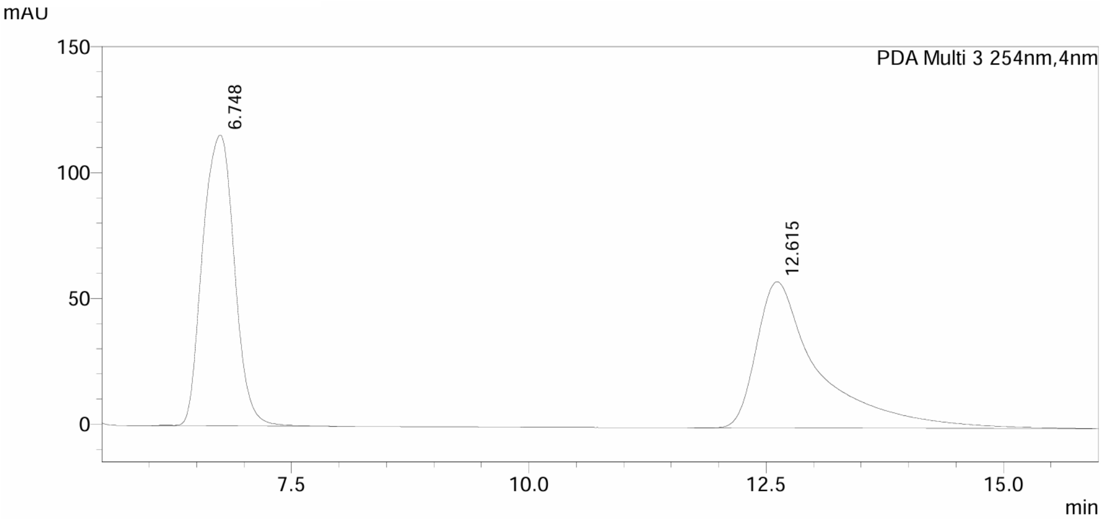


PDA Ch3 254nm

| Peak# | Ret. Time | Area% |
|---|---|---|
| 1 | 6.752 | 98.763 |
| 2 | 12.672 | 1.237 |
| Total | | 100.000 |

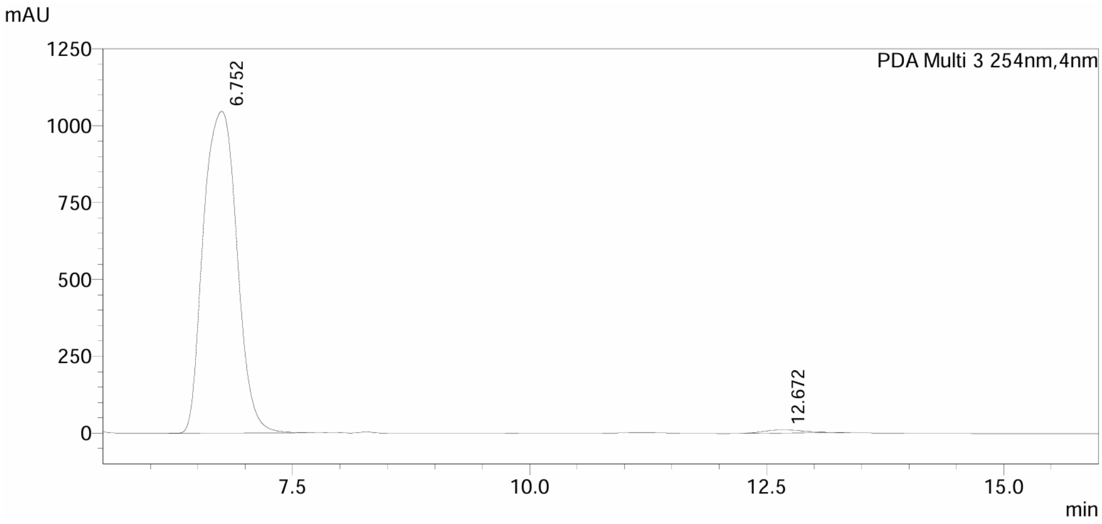

HPLC data for **5**: Chiralpak IA (98:2 *n*-hexane : $^i$PrOH, flow rate 1.5 mL·min$^{-1}$, 254 nm, 40 °C) $t_R$ (2*S*,3*S*)-**5** 12.3 min, $t_R$ (2*R*,3*R*)-**5** 28.1 min, 99:1 er.

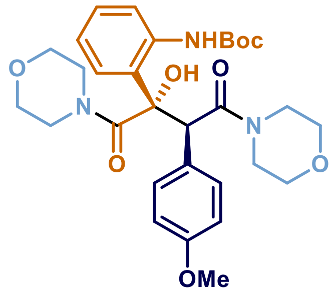


PDA Ch3 254nm

| Peak# | Ret. Time | Area% |
|---|---|---|
| 1 | 12.297 | 50.030 |
| 2 | 27.176 | 49.970 |
| Total | | 100.000 |

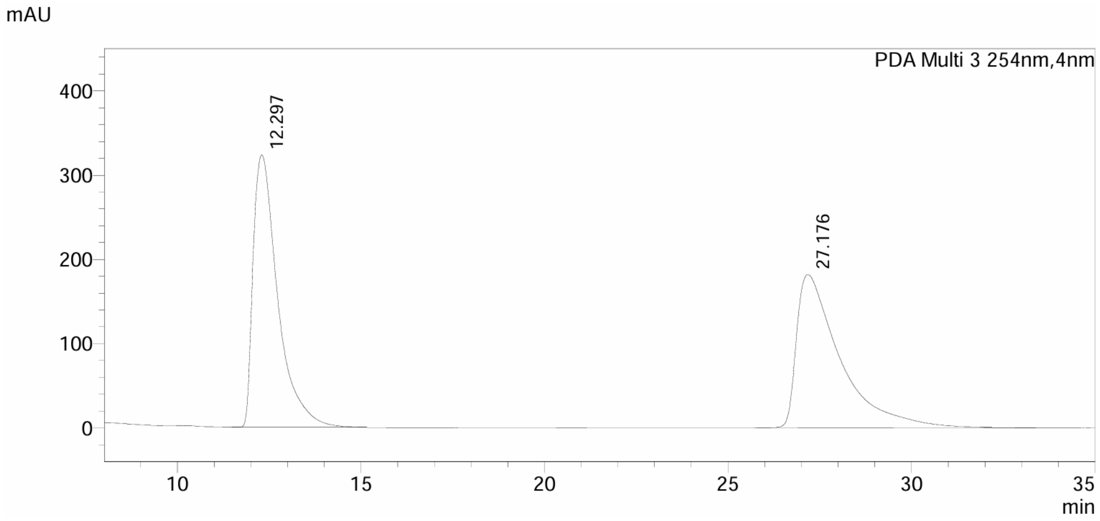


PDA Ch3 254nm

| Peak# | Ret. Time | Area% |
|---|---|---|
| 1 | 12.289 | 98.917 |
| 2 | 28.135 | 1.083 |
| Total | | 100.000 |

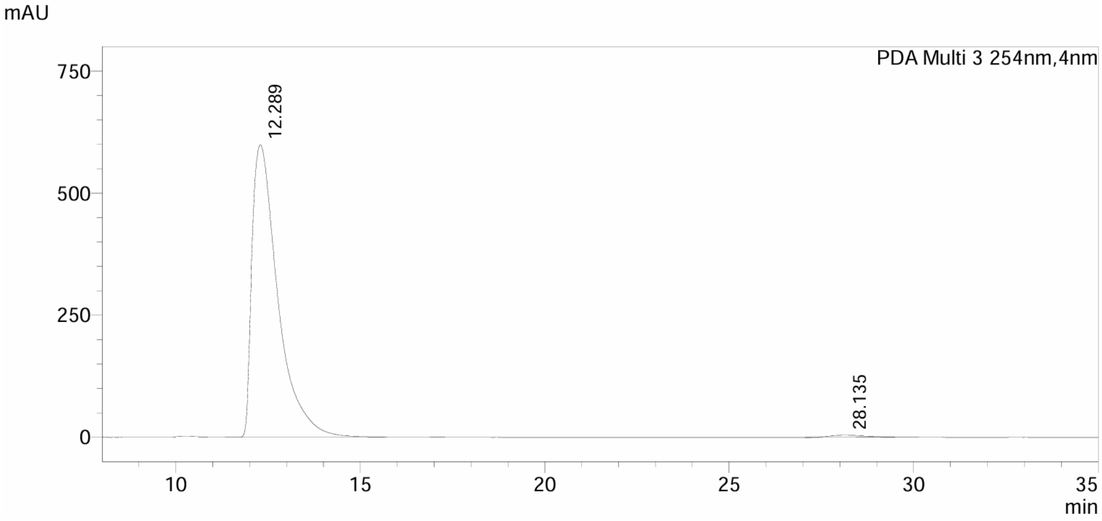

HPLC data for **6**: Chiralpak AD-H (70:30 *n*-hexane : *i*PrOH, flow rate 1.0 mL·min$^{-1}$, 211 nm, 30 °C) tR (2*R*,3*R*)-**6** 9.7 min, tR (2*S*,3*S*)-**6** 19.7 min, 97:3 er; tR (2*S*,3*R*)-**6** 11.4 min, tR (2*R*,3*S*)-**6** 15.0 min, 77:23 er.

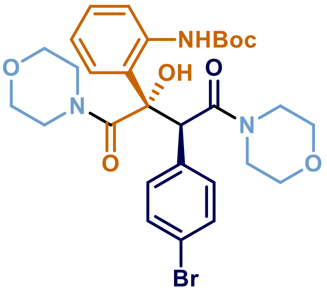


PDA Ch1 211nm

| Peak# | Ret. Time | Area% |
|---|---|---|
| 1 | 9.604 | 41.611 |
| 2 | 11.403 | 7.063 |
| 3 | 15.060 | 6.285 |
| 4 | 19.851 | 45.042 |
| Total | | 100.000 |

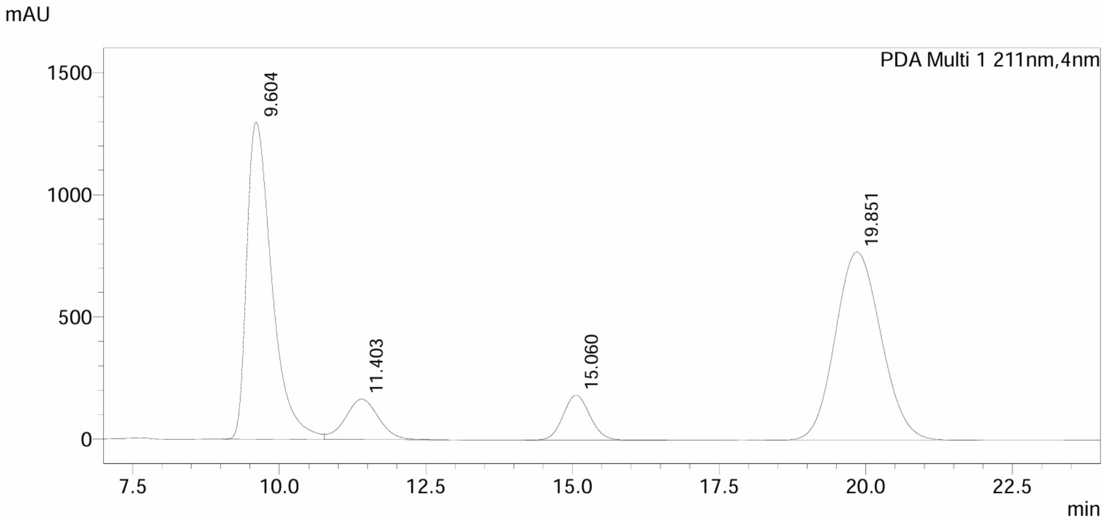


PDA Ch1 211nm

| Peak# | Ret. Time | Area% |
|---|---|---|
| 1 | 9.689 | 2.423 |
| 2 | 11.403 | 18.180 |
| 3 | 15.032 | 5.569 |
| 4 | 19.731 | 73.828 |
| Total | | 100.000 |

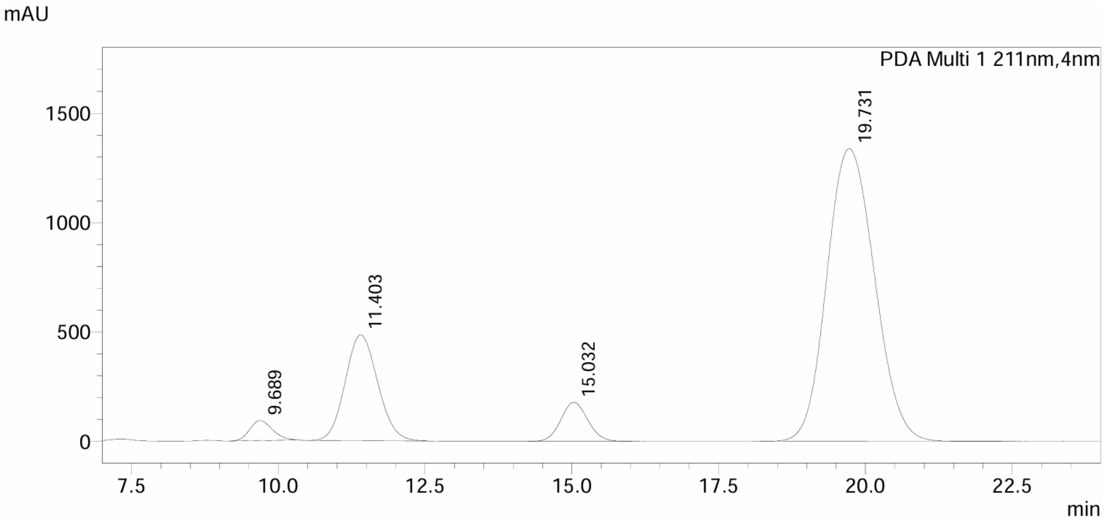

HPLC data for **7**: Chiralpak IA (98:2 *n*-hexane : $^{i}$PrOH, flow rate 1.5 mL·min$^{-1}$, 211 nm, 40 °C) $t_R$ (2*S*,3*S*)-**7** 13.1 min, $t_R$ (2*R*,3*R*)-**7** 26.5 min, 98:2 er.

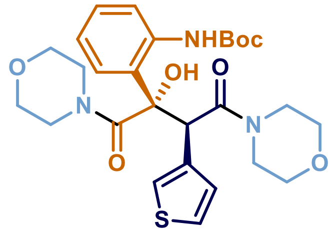


PDA Ch1 211nm

| Peak# | Ret. Time | Area% |
|---|---|---|
| 1 | 13.354 | 50.667 |
| 2 | 26.224 | 49.333 |
| Total | | 100.000 |

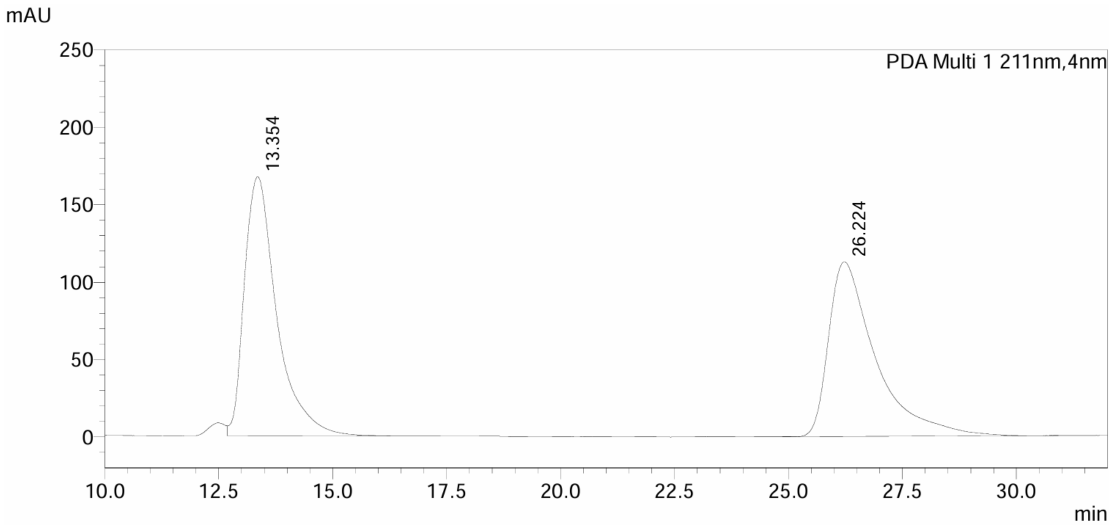


PDA Ch1 211nm

| Peak# | Ret. Time | Area% |
|---|---|---|
| 1 | 13.140 | 98.093 |
| 2 | 26.519 | 1.907 |
| Total | | 100.000 |

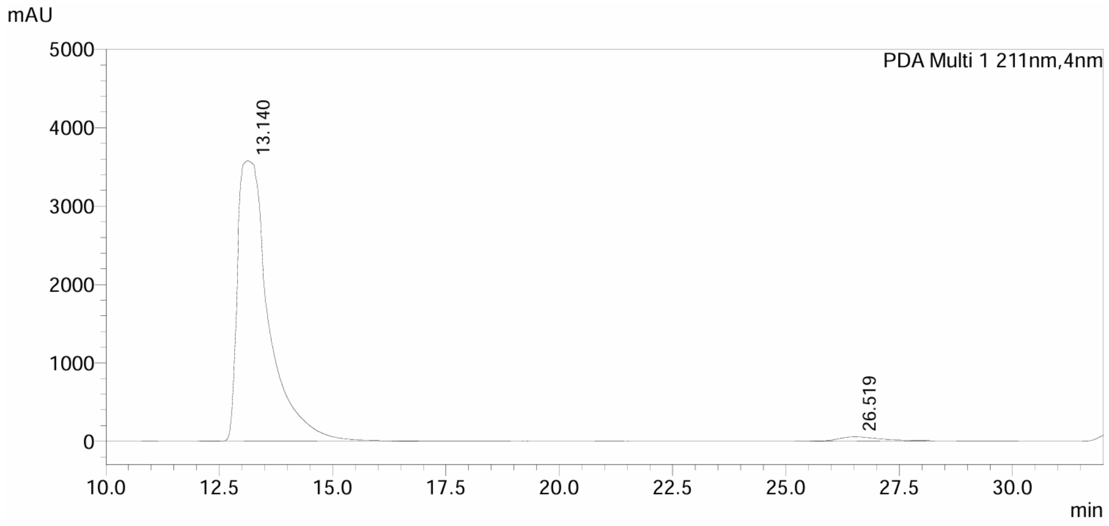

HPLC data for **8**: Chiralpak IA (97:3 *n*-hexane : $^{i}$PrOH, flow rate 1.0 mL·min$^{-1}$, 211 nm, 40 °C) $t_R$ (2*S*,3*S*)-**8** 30.6 min, $t_R$ (2*R*,3*R*)-**8** 34.4 min, 97:3 er.

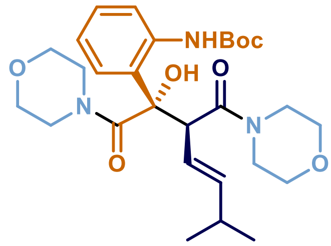


PDA Ch1 211nm

| Peak# | Ret. Time | Area% |
|---|---|---|
| 1 | 31.077 | 49.719 |
| 2 | 34.568 | 50.281 |
| Total | | 100.000 |

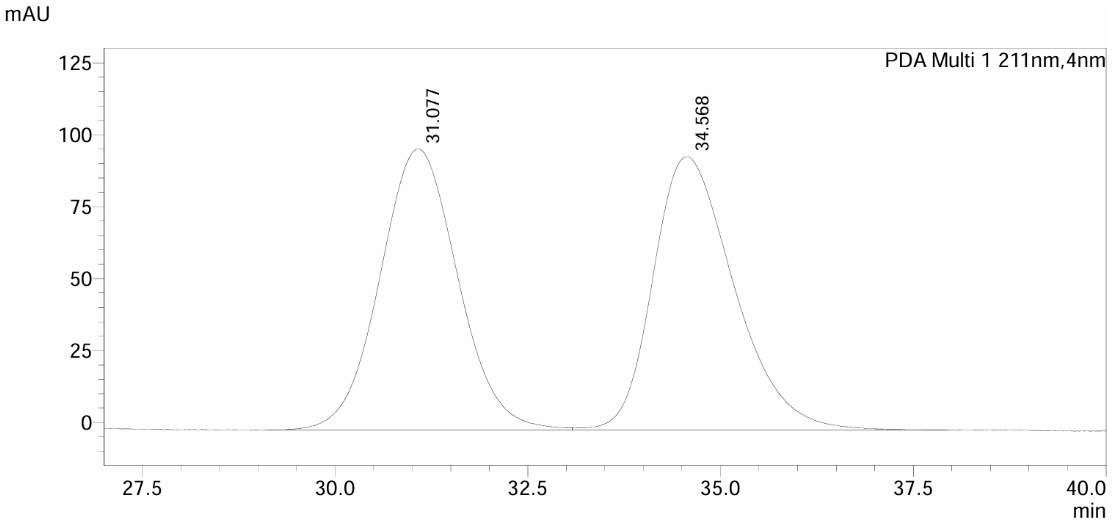


PDA Ch1 211nm

| Peak# | Ret. Time | Area% |
|---|---|---|
| 1 | 30.600 | 96.748 |
| 2 | 34.366 | 3.252 |
| Total | | 100.000 |

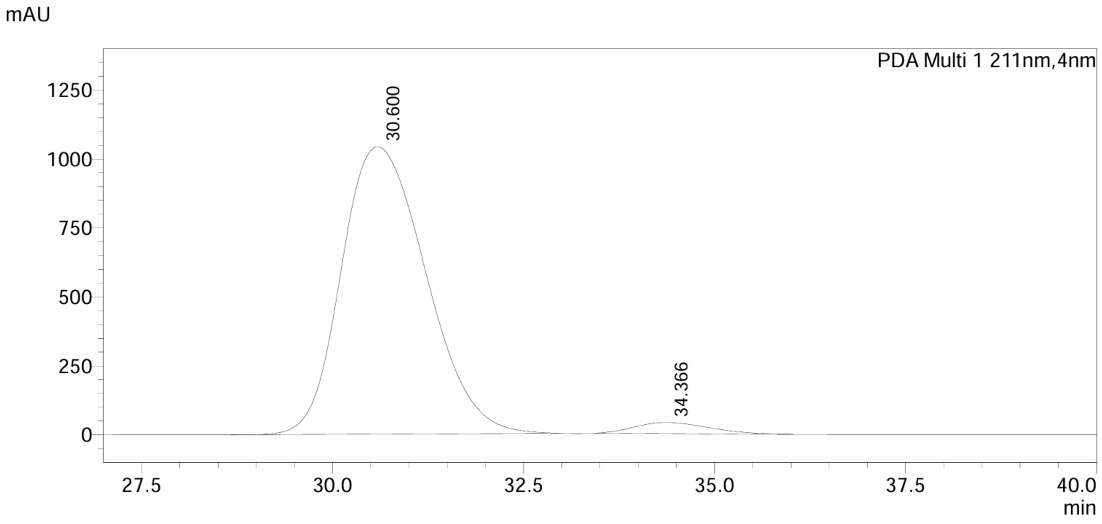

HPLC data for **9**: Chiralpak IA (90:10 *n*-hexane : $^{i}$PrOH, flow rate 1.0 mL·min$^{-1}$, 211 nm, 30 °C) $t_R$ (2*S*,3*S*)-**9** 29.0 min, $t_R$ (2*R*,3*R*)-**9** 46.5 min, >99:1 er.

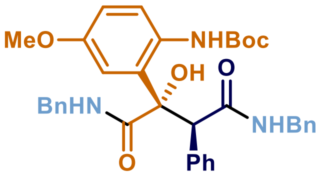


PDA Ch1 211nm

| Peak# | Ret. Time | Area% |
|---|---|---|
| 1 | 29.062 | 50.119 |
| 2 | 45.162 | 49.881 |
| Total | | 100.000 |

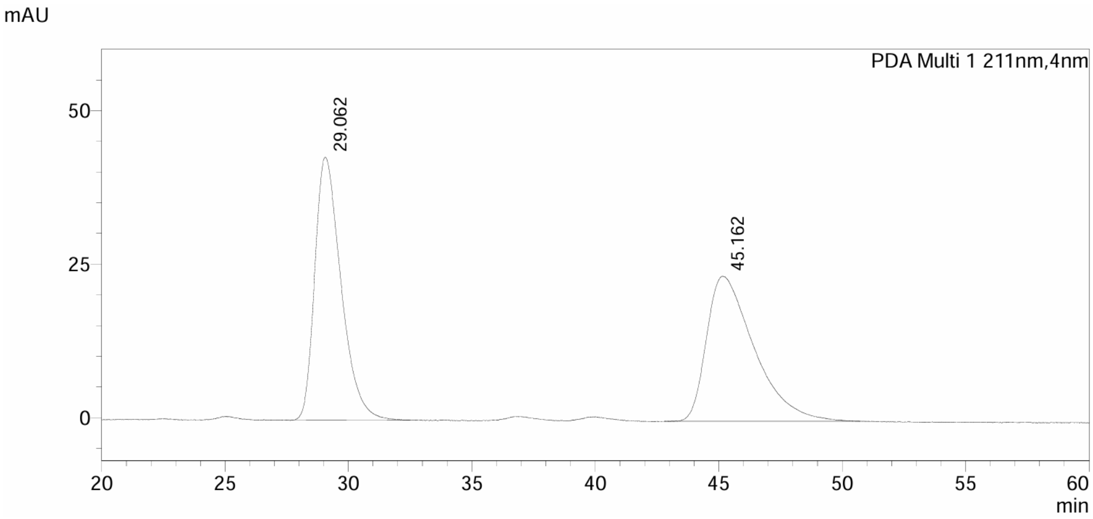


PDA Ch1 211nm

| Peak# | Ret. Time | Area% |
|---|---|---|
| 1 | 28.973 | 99.631 |
| 2 | 46.499 | 0.369 |
| Total | | 100.000 |

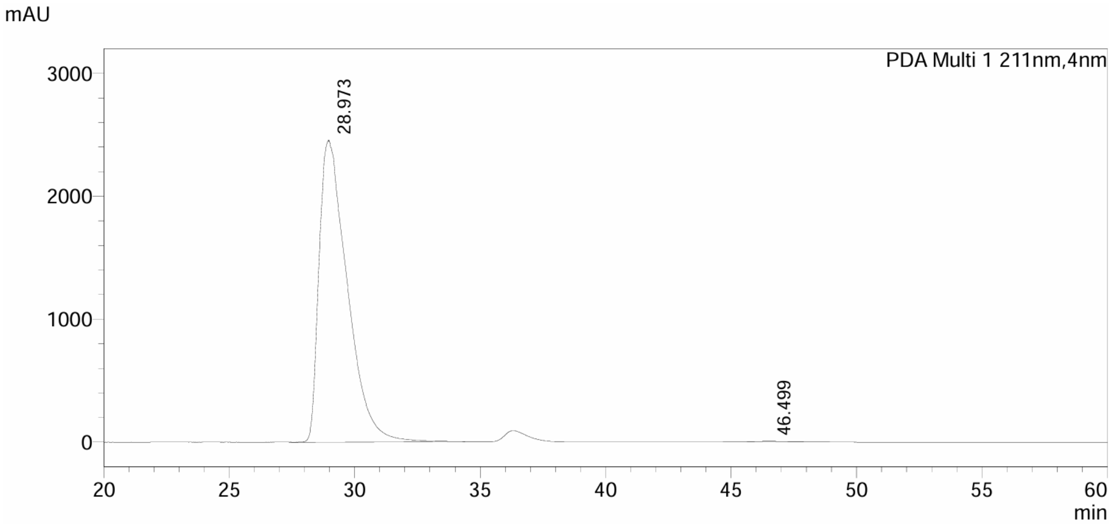

HPLC data for **10**: Chiralpak IA (90:10 *n*-hexane : $^{i}$PrOH, flow rate 1.0 mL·min$^{-1}$, 211 nm, 30 °C) $t_R$ (2*S*,3*S*)-**10** 12.2 min, $t_R$ (2*R*,3*R*)-**10** 30.7 min, 99:1 er.

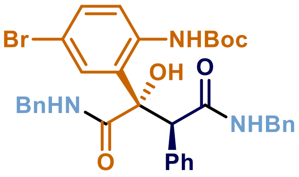


PDA Ch1 211nm

| Peak# | Ret. Time | Area% |
|---|---|---|
| 1 | 12.366 | 49.697 |
| 2 | 30.865 | 50.303 |
| Total | | 100.000 |

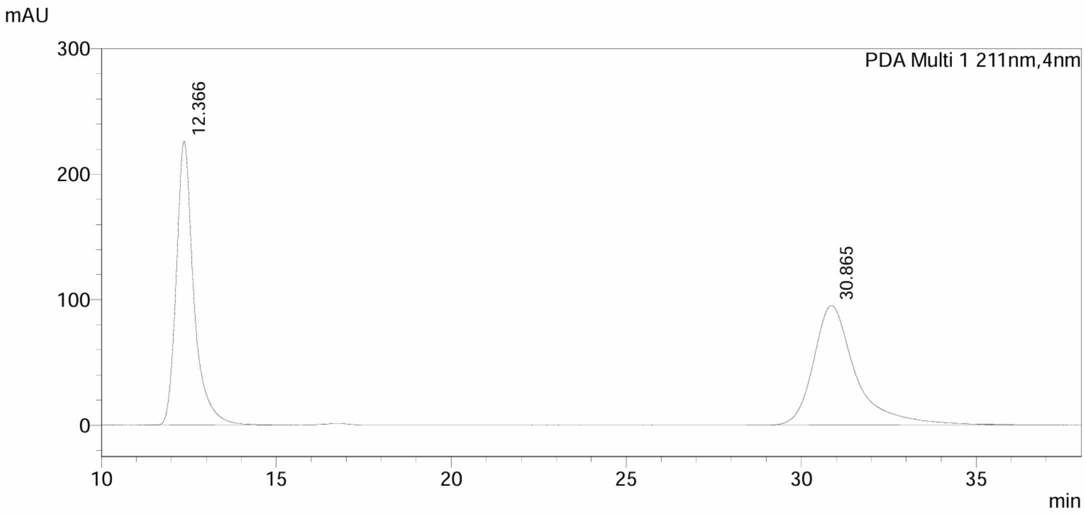


PDA Ch1 211nm

| Peak# | Ret. Time | Area% |
|---|---|---|
| 1 | 12.150 | 98.936 |
| 2 | 30.695 | 1.064 |
| Total | | 100.000 |

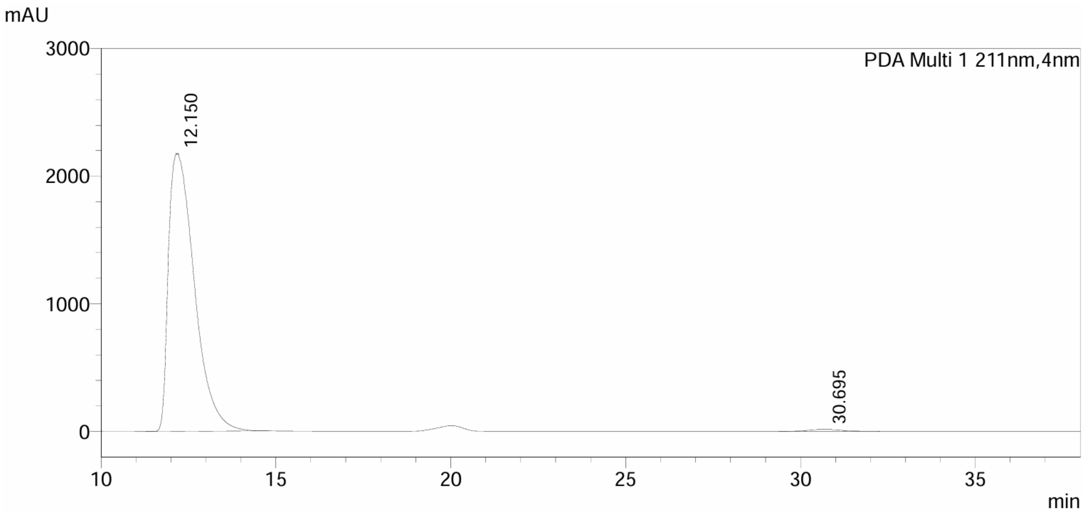

HPLC data for **11**: Chiralpak ID (90:10 *n*-hexane : $^{i}$PrOH, flow rate 1.0 mL·min$^{-1}$, 254 nm, 30 °C) $t_R$ (2*S*,3*S*)-**11** 10.1 min, $t_R$ (2*R*,3*R*)-**11** 28.6 min, 99:1 er.

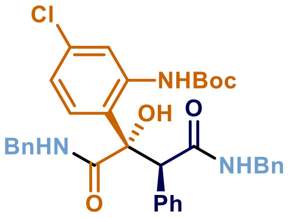


Detector A Channel 2 254nm

| Peak# | Ret. Time | Area% |
|---|---|---|
| 1 | 10.226 | 50.472 |
| 2 | 27.549 | 49.528 |
| Total | | 100.000 |

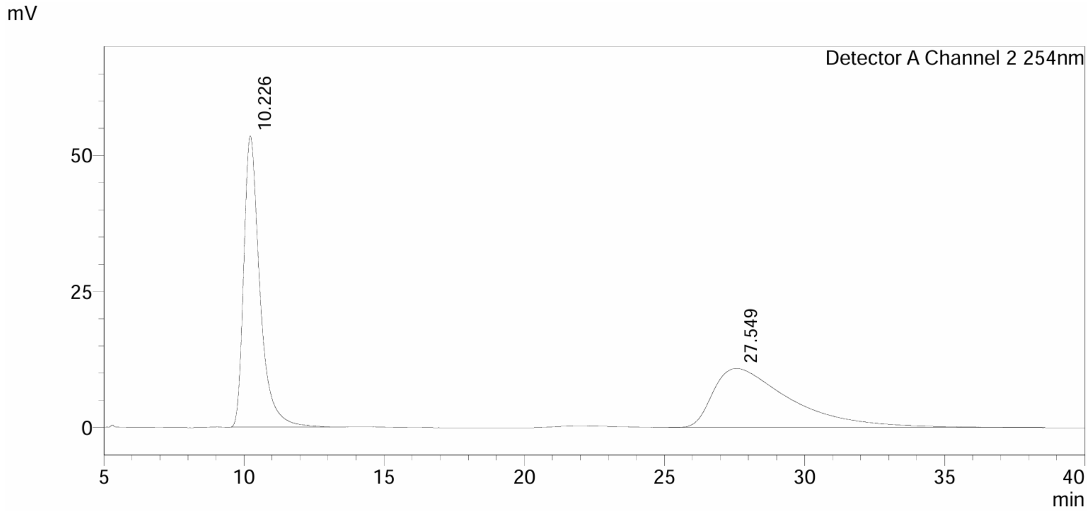


Detector A Channel 2 254nm

| Peak# | Ret. Time | Area% |
|---|---|---|
| 1 | 10.070 | 99.446 |
| 2 | 28.583 | 0.554 |
| Total | | 100.000 |

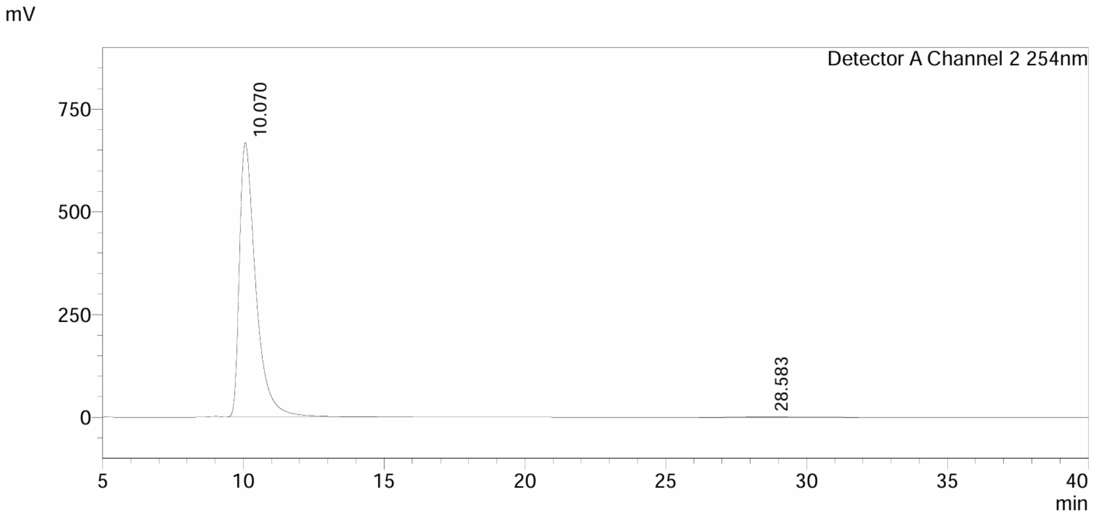

HPLC data for **12**: Chiralpak ID (60:40 *n*-hexane : $^{i}$PrOH, flow rate 1.0 mL·min$^{-1}$, 254 nm, 40 °C) $t_R$ (2*S*,3*S*)-**12** 6.4 min, $t_R$ (2*R*,3*R*)-**12** 12.3 min, >99:1 er.

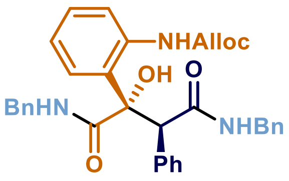


Detector A Channel 2 254nm

| Peak# | Ret. Time | Area% |
|---|---|---|
| 1 | 6.364 | 46.106 |
| 2 | 12.326 | 47.625 |
| 3 | 17.215 | 2.993 |
| 4 | 19.526 | 3.276 |
| Total | | 100.000 |

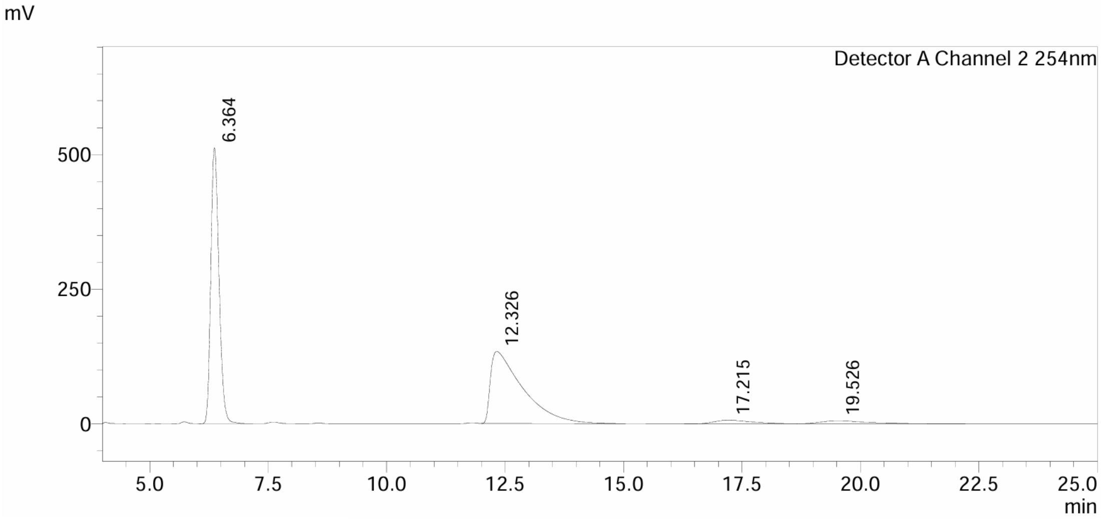


Detector A Channel 2 254nm

| Peak# | Ret. Time | Area% |
|---|---|---|
| 1 | 6.411 | 94.087 |
| 2 | 19.601 | 5.913 |
| Total | | 100.000 |

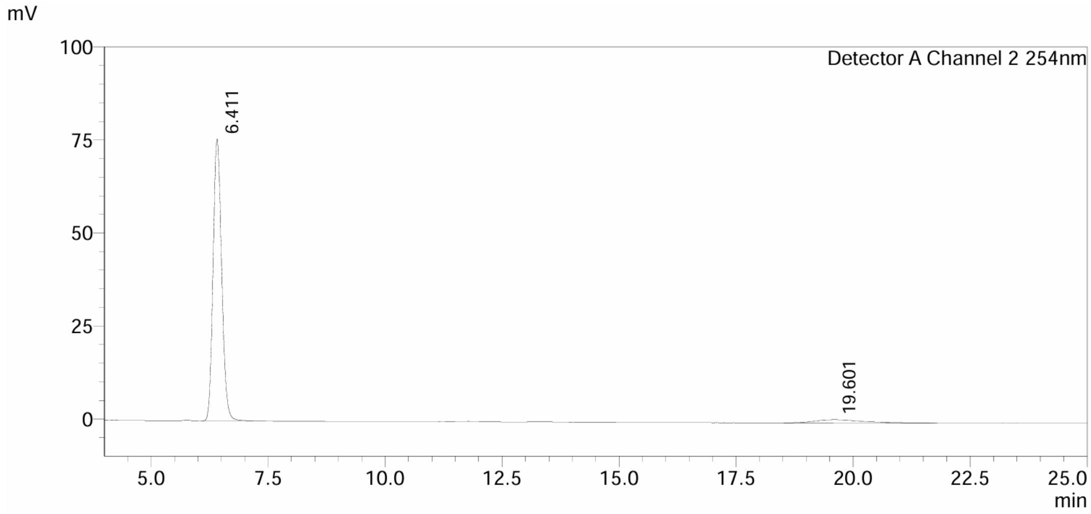

Supplement: Supplementary file 1 [file molecules-29-03635-s001.zip › molecules-3105765-supplementary.pdf]
